# Composition and Antigenic Effects of Individual Glycan Sites of a Trimeric HIV-1 Envelope Glycoprotein

**DOI:** 10.1016/j.celrep.2016.02.058

**Published:** 2016-03-10

**Authors:** Anna-Janina Behrens, Snezana Vasiljevic, Laura K. Pritchard, David J. Harvey, Rajinder S. Andev, Stefanie A. Krumm, Weston B. Struwe, Albert Cupo, Abhinav Kumar, Nicole Zitzmann, Gemma E. Seabright, Holger B. Kramer, Daniel I.R. Spencer, Louise Royle, Jeong Hyun Lee, Per J. Klasse, Dennis R. Burton, Ian A. Wilson, Andrew B. Ward, Rogier W. Sanders, John P. Moore, Katie J. Doores, Max Crispin

**Affiliations:** 1 Oxford Glycobiology Institute and Department of Biochemistry, University of Oxford, South Parks Road, Oxford OX1 3QU, UK; 2 Department of Infectious Diseases, Faculty of Life Sciences and Medicine, King's College London, Guy's Hospital, London SE1 9RT, UK; 3 Department of Microbiology and Immunology, Weill Cornell Medical College, New York, NY 10021, USA; 4 Department of Physiology, Anatomy and Genetics, University of Oxford, South Parks Road, Oxford OX1 3QX, UK; 5 Ludger, Ltd., Culham Science Centre, Abingdon, Oxfordshire OX14 3EB, UK; 6 Department of Integrative Structural and Computational Biology, International AIDS Vaccine Initiative (IAVI) Neutralizing Antibody Center and CAVD, Center for HIV/AIDS Vaccine Immunology and Immunogen Discovery, The Scripps Research Institute, La Jolla, CA 92037, USA; 7 Department of Immunology and Microbial Science, IAVI Neutralizing Antibody Center and CAVD, Center for HIV/AIDS Vaccine Immunology and Immunogen Discovery, the Scripps Research Institute, La Jolla, CA 92037, USA; 8 Ragon Institute of Massachusetts General Hospital, Massachusetts Institute of Technology and Harvard University, Boston, MA 02142, USA; 9 Skaggs Institute for Chemical Biology, The Scripps Research Institute, La Jolla, CA 92037, USA; 10 Laboratory of Experimental Virology, Department of Medical Microbiology, Center for Infection and Immunity Amsterdam (CINIMA), Academic Medical Center of the University of Amsterdam, 1105 AZ Amsterdam, the Netherlands

## Abstract

The HIV-1 envelope glycoprotein trimer is covered by an array of N-linked glycans that shield it from immune surveillance. The high density of glycans on the trimer surface imposes steric constraints limiting the actions of glycan-processing enzymes, so that multiple under-processed structures remain on specific areas. These oligomannose glycans are recognized by broadly neutralizing antibodies (bNAbs) that are not thwarted by the glycan shield but, paradoxically, target it. Our site-specific glycosylation analysis of a soluble, recombinant trimer (BG505 SOSIP.664) maps the extremes of simplicity and diversity of glycan processing at individual sites and reveals a mosaic of dense clusters of oligomannose glycans on the outer domain. Although individual sites usually minimally affect the global integrity of the glycan shield, we identify examples of how deleting some glycans can subtly influence neutralization by bNAbs that bind at distant sites. The network of bNAb-targeted glycans should be preserved on vaccine antigens.

## Highlights

• Quantitative, site-specific N-glycan analysis of a soluble HIV-1 Env trimer
• A map of the extremes of simplicity and diversity at individual glycan sites
• The fine structure of the mannose patch area of the Env trimer
• How individual glycan sites influence HIV-1 Env-pseudovirus neutralization

## Graphical Abstract

**Figure:**
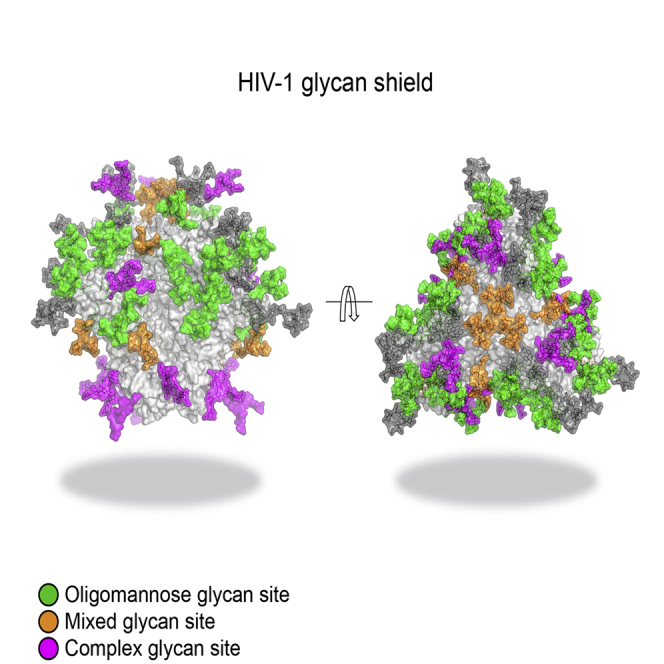

## Teaser

Behrens et al. present detailed, quantitative, site-specific analyses of N-glycosylation sites of a soluble recombinant HIV-1 envelope glycoprotein trimer. The results highlight structural and antigenic details of the glycan shield that will be valuable for designing next-generation HIV-1 Env vaccines and understanding virus neutralization by broadly active antibodies.

## Introduction

The trimeric HIV type 1 (HIV-1) envelope glycoprotein (Env) is the sole target for broadly neutralizing antibodies (bNAbs) produced by the immune system during infection and is, therefore, a focus of vaccine design. In numerous studies, bNAbs provide passive protection from viral challenge to non-human primates (Hessell et al., 2010, Mascola et al., 2000, Moldt et al., 2012). Many of these bNAbs recognize epitopes that are wholly or partially composed of glycan structures (Blattner et al., 2014, Calarese et al., 2003, Falkowska et al., 2014, Garces et al., 2014, Huang et al., 2014, Kong et al., 2013, McLellan et al., 2011, Mouquet et al., 2012, Pancera et al., 2013, Pejchal et al., 2011, Scanlan et al., 2002, Scharf et al., 2014, Walker et al., 2011). HIV-1 Env is among the most heavily glycosylated proteins known, with glycans making up ∼50% of its total mass (Lasky et al., 1986). These abundant glycans have long been considered to shield the trimer from immune surveillance by occluding relatively conserved protein surfaces (Wei et al., 2003); while this concept remains valid, it is also now evident that the glycan shield itself can be a target for bNAbs. Defining the detailed composition of the glycan shield will increase our understanding of bNAb epitopes and how HIV-1 is neutralized and, thus, help the rational design of Env-based vaccine immunogens.

The Env trimer is composed of three gp120 and three gp41 subunits. Analyses of monomeric gp120 proteins have revealed the presence of under-processed N-glycans that remain in oligomannose form (Man_5-9_GlcNAc_2_) because steric constraints impede the actions of the endoplasmic reticulum (ER) and Golgi α-mannosidases (Bonomelli et al., 2011, Doores et al., 2010, Go et al., 2013, Leonard et al., 1990, Zhu et al., 2000). These oligomannose-type glycans are mainly localized to a highly conserved area of the gp120 outer domain, the so-called “intrinsic mannose patch,” that presents numerous bNAb epitopes (Calarese et al., 2003, Doores, 2015, Doores et al., 2015, Kong et al., 2013, Pritchard et al., 2015c, Scanlan et al., 2002, Walker et al., 2009, Walker et al., 2011). Because these studies were performed using recombinant gp120 monomers, it was uncertain at the time to what extent their glycan content mimicked the native, trimeric structure on HIV-1 virions (Coutu and Finzi, 2015, Klasse et al., 2013, Kovacs et al., 2014, Ringe et al., 2013, Ringe et al., 2015). Virion-derived trimers are hard to obtain in sufficient quantities for detailed characterizations, but oligomannose-rich regions are known to be present on both Envs extracted from HIV-1 virions (Bonomelli et al., 2011, Doores et al., 2010, Pritchard et al., 2015a) and membrane-associated recombinant Env (Go et al., 2015). Nonetheless, major knowledge gaps remained to be filled.

The soluble, recombinant BG505 SOSIP.664 trimer is the prototype of a class of native-like, Env-mimetic immunogens that is now being pursued in various vaccine-development programs (Sanders et al., 2013, Sanders et al., 2015). On the BG505 SOSIP.664 trimers, the glycosylation profile of the gp120 subunits is dominated by large, oligomannose-type structures of the Man_8-9_GlcNAc_2_ type, while more complex-type structures are found on gp41 (Pritchard et al., 2015d). The native-like quaternary structure of the SOSIP.664 trimer had a major influence on its oligomannose-rich glycosylation profile; simpler (gp120 monomer) or non-native (uncleaved gp140) Env proteins carry a much higher content of processed glycans (Pritchard et al., 2015d, Ringe et al., 2015). These findings led to the concept of the “trimer-associated mannose patch” (TAMP) (Crispin and Doores, 2015).

Here, a quantitative, site-specific N-glycosylation analysis reveals the fine structures of the glycan shield of the BG505 SOSIP.664 trimer. Our results confirm the remarkable overall dominance of oligomannose-type glycans and reveal a mosaic of glycan microclusters bearing under-processed glycans, especially in areas covering the gp120 outer domain and at the trimer interfaces. At the trimer apex, there is a microcluster of glycans of mixed processing states, with both under-processed and complex structures present. In contrast, highly variable, but also highly processed, complex glycans occupy the sites present on gp41 and on the most proximal gp120 regions at the trimer base. Deleting specific potential N-glycosylation sites (PNGSs) does not markedly affect the overall glycosylation profile of the BG505 SOSIP.664 trimer but can directly or indirectly influence the neutralization sensitivity of HIV-1 BG505 Env-pseudotyped viruses.

## Results and Discussion

### A Glycan Library from BG505 SOSIP.664 Trimers

**Figure 1:**
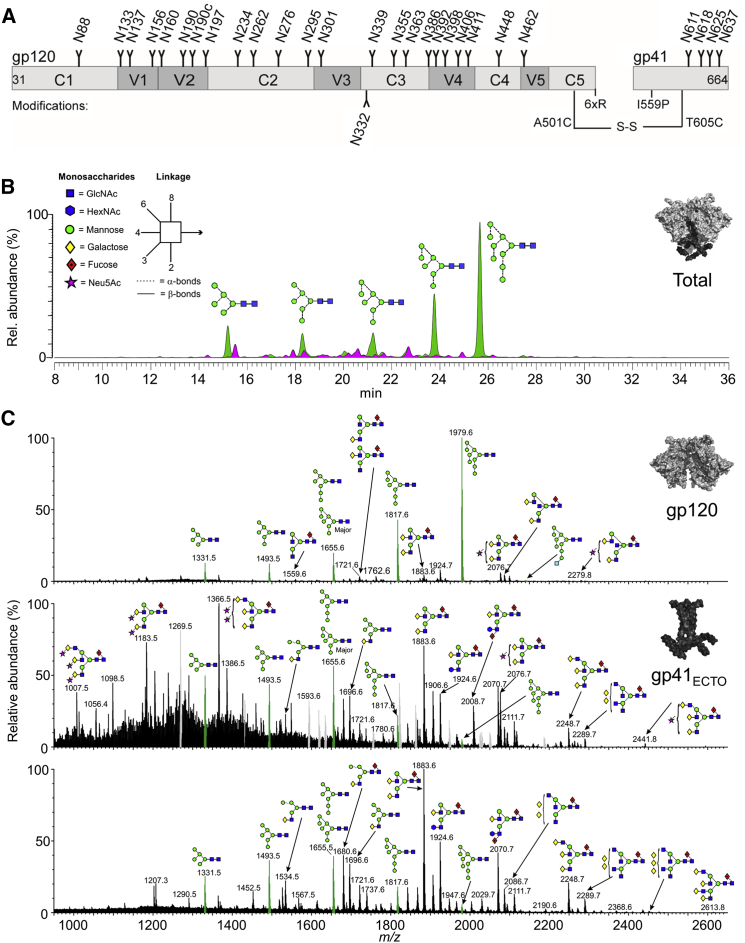
Glycosylation Pattern of BG505 SOSIP.664 Trimers (A) Schematic representation of the BG505 SOSIP.664 construct, including PNGSs and incorporated modifications. (B) HILIC-UPLC profile of N-linked glycans from BG505 SOSIP.664 trimers stably produced in HEK293T cells and purified by 2G12-affinity chromatography/SEC. Oligomannose-type and hybrid glycans (green) were identified by their sensitivity to Endo H digestion. Pink indicates complex glycans. Rel., relative. (C) Negative ion electrospray spectra of N-glycans found on the gp120 (upper panel) and gp41_ECTO_ (middle panel) subunits of BG505 SOSIP.664 trimers; and mobility-extracted singly charged negative ions from desialylated gp41_ECTO_ glycans (bottom). Symbols are as explained in (B). See also Table S1.

The design and structure of the native-like BG505 SOSIP.664 trimer have been described elsewhere and are summarized in Figure 1A (Binley et al., 2000, Julien et al., 2013b, Khayat et al., 2013, Klasse et al., 2013, Lyumkis et al., 2013, Sanders et al., 2002, Sanders et al., 2013). We first produced BG505 SOSIP.664 trimers from a stable HEK293T cell line as previously described (Chung et al., 2014). We analyzed the enzymatically released and fluorescently labeled N-glycans by hydrophilic interaction chromatography-ultra-performance liquid chromatography (HILIC-UPLC) to determine the trimer’s overall glycan profile (Figure 1B). Endoglycosidase H (Endo H) cleavage of the released glycan pool established that 63% of the total glycans were of the oligomannose type.

To probe the glycan structures in greater detail and to facilitate the processing of subsequent site analysis data, we generated a database of the N-glycans present. For this purpose, unlabeled glycans released from the gp120 and gp41 subunits were analyzed separately by ion mobility-electrospray ionization mass spectrometry (IM-ESI MS; Figure 1C). Glycans were identified, and isomeric structures were assigned using negative ion fragmentation mode (Harvey et al., 2011). In total, 52 and 59 isobaric structures were identified on gp120 and gp41, respectively (Table S1). Consistent with the UPLC data, a significant oligomannose population was present on gp120. We obtained fine structural details of the highly processed gp41 glycans, which were dominated by unsialylated galactose-terminating bi- and triantennary structures (Figure 1C).

Overall, the mass spectrometry (MS) data reveal the extremes of glycan processing and protection that are displayed across the surface of the densely glycosylated trimer. The site-specific variation in glycan structures arises from the intersection between the folding of Env into its quaternary structure and how the producer cell’s processing enzymes then see the folded trimer.

### Quantitative Site-Specific Glycan Analysis

To quantify the distribution of under-processed oligomannose-type glycans and specify the locations of the highly processed complex-type glycans, we used a parallel mass-spectrometric-based approach exploiting two different ionization modes.

In one strategy, we generated glycopeptides from BG505 SOSIP.664 trimers by trypsin digestion, fractionated them by reverse-phase high-performance liquid chromatography (RP-HPLC), and analyzed the resulting glycopeptide pools using MALDI-TOF MS. In total, 11 glycan sites could be isolated and quantified by assessment of the ion abundances (Figure S1; Tables S2 and S3). In an alternative approach, the total pool of trypsin- and chymotrypsin-digested glycopeptides was analyzed by coupled, in-line liquid chromatography-ESI (LC-ESI) MS. Here again, we determined the relative abundance of each specific glycan structure by summing the ion intensity of the corresponding glycopeptide over all identified charge states. This quantification procedure is justified, because protonation of glycopeptides occurs mainly on the peptide backbone; the method has been validated previously, albeit using simpler targets (Wada, 2013). Nevertheless, our complementary analytical approaches enabled an additional validation of the relative quantification performed here.

**Figure 2:**
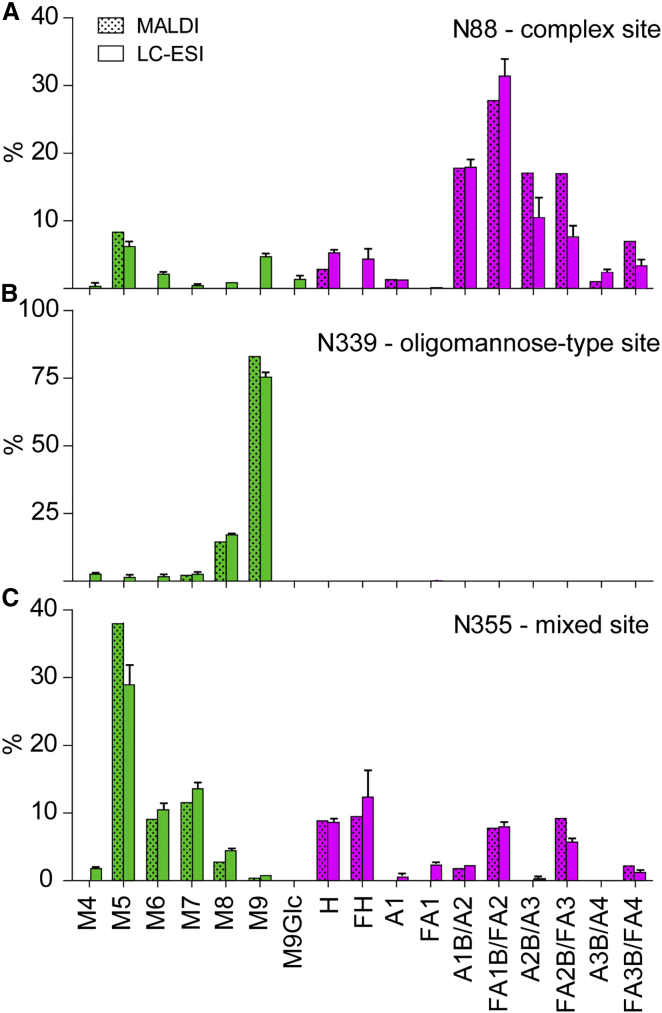
Comparison of Glycan Abundances Determined by MALDI-TOF MS and LC-ESI MS (A–C) Side-by-side bar graph representation of the relative glycan abundances determined by MALDI-TOF MS and LC-ESI MS for three representative sites identified by trypsin digests. Shown are examples of N-glycosylation sites dominated by (A) complex-type glycans (pink; N88), (B) oligomannose glycans (green; N339), or (C) a mixture of structures (N355). Repeats of the LC-MS ESI analysis were performed, and the SEM is indicated on the bar graphs. Figure S1 shows the quantification of the remaining glycosylation sites isolated and analyzed by MALDI-TOF MS. Glycans were grouped as shown in Table S2 and quantified by summing up ion intensities. Briefly, the grouping is based on the number of residues within the oligomannose series (M) or the number of antennae (A) with and without core fucose (F) in a complex glycan. See also Tables S3, S4, and S5.

**Figure 3:**
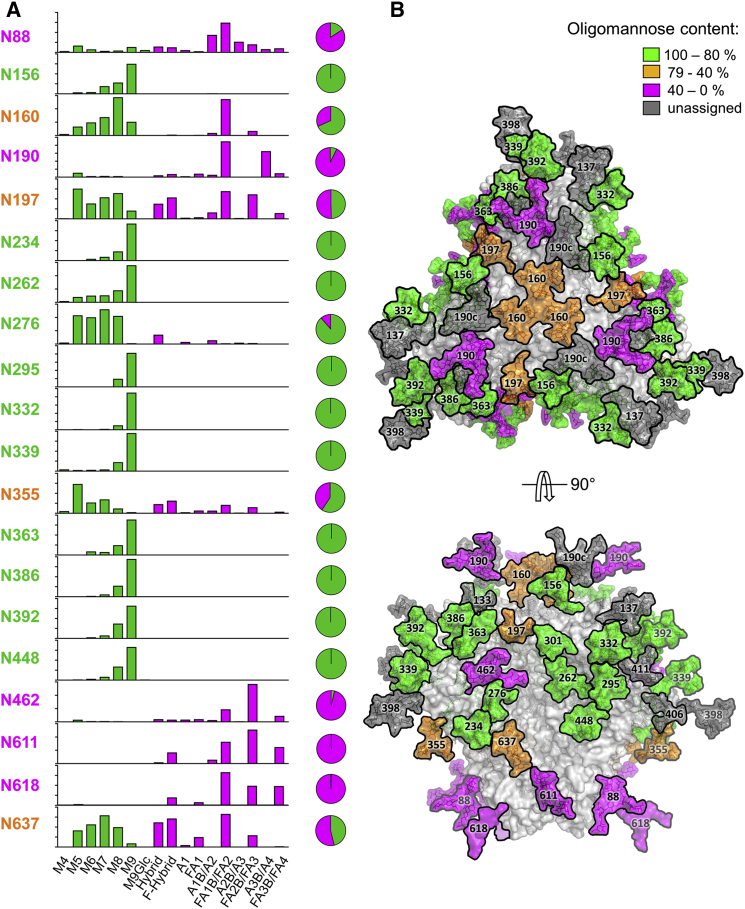
Site-Specific Glycosylation Profiles of BG505 SOSIP.664 Trimers (A) Relative quantification of 20 N-glycosylation sites of BG505 SOSIP.664 trimers, stably produced in HEK293T cells. The trimers were digested with trypsin or chymotrypsin, enriched for glycopeptides, and analyzed by LC-ESI MS. Glycans were grouped as shown in Table S2. The bar graphs represent the means of two analytical replicates; the pie charts summarize the quantification of oligomannose-type (green) and complex/hybrid glycans (pink) on individual sites. Quantifications are based on the peak lists in Tables S4 and S5. Percentages corresponding to this figure can be found in Table S6. (B) Model of a fully glycosylated BG505 SOSIP.664 trimer. The model was constructed using PDB: 5ACO (Lee et al., 2015), with the following glycans modeled according to the processing state: oligomannose-type (Man_9_GlcNAc_2_; Woods et al., 1998), processed (galactosylated biantennary glycan; from PDB: 1L6X), and mixed (Man_5_GlcNAc_2_; Woods et al., 1998) sites. N301 is classified as an oligomannose-type glycan based on the cryo-EM structure (Lee et al., 2015) and previously published MS analysis (Guttman et al., 2014). Deleting the nearby glycan at residue N276 increases both the on- and off-rate constants of the binding of CD4bs-targeting antibodies (Figure S2). See also Figure S2 and Tables S2, S4, and S6.

The glycan compositions derived by the two methods are compared in Figure 2, which shows three representative N-glycan sites corresponding to those dominated by oligomannose structures, complex-type structures, or a mixed population of both glycan classes. The two datasets are highly concordant, indicating that each method reliably captures the processing state of an isolated glycosylation site. Using the glycan database generated by the ion mobility MS of released glycans (Figure 1), analysis of the LC-ESI MS data allowed 20 occupied N-linked glycosylation sites to be characterized in detail (Figure 3; Tables S4, S5, and S6). The remaining eight sites could not be quantified; they were present either on a peptide containing more than one N-glycan (e.g., N133 and N137) or on a glycopeptide that could not be identified with sufficient confidence (e.g., N398 and N625). The latter scenario is likely to be attributable to either incomplete occupancy of the N-glycosylation site or a low ionization efficiency.

The overall percentages of oligomannose-type versus processed glycans confirm that the glycosylation sites on the gp120 subunits are largely dominated by oligomannose-type structures, whereas more highly processed glycans dominate on gp41. However, there are exceptions to these generalizations on both subunits, which we explore later. We generated a map of the distribution of the different processing states across the BG505 SOSIP.664 trimer by constructing a model based on its cryoelectron microscopy (cryo-EM) structure at a 4.36-Å resolution (Lee et al., 2015) (Figure 3B). The model reveals a mosaic of sites bearing oligomannose-type glycans, complex glycans, or a mixture of both (green, pink, and orange, respectively; Figure 3). However, the distribution is not random, and a network of oligomannose glycans is evident across the trimer surface. The heterogeneity of the glycan-processing states indicates that there must be hotspots of accessibility to the producer cell’s glycan-processing enzymes. In the following text, we discuss examples of how even closely proximal glycans can differ substantially in their processing states.

### Clusters of Under-processed Glycans

The trimer apex is covered by a ring of six glycans created by two individual sites (N156 and N160) from the V1/V2 regions of each of the three protomers. All six glycans in this ring are minimally processed, which we attribute to inter-glycan interactions among these topologically proximal structures that shield each of them from the processing enzymes. Man_9_GlcNAc_2_ moieties dominate at the N156 position, whereas the N160 glycan is slightly more processed, although still predominantly of the oligomannose type (Figure 3). Glycans N234 and N276 near the CD4-binding site (CD4bs) also behave as a linked, minimally processed pair on each individual gp120 protomer, and we note that they are separated at the Asn Cα positions by only 10.6 Å (PDB: 5ACO) (Lee et al., 2015). This degree of proximity may again be sufficient for bidirectional protection from enzymatic processing, albeit with one partner again being slightly more shielded than the other. Thus, here, N276 is partially oligomannose-trimmed toward Man_5_GlcNAc_2_, with some hybrid and small complex structures also present, whereas N234 is even less processed. The way in which glycans proximal to the CD4bs are processed has been proposed to influence how antibodies interact with this critical region of Env (Kong et al., 2010). In this general context, it is relevant that Fab fragments of several CD4bs-targeting antibodies bind markedly faster, but also with higher off-rate constants, to BG505 SOSIP.664 trimer mutants lacking the N276 glycan that abuts the CD4bs (Figure S2). A further example of how the dense clustering of glycans may sterically impede the access of glycan-processing enzymes to their substrates involves the mannose patch on the gp120 outer domain (Figure 3B).

### Fine Structure of the Mannose Patch

Two densely packed microclusters are centered on residues N295 and N392 that, together, form a large patch of predominantly Man_9_GlcNAc_2_ glycans (Figure 3B). Here, the N295 glycan is intimately embraced by its counterparts at positions N262, N448, N332, and N301. We were unable to characterize the N301 glycan in this analysis, but oligomannose-type glycans have previously been identified at this position (Guttman et al., 2014). The N392, N363, and N386 glycans form a closely packed microcluster, with their Cα atoms as close together as 7.1 Å (N363 to N392), 8.8 Å (N363 to N386), and 11.5 Å (N386 to N392) (PDB: 5ACO) (Lee et al., 2015). This roughly triangular array of glycans is further surrounded by those on N137, N197, N133, and N339. The N332 glycan, a key component of the so-called “supersite” of immune vulnerability (Kong et al., 2013, Mouquet et al., 2012, Pejchal et al., 2011, Walker et al., 2011), resides at the heart of this site. Its location between the two component microclusters links them into a single large oligomannose patch that stretches around the entire gp120 outer domain. The N332 glycan itself is in close contact with, or in close proximity to, N295, N301, and N137. Overall, the mannose patch is stabilized by the now-revealed glycan-glycan interactions within and/or between the two individual microclusters.

### Interprotomer Control of Glycan Processing

In addition to glycan clustering effects, we also found regions of the trimer where interprotomer interactions and the resulting steric effects are likely to limit glycan processing. For example, N197 bears a mix of oligomannose-type and complex N-glycans and resides very close to the V3 region of gp120 from the adjacent protomer (Figure 3B). As such interactions could only occur in the context of a properly assembled trimer, our observation is not inconsistent with reports that the N197 glycan of monomeric gp120 proteins contains complex glycans (Go et al., 2013, Leonard et al., 1990, Pritchard et al., 2015b). The same general scenario applies to the N156, N160, and N276 glycans that are complex when in the context of monomeric gp120 (Go et al., 2013, Leonard et al., 1990, Zhu et al., 2000). We now show that each of these glycans is predominantly of the oligomannose type when present on the BG505 SOSIP.664 trimer. Hence, all of these sites are subject to the additional constraints imposed on processing enzymes by the interprotomer interactions that apply at the trimer level but that are irrelevant to gp120 monomers (Pritchard et al., 2015d). Overall, our analyses reveal key molecular features of the TAMP (Crispin and Doores, 2015).

### Processing of the Exposed Glycans of the Trimer Base

Complex glycans dominate the N and C termini of the BG505 SOSIP.664 trimer, which are located very close to one another at the structure’s base (Figure 3). In particular, the highly processed gp120 N88 glycan is located immediately proximal to the gp41 subunit. Of the three glycan sites identified on gp41, N611 and N618 display a broad range of complex glycans (Figure 3; Tables S4 and S5), whereas N637 is less extensively processed; a significant proportion of these structures are in oligomannose-type form. An explanation for the mixed-processing status of the N637 glycan could be trimer-induced steric hindrance caused by its proximity to gp120 glycans N234 and N276. The comparatively limited (relative to gp120) overall oligomannose content of the gp41 subunit that was identified by UPLC analysis (Pritchard et al., 2015d) may, therefore, originate from only a single site, N637. We were only able to identify trace amounts of glycosylated peptides corresponding to the N625 site that were insufficient to verify by tandem MS (MS/MS). Hence, the N625 glycan site may be markedly less occupied than the other gp41 sites. There are previous reports of the partial or alternative occupancy of gp41 glycan sites in general (Depetris et al., 2012, Go et al., 2011, Go et al., 2015, Pabst et al., 2012) and of the BG505 SOSIP.664 trimer’s N625 glycan in particular (Guttman et al., 2014).

The BG505 SOSIP.664 trimer used for these glycan site analyses is a soluble protein. It lacks not only the transmembrane region of gp41 but also the membrane-proximal external region (MPER) (Khayat et al., 2013, Klasse et al., 2013). We note that the highly processed N611 and N618 glycans are each likely to be located very close to where the MPER would be located. Thus, in the context of full-length Env, additional shielding effects created by the MPER and the virion or cell membrane may provide additional constraints on processing of the nearby N611 and N618 sites. Even though the overall glycosylation profile of a soluble SOSIP.664 trimer and a sequence-matched, membrane-associated Env ΔCT trimer are broadly similar (Pritchard et al., 2015d), there may be structurally explicable differences in how specific sites are processed in the different contexts.

### The Mannose Patch Is Reshaped by the Presence of a Glycan at N332

**Figure 4:**
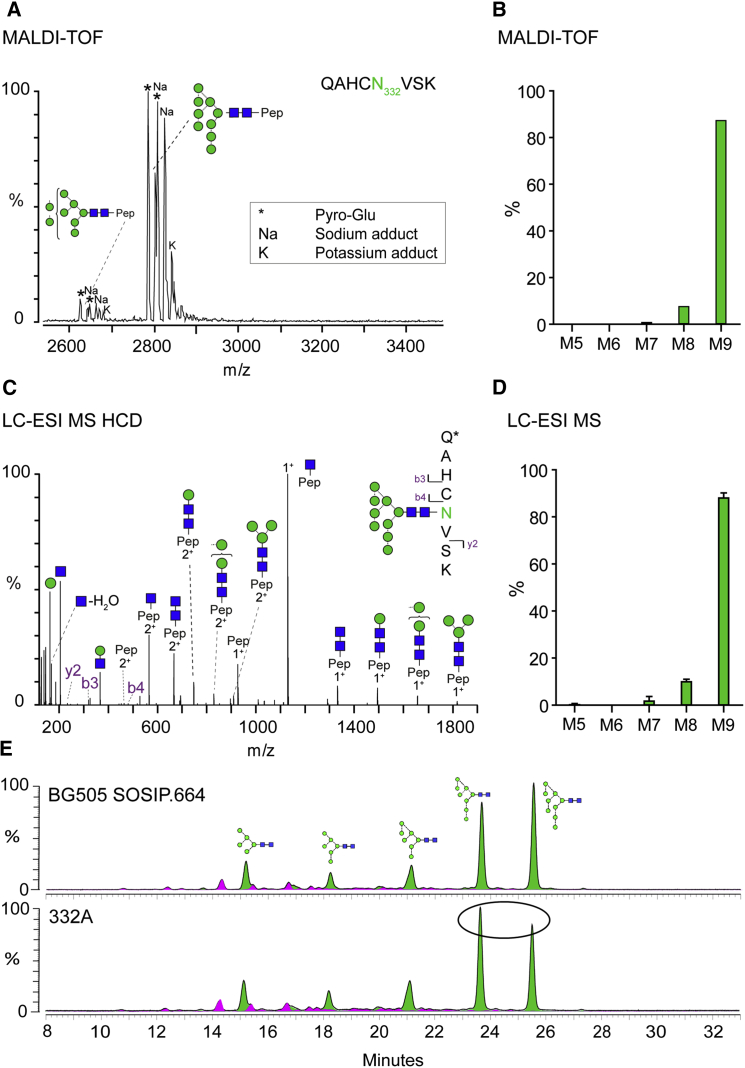
Glycosylation of N332 and Its Effect on the Glycan Shield (A) MALDI-TOF MS spectrum of a tryptic glycopeptide containing N332. Observed modifications are indicated in the box and highlighted in the spectrum. (B) Quantification of peak areas observed in the MALDI-TOF spectrum. (C) LC-ESI MS HCD fragmentation of the same tryptic peptide (Pep) containing N332. (D) Quantification of ion intensities for glycans identified for N332 and analyzed by LC-ESI MS. Error bars represent SEM. (E) HILIC-UPLC profiles of glycans released from BG505 SOSIP.664 transiently produced in HEK293F cells and purified by PGT151-affinity chromatography. Bottom spectrum: alanine-mutated PNGS N332. Green indicates oligomannose-type and hybrid glycans; pink indicates complex glycans. Oval indicates region of spectrum exhibiting changes upon N332A mutation.

The N332 glycan at the heart of the mannose patch on the BG505 SOSIP.664 trimer is clearly dominated by Man_9_GlcNAc_2_ moieties, as shown both by MALDI-TOF MS (Figures 4A and 4B) and LC-ESI MS (Figures 4C and 4D). We note that the transmitted/founder virus isolated from the BG505 infant 6 weeks after birth does not contain an N-glycosylation site at position N332 (Sanders et al., 2013, Wu et al., 2006). The SOSIP.664 trimer is based on a clone from the week-6 isolate, but with the N332 glycan specifically introduced to create epitopes for the multiple bNAbs that recognize it (Kong et al., 2013, Pejchal et al., 2011, Scanlan et al., 2002, Sok et al., 2014, Walker et al., 2011). To assess the impact of the introduced N332 glycan, we expressed and analyzed BG505 SOSIP.664 (i.e., N332 present) trimers and its N332A mutant in HEK293F cells and determined their glycan profiles. Comparing the resulting two HILIC-UPLC profiles shows that the knocked-in N332 glycan reshapes the overall glycan profile. Thus, compared to the standard trimer, the glycans released from the N332A mutant contain fewer Man_9_GlcNAc_2_ moieties, but more Man_8_GlcNAc_2_, so that the latter was now the most prominent N-glycan structure (Figure 4E). We interpret this observation to mean that, when the N332 glycan is present, it influences the composition of the multiple, closely proximal glycans within the mannose patch. A likely mechanism for this effect is steric obstruction of α-mannosidase enzymes (Pritchard et al., 2015b). More generally, we propose that, when the glycan shield evolves under immune selection pressure during HIV-1 infection, there may be subtle influences on the glycan composition of sites located some distance away from the point at which a glycan is added or deleted.

### Effect of Glycan Site Deletions on Neutralization Sensitivity and Structure Integrity

**Figure 5:**
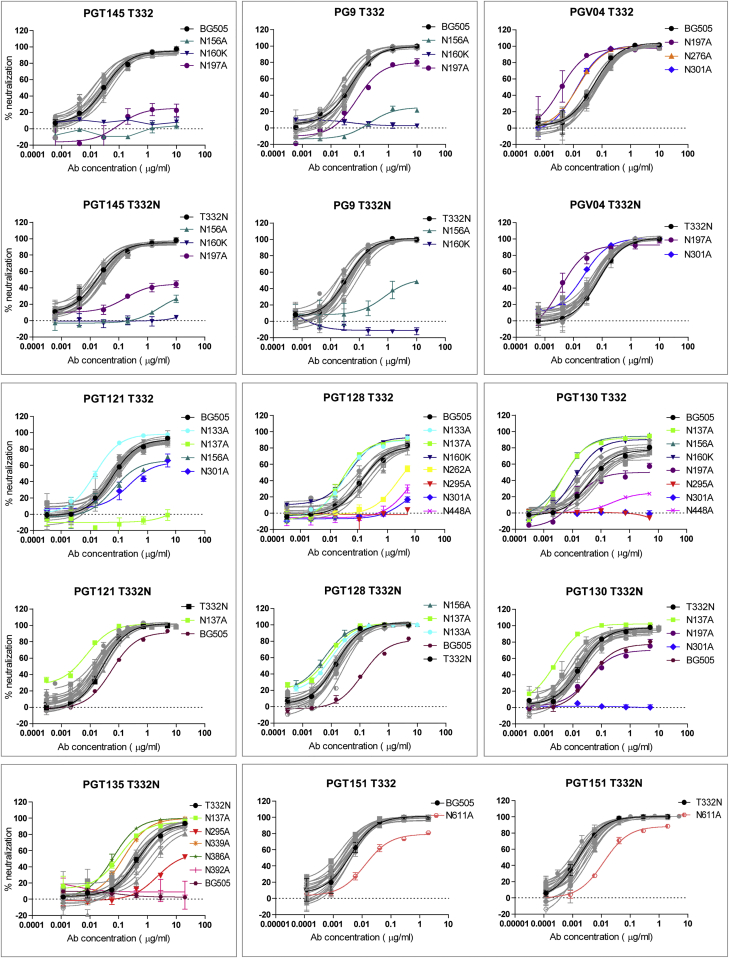
Glycan Site Modulation of Viral Neutralization by Glycan-Dependent bNAbs Neutralization of wild-type BG505 (termed BG505.T332 in the main text) and BG505.T332N Env-pseudoviruses, and a panel of glycan-deletion mutant Env-pseudoviruses, by bNAbs PGT145, PG9, PGV04, PGT121, PGT128, PGT130, PGT135, and PGT151. The presence or absence of the N332 glycan is indicated above each panel (i.e., T332 or T332N). The plots show data from one experiment that is representative of at least two. The error bars are based on the mean values derived from two duplicate wells ± SEM. The glycan-deletion mutants based on the BG505.T332 virus were not analyzed for their sensitivity to PGT135, as this antibody (Ab) is highly N332 dependent, and the parental virus already lacks this glycan (Kong et al., 2013). Mutant Env-pseudoviruses with markedly increased or decreased neutralization sensitivity compared to the remaining panel are highlighted using the colors shown in the legends. The full list of PNGS deletions is in Table S8.

To determine the impact of glycan deletions on bNAb sensitivity, we removed various PNGSs from the BG505.T332 (i.e., wild-type BG505) and the BG505.T332N Env-pseudotyped viruses (note that the BG505.T332N virus has the same glycosylation sites as BG505 SOSIP.664 trimers) (Sanders et al., 2013, Sanders et al., 2015). Then, we determined neutralization profiles for a range of bNAbs to multiple epitopes: CD4bs (PGV04), mannose-patch binding (PGT121, PGT128, PGT130, and PGT135), V1/V2 loop (PG9 and PGT145), and gp120/gp41 interface (PGT151) (Figure 5). As expected, when glycans known to be key components of epitopes were absent, bNAb sensitivity was substantially reduced or lost entirely. For example, removing the N156 or N160 glycans from either of the BG505 test viruses reduced the neutralization activities of PG9 and PGT145; likewise, for glycans N295 or N301 with PGT128 and PGT130. However, we also observed some unexpected outcomes. Thus, eliminating the N197 glycan substantially reduced (BG505.T332) or entirely ablated (BG505.T332N) the neutralization activity of the V1/V2-binding bNAb PGT145. Similarly, deleting the N197 glycan from the BG505.T332 virus also reduced its sensitivity to PG9. We note that the N197 site contains a mixture of glycoforms (Figure 3) and is located at the trimer apex near the protomer interface. We propose that its removal indirectly affects the binding of various mannose-dependent bNAbs by perturbing the glycans at the trimer apex.

**Figure 6:**
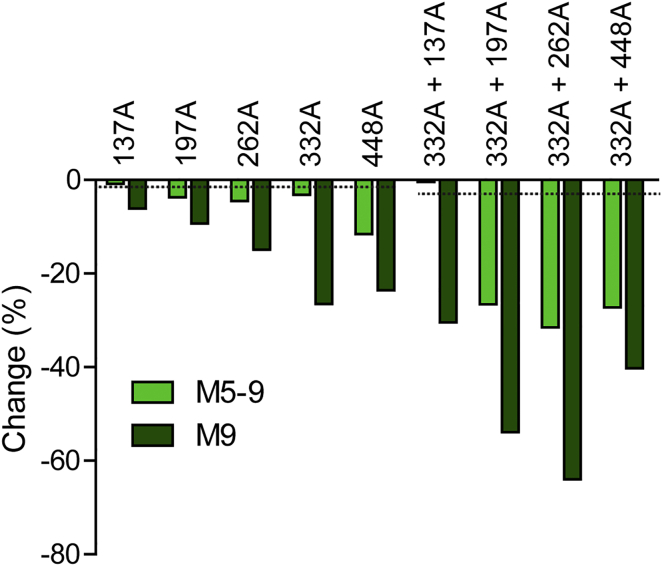
Impact of Individual Glycan Sites on Total Trimer Glycosylation The figure shows the effects of various single- and double-glycan site deletions on the overall abundance of oligomannose-type glycans (M5-9; green), as well as on the individual abundance of Man_9_GlcNAc_2_ (M9; dark green). His-tagged BG505 SOSIP.664 trimers were transiently expressed in HEK293F cells and purified by PGT151-affinity chromatography. Bands corresponding to the trimeric protein were excised from native gels and subjected to glycan analysis. The abundance of oligomannose-type glycans were determined by the integration of corresponding HILIC-UPLC peaks with or without Endo H treatment. The y axis represents the change in percentage of oligomannose-type glycans of a mutant relative to the non-mutated BG505 SOSIP.664 trimer control and is calculated as follows: percent change = [(% oligomannose in control − % oligomannose in mutant)/(% oligomannose in control)] × 100. The two dashed lines represent the decrease in oligomannose abundance that would be predicted to arise solely by the deletion of one or two N-glycosylation sites that are exclusively occupied by oligomannose-type glycans when present on the control trimer. See also Figure S3.

To further explore some of these phenomena, we created several double-PNGS deletion mutant trimers on the BG505 SOSIP.664 N332A background, together with the corresponding single mutants. The trimers were produced as described earlier, and their glycan profiles were analyzed by HILIC-UPLC. The relative impact of single-site deletions on the abundance of oligomannose-type glycans was generally significantly less than when two sites were deleted (Figure 6; Figure S3). However, it is notable that the overall reduction in oligomannose content transcends what would be predicted solely from the engineered loss of the individual glycans. As noted earlier, the N332 site has a significant influence on the wider composition of the glycan shield on BG505 SOSIP.664 trimers. This observation is reinforced by studies using mannose-patch binding bNAbs and mutant viruses (Figure 5). Specifically, the BG505.T332 virus (which lacks the N332 glycan) is more affected by the deletion of additional glycans, compared to BG505.T332N (which contains it). Thus, unexpectedly, the glycan epitopes for PGT128 and PGT130 are particularly strongly impaired when the N448 glycan is deleted from the BG505.T332 virus, more so than seen with BG505.T332N. Of note is that, in the SOSIP.664 trimer context, the double deletion of N332 and N448 has quite a strong impact on the overall abundance of oligomannose, as does the dual elimination of N332 and N262 (Figure 6). We noted that deleting the N262 glycan also reduces the sensitivity of the BG505.T332 virus to neutralization by PGT128. However, the impact of removing the N262 glycan may be explained by its critical role in gp120 folding (Mathys et al., 2014, Moore et al., 1994) and in influencing the conformation of the N301 glycan (Kong et al., 2015).

Conversely, and unexpectedly, certain bNAbs to the mannose-patch epitopes more potently neutralize BG505 virus mutants that lack some outer domain glycans. Deleting the N137 glycan renders both BG505 test viruses more sensitive to PGT128 and PGT130 and makes BG505.T332N more vulnerable to PGT121 and PGT135, but the corresponding change has no meaningful effect on oligomannose content in the SOSIP.664 trimer context (Figure 6). The likely explanation is that the loss of the N137 glycan increases the overall accessibility of the mannose-patch epitopes but without affecting the average composition of the glycan shield (Doores et al., 2015, Garces et al., 2015).

The CD4bs antibody PGV04 more strongly neutralized the N197 and N301 single-deletion mutants of the BG505.T332 and BG505.T332N viruses. This outcome is consistent with, and extends, a recent report that deleting the N197 glycan from a diverse panel of HIV-1 isolates generally increases their sensitivity to CD4bs bNAbs, presumably via increased epitope accessibility (Townsley et al., 2015).

### Glycan-Dependent Epitope of the Trimer Apex

The PG9 epitope is critically dependent on the trimer apex glycans N156 and N160 (Amin et al., 2013, Julien et al., 2013a, Walker et al., 2009). On the BG505 SOSIP.664 trimer, the N156 site is exclusively occupied by oligomannose-type glycans, mainly Man_9_GlcNAc_2_ (Figure 3). This observation is in marked contrast with a report that sialylated complex structures play a critical role in how PG9 recognizes the N156 glycan (Amin et al., 2013) and also with the proposal that sialylated hybrid glycans are targeted by the related PG16 antibody (Pancera et al., 2013). These studies used a scaffolded V1/V2 region of the trimer, and we suggest that, although antibodies may be able to bind such fragments, their glycans may not be close mimics of the native setting. A related observation is that, in the trimer context, N160 can now be seen to be occupied by a mixed array of glycans, in which Man_8_GlcNAc_2_ predominates. This information supersedes the suggestion, based on simpler structures, that processed Man_5_GlcNAc_2_ moieties play a critical role in how PG9 recognizes the N160 glycan (Amin et al., 2013). It is unclear how the presence of kifunensine-induced Man_9_GlcNAc_2_ moieties impedes viral neutralization by PG9 and PG16 (Doores and Burton, 2010). One explanation is that PG9 and PG16 may simply not bind Man_9_GlcNAc_2_ structures very well. However, an alternative hypothesis is that driving all three N160 glycans to the larger structure (i.e., by the use of kifunensine) distorts the packing of the trimer apex, with implications for the presentation of the PG9 epitope. Overall, we now show that the PG9 epitope, in the trimer context, is substantially more oligomannose tolerant/dependent than has been thought. Our findings are concordant with the observation that PG9 binds comparably to BG505 SOSIP.664 trimers regardless of whether they are produced in HEK293T cells (which permit glycan processing) or GnTI-deficient HEK293S cells (which restrict glycans to oligomannose forms) (Julien et al., 2013a).

The knowledge we present on the site-specific glycan composition of bNAb epitopes should inform us about the types of immunological response that Env vaccine candidates may need to induce. As additional native-like recombinant trimers become available, including ones specifically engineered to better present epitopes for glycan-influenced bNAbs or their germline precursors, site-specific glycan analysis will become a critical tool that will, in turn, generate yet more information to guide further design improvements. In the context of HIV-1 infection, we also propose that the dense network of glycans that covers the Env trimer will tend to promote the presence of particular carbohydrate structures, despite the underlying and much greater diversity at the amino-acid-sequence level.

## Experimental Procedures

A more detailed description of the experimental procedures is provided in the Supplemental Experimental Procedures.

### Overview of BG505 Constructs

BG505 SOSIP.664 trimers used for site-specific MS analysis were stably expressed in HEK293T cells as previously described (Chung et al., 2014). The effects of deleting individual glycan sites were studied by making specific mutants of His-tagged BG505 SOSIP.664 trimers (Sanders et al., 2013), which were transiently expressed in HEK293F cells. Neutralization assays were conducted with Env-pseudoviruses produced in HEK293T cells. Viruses based on the full-length wild-type BG505 Env (termed here BG505.T332), or the BG505.T332N point mutant in which the N332 glycan site was restored, served as the basis for introducing specific mutations to delete one or two PNGSs. Mutations were made using the QuikChange Mutagenesis (Agilent Technologies) system according to the manufacturer’s instructions. The primers used and the mutants created are listed in Tables S7 and S8, respectively.

### Expression and Purification of Env Trimers

BG505 SOSIP.664 trimers used for MS analysis of site-specific N-glycosylation were expressed in stable Flp-In HEK293T cells and purified by 2G12-affinity chromatography followed by size exclusion chromatography (SEC), as previously described (Chung et al., 2014). His-tagged BG505 SOSIP.664 trimers and corresponding PNGS mutants (discussed earlier) were transiently expressed in HEK293F cells, as described elsewhere (Sanders et al., 2013). The trimers were then purified from cell culture supernatants by PGT151-affinity chromatography, as described elsewhere (Pritchard et al., 2015d, Ringe et al., 2015).

### Env-Pseudovirus Production and Neutralization Assays

To produce Env-pseudoviruses capable of single-cycle replication, HEK293T cells were cotransfected with plasmids encoding Env and an Env-deficient genomic backbone. Supernatants containing Env-pseudoviruses were harvested 72 hr post-transfection for use in neutralization assays based on TZM-bl target cells (Montefiori, 2005).

### In-Gel Release of N-Linked Glycans

N-linked glycans were enzymatically released from trimers via in-gel digestion with PNGase F. The released glycans were then either fluorescently labeled or subjected to IM-ESI MS.

### Glycan Analysis by HILIC-UPLC

Released glycans were fluorescently labeled with 2-aminobenzoic acid (2-AA), as previously described (Neville et al., 2009, Pritchard et al., 2015d). Labeled glycans were analyzed using a 2.1 mm × 10 mm Acquity BEH glycan column in a Waters Acquity UPLC instrument. Endo H digestions of labeled glycans were used to measure the abundance of oligomannose-type glycans.

### Tandem IM-ESI MS Analysis of Released N-Linked Glycans

The total pool of released glycans from BG505 SOSIP.664 trimers (from the stable HEK293T cell line) was analyzed using IM-ESI MS with a Waters Synapt G2Si mass spectrometer (Waters). These results were the basis for the creation of a glycan library that was then used for the subsequent site-specific N-glycosylation analyses.

### Proteolytic Digestion of BG505 SOSIP.664 Trimers and Glycopeptide Enrichment

A 200- to 300-μg sample of trimer was used for in-solution proteolytic digestion using trypsin or chymotrypsin (Mass Spectrometry Grade, Promega), followed by enrichment of the digestion mixture for glycopeptides using the ProteoExtract Glycopeptide Enrichment Kit (Merck Millipore). Glycopeptides were then either directly analyzed by LC-ESI MS or fractionated by RP-HPLC and subjected to MALDI-TOF MS.

### RP-HPLC Fractionation and MALDI-TOF MS Analysis of Glycopeptides

Glycopeptides were resuspended in PBS buffer and fractionated with RP-HPLC a Jupiter C18 5-μm 250 × 4.5 mm column (300 Å, Phenomenex) and a Dionex U3000 LC system. Glycopeptide fractions were split in half. One half was directly analyzed by MALDI-TOF MS, whereas the other half was deglycosylated using PNGase F (New England Biolabs). MALDI-TOF MS was performed using an Autoflex Speed MALDI-TOF(/TOF) instrument (Bruker). MS/MS was performed on both glycopeptides and peptides to confirm peptide identity. Acquired data were processed using DataAnalysis 3 software (Bruker).

### LC-ESI MS and MS/MS Analysis of Glycopeptides

Enriched glycopeptides were analyzed on a Q-Exactive Orbitrap mass spectrometer (Thermo Fisher Scientific) coupled to a Dionex Ultimate 3000 nanoLC system. MS data were acquired with XCalibur 3.0.63 (Thermo Fisher Scientific) using Top10 intense ions in 1.42-s duty cycle time. Glycopeptides were fragmented using higher energy collisional dissociation (HCD) fragmentation. Data analysis and glycopeptide identification were performed using Byonic (Version 2.7) and Byologic software (Version 2.3; Protein Metrics).

## Author Contributions

A.-J.B., S.V., L.K.P., D.J.H., R.S.A., S.A.K., A.C., A.K., J.H.L., P.J.K., and G.E.S. performed experimental work. A.-J.B., A.B.W., L.K.P., R.W.S., D.J.H., W.B.S., H.B.K., D.I.R.S., L.R., K.J.D., D.R.B., N.Z, J.H.L., P.J.K., and M.C. analyzed data. A.-J.B., I.A.W., A.B.W., R.W.S., W.B.S., J.P.M., K.J.D., and M.C. wrote the paper. D.R.B., I.A.W., J.P.M., K.J.D., and M.C. designed the study. All authors read and approved the final manuscript.

## Supplemental Information

Document S1. Supplemental Experimental Procedures, Figures S1–S3, and Tables S2 and S6–S8

Table S1. Library of Glycan Structures Identified on BG505 SOSIP.664 Trimers, Related to Figure 1 Structures are represented as shown in Figure 1 using the Oxford glycan nomenclature (Oxford) as previously described (Harvey et al., 2011) and also the nomenclature from the Consortium of Functional Glycomics (CFG). MW: Molecular weight; Calc: Calculated.

Table S3. N-Linked Glycopeptide Compositions of Trypsin-Digested BG505 SOSIP.664 Trimers Identified by MALDI-TOF MS, Related to Figure 2 Site: N-glycosylation site; Mod.: Modifications; Ad.: Adduct; C: Carbamidomethylated; PG: Pyro-Glu; O: Oxidation; Exp.: Experimental determined *m/z*; Calc.: Calculated *m/z*; Intensity: Intensity counts of all isotopic peaks. The second dataset shows N-linked glycopeptide compositions identified from pooled RP-HPLC fractions by MALDI-TOF MS.

Table S4. N-Linked Glycopeptide Compositions of Trypsin-Digested BG505 SOSIP.664 Trimers Identified by LC-ESI MS, Related to Figure 3 Site: N-glycosylation site; XIC: Extracted ion chromatogram; Exp.: Experimental determined mass (shown as a range when different charge states and/or different scans were recorded); Calc.: Calculated mass. All cysteines are carbamidomethylated. Lower case letters in sequence indicate the position of the modification. Table contains data from two analytical replicates.

Table S5. N-Linked Glycopeptide Compositions of Chymotrypsin-Digested BG505 SOSIP.664 Trimers Identified by LC-ESI MS, Related to Figure 3 Site: N-glycosylation site; XIC: Extracted ion chromatogram; Exp.: Experimental determined mass (shown as a range when different charge states and/or different scans were recorded); Calc.: Calculated mass; O: Oxidation. All cysteines are carbamidomethylated. Lower case letters in sequence indicate the position of the modification. Table contains data from two analytical replicates.

Document S2. Article plus Supplemental Information
