# Supplementary material for: Composition and Antigenic Effects of Individual Glycan Sites of a Trimeric HIV-1 Envelope Glycoprotein
Source: Cell Rep. 2016 Mar 10;14(11):2695–706. doi: 10.1016/j.celrep.2016.02.058 (PMC4805854; doi:10.1016/j.celrep.2016.02.058)
Supplement: Document S1. Supplemental Experimental Procedures, Figures S1–S3, and Tables S2 and S6–S8 [file mmc1.pdf]

Cell Reports, Volume *14*

Supplemental Information

Composition and Antigenic Effects of Individual Glycan Sites of a Trimeric HIV-1 Envelope Glycoprotein

Anna-Janina Behrens, Snezana Vasiljevic, Laura K. Pritchard, David J. Harvey, Rajinder S. Andev, Stefanie A. Krumm, Weston B. Struwe, Albert Cupo, Abhinav Kumar, Nicole Zitzmann, Gemma E. Seabright, Holger B. Kramer, Daniel I.R. Spencer, Louise Royle, Jeong Hyun Lee, Per J. Klasse, Dennis R. Burton, Ian A. Wilson, Andrew B. Ward, Rogier W. Sanders, John P. Moore, Katie J. Doores, and Max Crispin

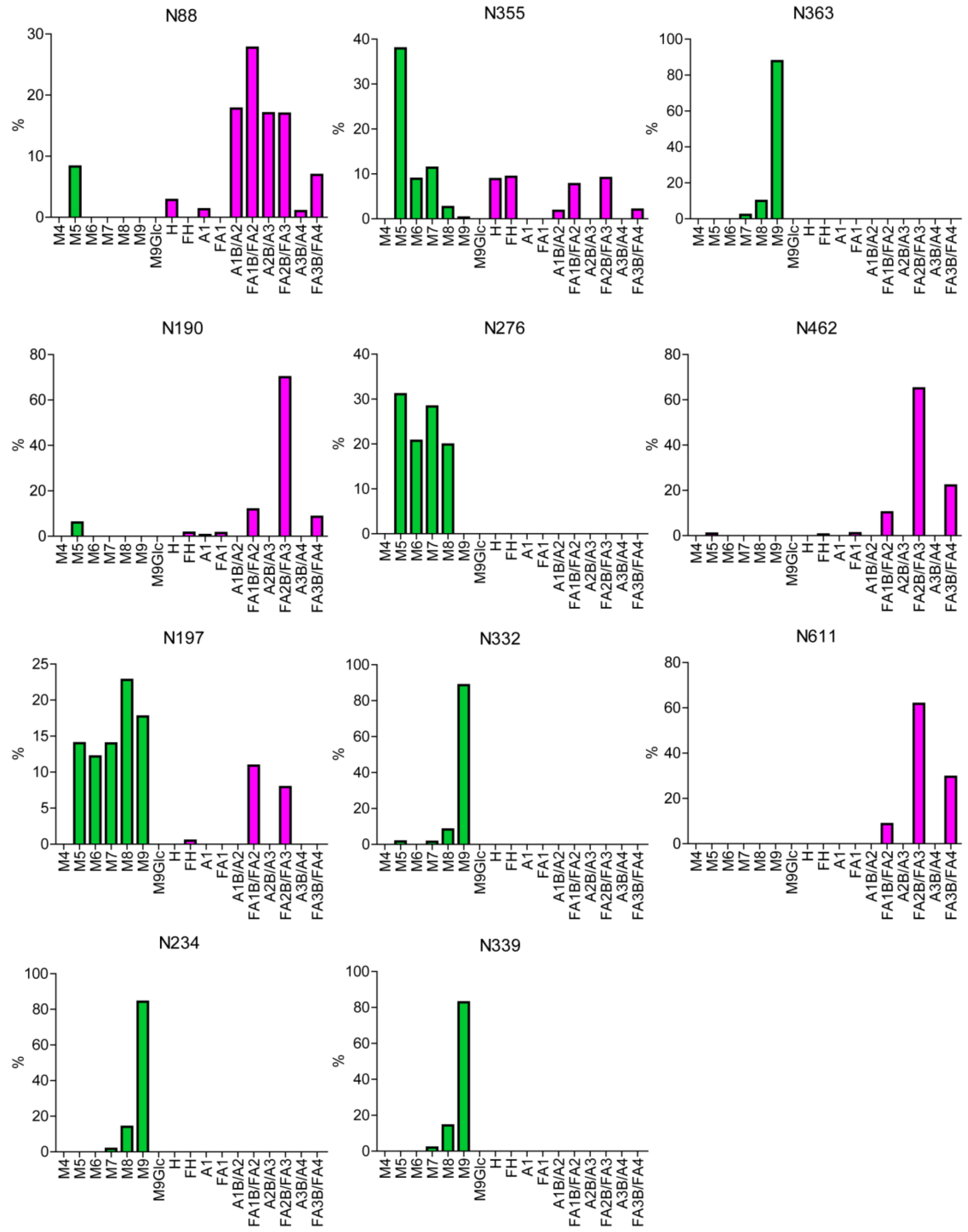

Figure S1. Relative quantification of N-glycosylation sites on BG505 SOSIP.664 trimers identified by MALDI-TOF MS, related to Figure 2. BG505 SOSIP.664 trimers, stably produced in 293T cells and purified by 2G12 affinity chromatography/SEC, were reduced, alkylated, digested with trypsin and then fractionated by RP-HPLC. Fractions were analysed by MALDI-TOF MS and MS/MS in positive mode. In cases where glycopeptides eluted over more than one fraction, the relevant fractions were pooled and reanalysed. The microheterogeneity at individual N-glycosylation sites was quantified in the following way: [Sum of areas of isotopic peaks of one glycoform] / [sum of areas of isotopic peaks of all glycoforms] x 100. Glycans were grouped as shown in Table S2; green: oligomannose-type glycans; pink: hybrid and complex glycans. The peak lists corresponding to these data including intensities can be found in Table S3 and Table S4.

A

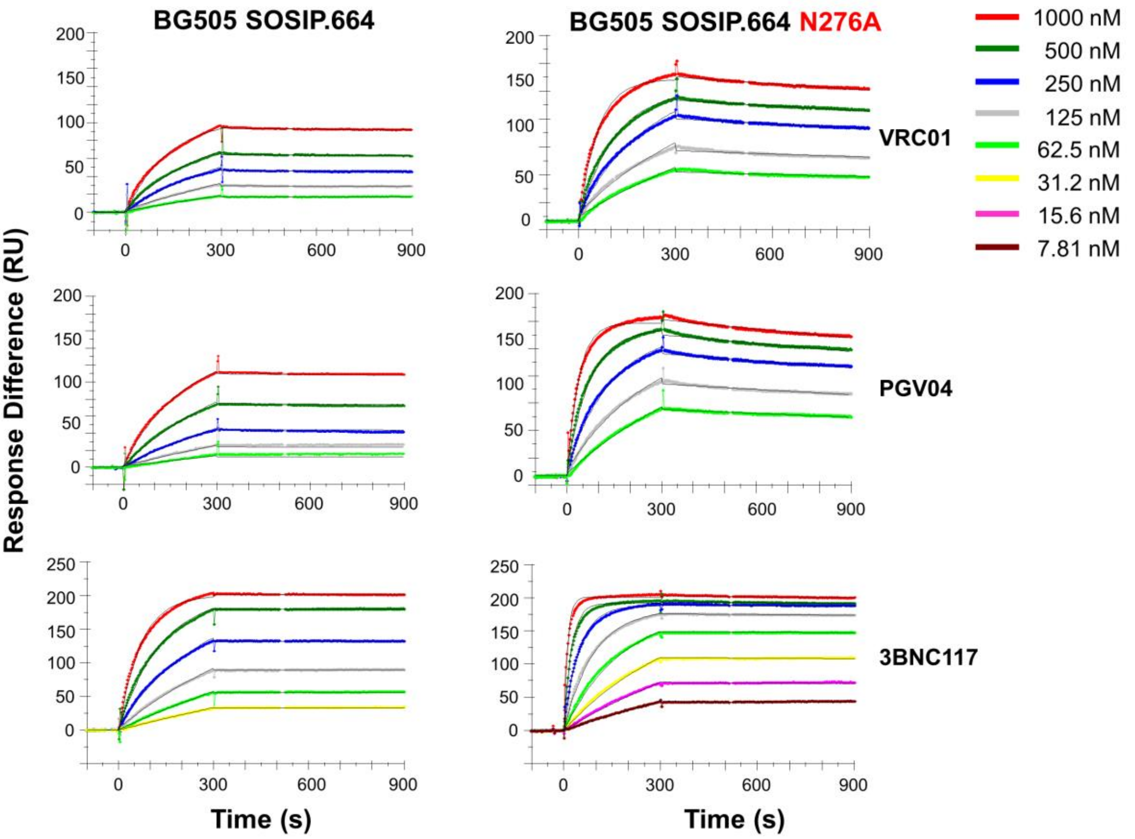

B

mAb (Fab)	BG505 SOSIP.664	k_{on} (1/Ms)	k_{off} (1/s)	K_d (nM)	S_m
**VRC01**	WT (n=2)[a]	$8.6 \cdot 10^3 \pm 1.0 \cdot 10^2$	$< 10^{-5}$ [b]	< 1 [b]	1.6 ± 0.061
	N276A (n=3)	$1.7 \cdot 10^4 \pm 3.3 \cdot 10^2$	$1.7 \cdot 10^{-4} \pm 1.0 \cdot 10^{-5}$	10 ± 0.50	2.5 ± 0.055
**PGV04**	WT (n=2)	$6.7 \cdot 10^3 \pm 50$	$3.2 \cdot 10^{-5} \pm 1.5 \cdot 10^{-6}$	4.8 ± 0.20	2.0 ± 0.034
	N276A (n=3)	$2.6 \cdot 10^4 \pm 3.3 \cdot 10^2$	$2.1 \cdot 10^{-4} \pm 8.8 \cdot 10^{-6}$	8.0 ± 0.40	2.7 ± 0.054
**3BNC117**	WT (n=3)	$1.5 \cdot 10^4 \pm 0$	$< 10^{-5}$ [b]	< 0.1 [b]	3.0 ± 0.053
	N276A (n=3)	$8.4 \cdot 10^4 \pm 1.5 \cdot 10^3$	$2.7 \cdot 10^{-5} \pm 2.9 \cdot 10^{-6}$	0.30 ± 0.040	2.9 ± 0.028

Figure S2. Binding of CD4bs-specific Fabs to BG505 SOSIP.664 and N276A mutant trimers analysed by SPR, related to Figure 3. (A) The binding of Fabs at different concentrations (legend to the right), after background subtraction, is given on the y axes (response units, RU) over the time periods shown on the x axis (s). Association was monitored for 300 s and dissociation for 600 s. Deleting the N276 glycan site increased both the on- and off-rate constants for all three Fabs. (B) Langmuir-modelled binding parameters. [a]Tabulated parameter values were obtained by fitting a Langmuir model to the binding data; the values are the means ± SEM of n independent experiments. [b]Off-rate constants below significant detectability (T<10) and the corresponding dissociation constants are given as <.

A

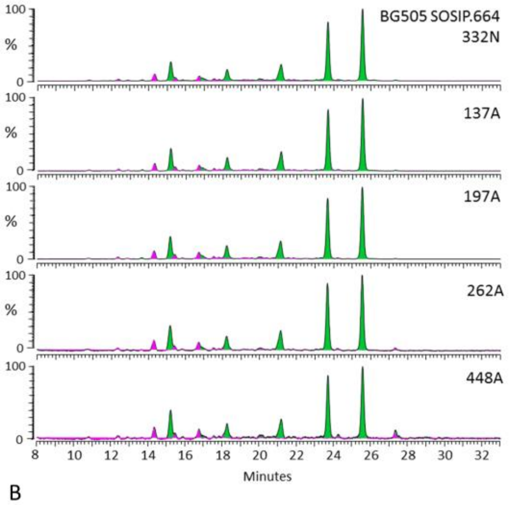

B

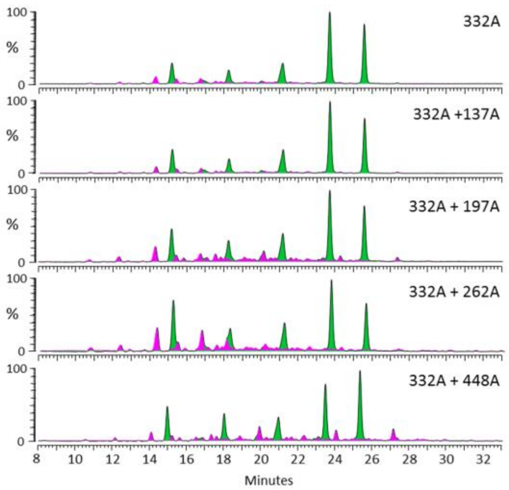

C

	Man5	Man6	Man7	Man8	Man9	sum
BG505 SOSIP.664	8	5	9	24	30	75
137A	9	5	9	24	28	75
332A	8	5	10	27	22	73
197A	9	5	8	23	27	73
262A	10	6	8	23	25	72
332A	8	5	10	27	22	73
448A	10	6	8	20	23	67
332A+137A	9	6	12	27	21	75
332A+197A	9	6	9	18	14	55
332A+262A	12	5	8	16	11	52
332A+448A	9	7	8	14	18	55

Figure S3. HILIC-UPLC profiles of a panel of PNGS-deletion mutants of BG505 SOSIP.664 trimers, related to Figure 6. A selection of single and double N-glycan site knockout mutants of the His-tagged BG505 SOSIP.664 trimer, together with the unmodified control trimer, were expressed by transient transfection of HEK 293F cells and purified by PGT151 affinity chromatography. The eluted Env proteins were resolved by BN-PAGE and the bands corresponding to trimers were excised. N-glycans were released from the gel bands using PNGase F and fluorescently labelled. Glycan profiles with and without Endo H digestion were analysed by HILIC-UPLC. Peaks corresponding to oligomannose-type and hybrid-type glycans are depicted in green, complex-type glycans in pink. (A) The panel of single PNGS knockout mutants. (B) The corresponding panel of N332A double PNGS knockout mutants. (C) Abundances (as percentages of total glycans) of oligomannose-type glycans $Man_{5\text{-}9}GlcNAc_2$ (Man5-Man9). The changes seen in the various mutants, relative to the BG505 SOSIP.664 control trimers, are presented in Figure 6.

Table S1. Library of glycan structures identified on BG505 SOSIP.664 trimers, related to Figure 1. Structures are represented as shown in Figure 1 using the Oxford glycan nomenclature (Oxford) as previously described (Harvey et al., 2011) and also the nomenclature from the Consortium of Functional Glycomics (CFG). MW: Molecular weight; Calc: Calculated.

Table S2. Classification of identified N-linked glycan compositions, related to Figure 2. Glycan names are constructed as follows: Mn = number (n) of mannose residues; An = number (n) of antennae (e.g. A3 = triantennary); Gn = number (n) of galactose residues; F in the front of the name indicates the presence of a core fucose.

Group Name	Members[a]
M4	M4
M5	M5
M6	M6
M7	M7
M8	M8
M9	M9
M9Glc	M9Glc
H/Hybrid	M4A1G1, M5A1G1, M6A1G1, M4A1G1S1, M5A1G1S1,
FH/Fucosylated-Hybrid	FM4A1G1, FM5A1G1, FM4A1G1S1, FM5A1G1S1
A1	A1, A1G1, A1G1S1
FA1	FA1, FA1G1, FA1G1S1
A1B/A2	A2, A2G1, A2G2, A2G1S1, A2G2S2
FA1B/FA2	FA2, FA2G1, FA2G2, FA2G1S1, FA2G2S1, FA2G2S2
A2B/A3	A2B, A2BG1, A2BG2, A3G3, A3G2S1, A3G3S1, A3G3S2, A3G3S3
FA2B/FA3	FA2B, FA2BG1, FA2BG2, FA3G3, FA3G3S1, FA3G3S2, FA3G3S3, FA2BG2S1
A3B/A4	A4, A4G1, A4G2, A4G3, A4G4, A4G4S1, A4G4S2, A4G4S3, A4G4S4
FA3B/FA4	FA3B, FA4G1, FA3BG1, FA3BG2, FA3BG3, FA4G4, FA4G4S1, FA4G4S2, FA4G4S3, FA4G4S4

[a]Isobaric glycans are not listed. Unusual glycan structures with terminal fucoses, sulfates or *N*-acetylgalactosamines are categorized according to the number of their antennae.

Table S3. N-linked glycopeptide compositions of trypsin-digested BG505 SOSIP.664 trimers identified by MALDI-TOF MS, related to Figure 2. Site: N-glycosylation site; Mod.: Modifications; Ad.: Adduct; C: Carbamidomethylated; PG: Pyro-Glu; O: Oxidation; Exp.: Experimental determined *m/z*; Calc.: Calculated *m/z*; Intensity: Intensity counts of all isotopic peaks. The second dataset shows N-linked glycopeptide compositions identified from pooled RP-HPLC fractions by MALDI-TOF MS.

Table S4. N-linked glycopeptide compositions of trypsin-digested BG505 SOSIP.664 trimers identified by LC-ESI MS, related to Figure 3. Site: N-glycosylation site; XIC: Extracted ion chromatogram; Exp.: Experimental determined mass (shown as a range when different charge states and/or different scans were recorded); Calc.: Calculated mass. All cysteines are carbamidomethylated. Lower case letters in sequence indicate the position of the modification. Table contains data from two analytical replicates.

Table S5. N-linked glycopeptide compositions of chymotrypsin-digested BG505 SOSIP.664 trimers identified by LC-ESI MS, related to Figure 3. Site: N-glycosylation site; XIC: Extracted ion chromatogram; Exp.: Experimental determined mass (shown as a range when different charge states and/or different scans were recorded); Calc.: Calculated mass; O: Oxidation. All cysteines are carbamidomethylated. Lower case letters in sequence indicate the position of the modification. Table contains data from two analytical replicates.

Table S6. Semi-quantification of glycopeptides identified by LC-ESI MS, related to Figure 3. The numbers are the % of the total glycan pool at any given position. Glycopeptides are grouped as shown in Table S2. T (trypsin) and C (chymotrypsin) indicate the digestion origins of the glycopeptide used for quantification. Average shows the average abundance of the different glycopeptide groups among the 20 identified N-glycosylation sites.

	N88	N156	N160	N190	N197	N234	N262	N276	N295	N332	N339
**M4**	0.3	0.0	0.7	0.0	0.1	0.0	1.2	0.8	0.0	0.0	2.5
**M5**	6.2	2.1	6.3	4.8	14.9	0.0	7.3	21.3	0.0	0.4	1.3
**M6**	2.1	2.4	9.4	0.7	7.5	2.6	9.4	19.8	0.0	0.0	1.5
**M7**	0.4	14.3	13.4	0.9	10.6	6.6	9.8	25.8	0.0	0.9	2.4
**M8**	0.9	21.7	28.4	0.8	12.7	17.5	17.3	20.8	18.6	9.6	17.0
**M9**	4.7	59.3	9.9	0.0	3.9	73.3	55.0	0.3	81.4	89.1	75.3
**M9Glc**	1.4	0.0	0.0	0.0	0.0	0.0	0.0	0.0	0.0	0.0	0.0
**H**	5.3	0.1	0.0	1.8	7.4	0.0	0.0	6.7	0.0	0.0	0.0
**FH**	4.3	0.0	0.3	4.9	10.5	0.0	0.0	0.0	0.0	0.0	0.0
**A1**	1.3	0.0	0.0	0.6	0.3	0.0	0.0	0.9	0.0	0.0	0.0
**FA1**	0.1	0.0	0.1	3.7	0.6	0.0	0.0	0.0	0.0	0.0	0.0
**A1B/A2**	17.9	0.1	1.5	2.4	3.0	0.0	0.0	2.5	0.0	0.0	0.0
**FA1B/FA2**	31.4	0.0	27.0	43.5	13.7	0.0	0.0	0.2	0.0	0.0	0.0
**A2B/A3**	10.5	0.0	0.0	0.0	0.2	0.0	0.0	0.7	0.0	0.0	0.0
**FA2B/FA3**	7.6	0.0	3.0	31.7	12.1	0.0	0.0	0.4	0.0	0.0	0.0
**A3B/A4**	2.4	0.0	0.0	0.0	0.0	0.0	0.0	0.0	0.0	0.0	0.0
**FA3B/FA4**	3.3	0.0	0.0	4.2	2.7	0.0	0.0	0.0	0.0	0.0	0.0
**Digestion**	T	C	T	C	T	T	C	T	T	T	T

	N355	N363	N386	N392	N448	N462	N611	N618	N637	Average
**M4**	1.7	0.0	0.0	0.0	0.1	0.0	0.0	0.0	0.0	0.4
**M5**	28.9	0.0	0.0	0.0	0.5	2.5	0.0	0.5	8.1	5.3
**M6**	10.4	6.2	0.7	1.4	1.6	0.5	0.0	0.0	11.1	4.4
**M7**	13.4	4.9	3.6	5.1	5.7	0.4	0.0	0.0	15.7	6.7
**M8**	4.3	18.7	21.0	28.8	26.2	0.2	0.0	0.0	9.7	13.7
**M9**	0.6	70.2	74.7	64.7	65.5	0.0	0.0	0.0	1.6	36.5
**M9Glc**	0.0	0.0	0.0	0.0	0.3	0.0	0.0	0.0	0.0	0.1
**H**	8.7	0.0	0.0	0.0	0.0	3.3	0.4	0.0	12.1	2.3
**FH**	12.2	0.0	0.0	0.0	0.0	2.6	12.3	9.0	14.1	3.5
**A1**	0.5	0.0	0.0	0.0	0.0	2.7	0.0	0.0	0.7	0.4
**FA1**	2.3	0.0	0.0	0.0	0.0	3.4	0.0	2.9	4.7	0.9
**A1B/A2**	2.2	0.0	0.0	0.0	0.0	1.3	3.5	0.0	0.0	1.7
**FA1B/FA2**	7.9	0.0	0.0	0.0	0.0	18.2	25.2	40.8	16.4	11.2
**A2B/A3**	0.2	0.0	0.0	0.0	0.0	0.3	0.0	0.0	0.0	0.6
**FA2B/FA3**	5.6	0.0	0.0	0.0	0.0	56.3	39.9	23.6	5.6	9.3
**A3B/A4**	0.0	0.0	0.0	0.0	0.0	0.0	0.0	0.0	0.0	0.1
**FA3B/FA4**	1.1	0.0	0.0	0.0	0.0	8.3	18.7	23.2	0.2	3.1
**Digestion**	T	T	C	C	T	T	T	C	T	

Table S7. Primers used for site-directed mutagenesis of BG505.SOSIP.664 trimers, related to Experimental Procedures.

	**Primer sequence (5'-to-3')**
BG505 SOSIP.664 137A	GTACAAACGTGACTAACGCTATCACCGACGATATGCG
BG505 SOSIP.664 197A	CAGGCTGATCAATTGCGCCACCAGTGCCATTACAC
BG505 SOSIP.664 262A	CAGCTGCTGCTGGCTGGATCTCTGGCC
BG505 SOSIP.664 332A	CAGGCACACTGTGCTGTGAGCAAGGCTAC
BG505 SOSIP.664 448A	GTGATTCGATGTGTCAGCGCTATCACAGGCCTGATTC

Table S8. Primers used for site-directed mutagenesis of full-length Env, related to Experimental Procedures.

	**Primer sequence (5'-to-3')**
N133A	CTCTACAGTGTACCGCTGTCACCAATAATATC
N137A	CCAATGTCACCAATGCTATCACTGATGACATG
N156A	GAGGGGAGAATTAAAAGCCTGCTCTTTC
N160K	AACTGCTCTTTCAAGATGACCACAGAGCTAAGG
N190A	ACGAGAATCAAGGTGCTAGGAGTAATAATAGTA
N190cA	CGAGAATCAAGGTGCTAGGAGTAATAATAGTAAC
N197A	GATTAATAAATTGTGCTACCTCAGCCATTAC
N262A	CTCAACTGCTGTTAGCTGGCAGTCTAGCAG
N276A	ATGATTAGATCTGAAGCTATCACAAACAATGC
N295A	ACGCCTGTGCAAATTGCTTGTACCAGACCTAAC
N301A	GTACCAGACCTAACGCCAATACAAGGAAAAG
T332N	GACAAGCACATTGTAATGTCAGTAAAGCAA
N339A	GTCAGTAAAGCAACATGGGCTGAAACTTTGGG
N386A	GAATTTTTCTATTGTGCCACATCAGGCCTGTTC
N392A	CATCAGGCCTGTTCGCTAGCACTTGGATTAG
N448A	AATAAGATGTGTATCAGCCATTACAGGGCTAATA
N611A	TAATGTGCCCTGGGCCTCTAGTTGGAGTAATA
N618A	TTGGAGTAATAGAGCCCTGAGTGAGATATGGG
N625A	AGTGAGATATGGGACGCTATGACCTGGCTGC
N637A	GGGATAAAGAAATTAGCGCCTACACACAGATAA

Supplemental Experimental Procedures

Overview of BG505 constructs

BG505 SOSIP.664 trimers used for site-specific MS analysis were stably expressed in HEK 293T cells as previously described (Chung et al., 2014). The effects of deleting individual glycan sites were studied by making specific mutants of His-tagged BG505 SOSIP.664 trimers (Sanders et al., 2013), which were transiently expressed in HEK 293F cells. SPR experiments were conducted with BG505 SOSIP.664 trimers transiently expressed in 293T cells. Neutralization assays were conducted with Env-pseudoviruses produced in HEK 293T cells. Viruses based on the full-length wild-type BG505 Env glycoprotein (termed here BG505.T332), or the BG505.T332N point mutant in which the N332 glycan site was restored, served as the basis for introducing specific mutations to delete one or two PNGS.

Site-directed mutagenesis

The pPPI4 vector expressing His-tagged BG505 SOSIP.664 has been previously described (Sanders et al., 2013). Single and double mutants of a selection of N-glycan sites in this construct, as well as in the BG505.T332 and BG505.T332N full-length Env proteins, were made by changing asparagine (within the N-glycosylation sequence Asn-X-Ser/Thr, where X is any amino acid except proline) to alanine or (for the N160 site, only) lysine, using the QuikChange Mutagenesis (Agilent) system according to the manufacturer's instructions. Changes were verified by DNA sequencing. The primers used and the list of mutants created are listed in Table S7 and Table S8, respectively.

Expression and purification of Env trimers

BG505 SOSIP.664 trimers used for mass spectrometry analysis of site-specific N-glycosylation were expressed in stable Flp-In HEK 293T cells and purified by 2G12-affinity chromatography followed by SEC, as previously described (Chung et al., 2014). His-tagged BG505 SOSIP.664 trimers and PNGS mutants thereof (see above) were transiently expressed in HEK 293F cells, as described elsewhere (Sanders et al., 2013). The trimers were then purified from cell culture supernatants by PGT151

affinity chromatography as described elsewhere (Pritchard et al., 2015; Ringe et al., 2015). Eluted trimers were immediately buffer exchanged into 10 mM Tris-HCl, 75 mM NaCl (pH 8.0) and concentrated using a 50 kDa cut-off Vivaspin column (GE Healthcare).

Env-pseudovirus production and neutralization assays

To produce Env-pseudoviruses capable of single-cycle replication, HEK 293T cells were co-transfected (PEI, 1 mg/mL, 1:3 PEI:total DNA, Polysciences) with plasmids encoding Env and an Env-deficient genomic backbone (pSG3ΔEnv, 1:2 ratio). Supernatants containing Env-pseudoviruses were harvested 72 h post transfection for use in neutralization assays based on TZM-bl target cells (Li et al., 2005; Montefiori, 2005). Briefly, a serial diluted antibody was pre-incubated with virus for 1 h at 37 °C in a 96-well, flat bottom plate. TZM-bl cells (20,000 cells/well) were added to the virus/antibody mixture and incubated for 72 h. The cells were lysed and luminescence was quantified via addition of Bright-Glo™ Luciferase substrate (Promega). To determine IC_{50} values, dose-response curves were fitted using nonlinear regression (GraphPad Prism).

In-gel release of N-linked glycans

N-linked glycans were enzymatically released from trimers via in-gel digestion. Purified trimers were fractionated by reducing SDS-PAGE (addition of DL-Dithiothreitol; DTT) or by BN-PAGE and stained with Coomassie Blue. Following destaining, the bands corresponding to the trimeric protein (BN-PAGE) or $gp41_{ECTO}$ and gp120 (reducing SDS-PAGE) were excised and washed five times, alternating between acetonitrile and water. The proteins in the gel bands were then digested *in situ* with PNGase F, at 37°C for 16 h, according to the manufacturer's instructions (New England Biolabs). The released glycans were extracted from the gel by washing with water, dried down in a SpeedVac concentrator and then either fluorescently labelled or subjected to IM-ESI MS.

Fluorescent labelling of N-linked glycans and HILIC-UPLC

Released glycans were fluorescently labelled with 2-aminobenzoic acid (2-AA) as previously described (Neville et al., 2009; Pritchard et al., 2015). Excess label was removed using Spe-ed Amide 2 cartridges (iBiosys Solutions Ltd), as described before (Neville et al., 2009). 2-AA labelled glycans were separated by HILIC-UPLC using a 2.1 mm x 10 mm Acquity BEH glycan column (1.7 µm particle size, Waters, Elstree, UK) in a Waters Acquity UPLC instrument. Glycans were separated by running the following gradient: time = 0 min (t = 0): 22 % A, 78 % B (flow rate 0.5 ml/min); t = 38.5: 44.1 % A, 55.9 % B (0.5 ml/min); t = 39.5: 100 % A, 0 % B (0.25 ml/min); t = 44.5: 100 % A, 0 % B; t = 46.5: 22 % A, 78 % B (0.25 ml/min), t = 46.6: 22 % A, 22 % B (0.5 ml/min), where solvent A was 50 mM ammonium formate, pH 4.4, and solvent B was acetonitrile. Fluorescence measuring occurred at an excitation wavelength of 250 nm and a detection wavelength of 428 nm. Empower 3 software was used for data processing.

Endo H digestion of released glycans

Digestion of released glycans with Endo H (New England Biolabs) was employed to measure the abundance of oligomannose-type glycans. The 2-AA labelled glycans were resuspended in water and digested with Endo H for 16 h at 37°C, according to the manufacturer's instructions, then extracted using a PVDF protein-binding membrane plate (Merck Millipore), again according to the manufacturer's instructions, and finally analysed by HILIC-UPLC. The relative abundance of oligomannose-type glycans was determined by integrating the chromatograms with or without Endo H, following normalization.

Tandem ion mobility-ESI MS analysis of released N-linked glycans

The total pool of released glycans from BG505 SOSIP.664 trimers (from the stable HEK 293T cell line) was analysed using ion mobility MS. These results were the basis for the creation of a glycan library that was then used for the subsequent site-specific N-glycosylation analyses. Glycan samples were

desalted with a Nafion membrane for 2 h, as described elsewhere (Börnsen et al., 1995). They were then dissolved in a solution of methanol-water (1:1; vol/vol) containing 100 mM ammonium phosphate. Negative ion mass, collision-induced dissociation (CID) and ion mobility spectra were recorded with a Waters Synapt G2Si mass spectrometer (Waters Corp.) fitted with a nano-electrospray ion source. Samples were infused through Waters thin-wall nanospray capillaries. The instrument was set up as follows: Electrospray capillary voltage: 1.2 kV; cone voltage: 150 V; ion source temperature: 80°C; ion mobility gas (nitrogen) flow: 80 mL/min; T-wave velocity: 450 m/sec; T-wave peak height: 40 V; trap DC bias: 60 V. CID fragmentation was performed after mobility separation in the transfer cell with argon as the collision gas. The collision cell voltage (60-130 V) was adjusted according to the parent ion mass to give an even distribution of fragment ions across the mass range. The instrument was externally mass-calibrated with glucose oligomers (Glc_{2-13}) from dextran (*Leuconostoc mesenteroides*). Samples were digested with α-Neuraminidase (*Clostridium perfringens;* New England Bioscience) if needed. Data acquisition and processing were carried out using the Waters Driftscope (version 2.8) software and MassLynx™ (version 4.1). Spectra were interpreted as described in earlier publications (Harvey, 2005a, b; Harvey, 2005c; Harvey et al., 2008).

Proteolytic digestion of BG505 SOSIP.664 and glycopeptide enrichment

A 200-300 µg sample of trimer in 50 mM Tris/HCl, 6 M Urea, pH 8.0 was used for proteolytic digestions. DTT was added to a final concentration of 5 mM during a 1h incubation at 50°C, followed by addition of 20 mM iodacetamide (IAA), for a further 1h at room temperature (RT) in the dark. Any residual IAA was neutralized by adding 20 mM DTT for 1h at RT. The protein was then buffer exchanged into 50 mM Tris/HCl, pH 8.0 using Vivaspin columns and digested with either trypsin or chymotrypsin (Mass Spectrometry Grade, Promega) at a ratio of 1:20 (w/w) or 1:30 (w/w), respectively, according to the manufacturer's instructions. Protease-digested samples were enriched for glycopeptides by using the ProteoExtract Glycopeptide Enrichment Kit (Merck Millipore) and

dried down in a SpeedVac concentrator. Glycopeptides were then either directly analysed by LC-ESI MS or fractionated by RP-HPLC and subjected to MALDI-TOF MS.

RP-HPLC fractionation of tryptic glycopeptides

Glycopeptides were resuspended in PBS buffer and fractionated by reverse phase-HPLC using a Jupiter C18 5 µm 250 x 4.5 mm column (300 Å, Phenomenex) and a Dionex U3000 LC system. The following gradient was run at a flow rate of 1 ml/min: time = 0 min (t = 0): 95 % Am 5 % B; t = 5: 95 % A, 5 % B; t = 90: 10 % A, 90 % B; t = 95: 10 % A, 90 % B; t = 97: 95 % A, 5 % B, where solvent A was 10 mM ammonium formate, pH 4.4 and solvent B was 10 mM ammonium formate in 80 % acetonitrile. Fractions were collected every minute for 90 min, dried down using a SpeedVac concentrator and resuspended in 20 mM $NaHCO_3$ buffer.

MALDI-TOF MS analysis of glycopeptides

Glycopeptide fractions were divided into two equal fractions. One half was directly analysed MALDI-TOF MS whereas the other was deglycosylated using PNGase F (New England Biolabs). Samples were desalted employing C18 Ziptips according to the manufacturer's instructions and then spotted onto a ground steel target plate in 2,5-dihydrobenzoic acid (DHB, 10 mg/ml in 50 % acetonitrile) matrix. MALDI-TOF MS was performed using an Autoflex^{TM} Speed MALDI-TOF(/TOF) instrument (Bruker), operated in positive ion mode. Tandem MS (MS/MS) was performed on both glycopeptides and peptides to confirm peptide identity. In cases where the same peptide eluted in more than one fraction, the relevant fractions were pooled and reanalysed. Acquired data were processed using DataAnalysis 3 software (Bruker). After baseline subtraction, a signal-to-noise ratio cut-off of 3 was applied. Peptides were manually identified by a combination of 2 approaches. MALDI-TOF MS/MS data were examined for the presence of characteristic glycopeptide fragment ions such as, for example, the $^{0.2}X$ ion $[M_{peptide}+83+H]^+$ or the Y1-ion $[M_{peptide}+203+H]^+$ which then enabled the mass of the peptide portion to be calculated (Wuhrer et al., 2007). In addition, MS and MS/MS data of the corresponding deglycosylated peptide-containing fractions were included in the peptide

identification process. The following peptide modifications were considered: Carbamidomethylation of cysteine, oxidation of methionine, N-terminal Pyro-Glu conversion and deamidation of asparagine, as well as sodium and potassium adducts. As a next step, the glycopeptide spectra were screened for plausible glycopeptide compositions based on the mass of the peptide portion and the generated glycan library. The relative abundance of different glycoforms on each peptide was determined by integrating the intensity of all isotopic peaks, and relating this value to the sum of the intensities of all identified glycoforms.

LC-ESI MS and MS/MS analysis of glycopeptides

Enriched glycopeptides were analysed on a Q-Exactive Orbitrap mass spectrometer (Thermo Fisher Scientific) and glycopeptides were desalted using a Dionex UltiMate 300 RS pump with Acclaim PepMap C18 300 µm ID x 5 mm trap at RT. A flow rate 12 µL/min was used for 4 min using 0.05 % TFA (trifluoroacetic acid) in water as desalting buffer. Desalted peptides were separated using a Dionex UltiMate3000 RSLCnano pump with 75 µm ID x 500 mm analytical columns at 50 ^{o}C. A flow rate of 250 nl/min was used and peptides were eluted using a 2 h linear gradient of 4 % to 40 % buffer B over 120 min. Buffer A was 0.1 % formic acid in water and buffer B was 0.1 % formic acid in acetonitrile. MS data were acquired with XCalibur 3.0.63 (Thermo Fisher Scientific). A Top10 data dependent acquisition method was used. This method allows the selection, fragmentation and detection of ten precursors in a duty cycle time of 1.42 s. Higher energy collisional dissociation (HCD) fragmentation of glycopeptides was performed and led to the generation of low-molecular-weight oxonium ions, peptide fragmentation ions (predominantly y- and b-ions) and B- and Y-type fragmentation of glycosidic linkages (Cao et al., 2014). Data analysis and glycopeptide identification were performed using Byonic^{TM} (Version 2.7) and Byologic^{TM} software (Version 2.3; Protein Metrics Inc.). First, acquired tandem MS spectra were analysed with Byonic, considering the same peptide modifications as for MALDI-TOF MS analysis, a precursor mass tolerance of 4 ppm, a fragment mass tolerance of 10 ppm and two missed cleavages. For tryptic-digested samples, semi-tryptic cleavages

were allowed. Chymotrypsin digestions were specified by C-terminal cleavage of Tyr, Phe, Trp, Leu and Met. The personalized glycan library was used in order to identify the compositions of glycosylated peptides. Byologic was then used for further data processing including relative quantification. Fragmentation spectra of identified glycopeptides were manually verified. The abundance of individual glycoforms was determined by adding up the intensities of the extracted-ion chromatograms (XICs) over all charge states. Relative abundance = [Intensity of one glycoform] / [Intensities of all glycoforms identified on the same peptide].

Surface Plasmon Resonance

Binding of Fabs was analysed by surface plasmon resonance (SPR) on a Biacore 3000 instrument at 25 ^{o}C. Env trimers (transiently expressed in HEK 293T cells and purified by 2G12 affinity chromatography and SEC (Lee et al., 2015)) were immobilized by antibody binding to a His-tag, as previously described (Lee et al., 2015; Yasmeen et al., 2014). Briefly, anti-histidine antibody (GE Healthcare) was amide-coupled to the dextran surface of a CM5 chip up to an immobilization level of 15000 RU. After deactivation, Env trimers were captured to yield R_L values of 520 RU (<2 % s.d.); HBS-EP (10 mM HEPES [pH 7.4], 150 mM NaCl, 3 mM EDTA, 0.002 % P20 surfactant) was used as running buffer. A high flow rate for analyte injections (50 µl/min) was used in order to prevent significant distortion by mass-transport limitation, the near absence of which was confirmed by global fits of mass transfer coefficients. After each cycle the capture-antibody surface was regenerated by an injection of 10 mM Glycine (pH 2) for 120 s at a flow rate of 30 µl/min. The signals from both 0-analyte and control-channel injections were subtracted. A Langmuir model (Biaevaluation, GE Healthcare) was fitted to the binding data as previously described (Yasmeen et al., 2014). S_m values were based on the highest analyte concentrations. All reported binding parameters were significant (T>10).

Supplemental References

Börnsen, K.O., Mohr, M.D., and Widmer, H.M. (1995). Ion exchange and purification of carbohydrates on a Nafion® membrane as a new sample pretreatment for matrix-assisted laser desorption/ionization mass spectrometry. Rapid Commun Mass Spectrom *9*, 1031-1034.

Cao, L., Tolic, N., Qu, Y., Meng, D., Zhao, R., Zhang, Q., Moore, R.J., Zink, E.M., Lipton, M.S., Pasa-Tolic, L.*, et al.* (2014). Characterization of intact N- and O-linked glycopeptides using higher energy collisional dissociation. Anal Biochem *452*, 96-102.

Chung, N.P.Y., Matthews, K., Kim, H.J., Ketas, T.J., Golabek, M., de Los Reyes, K., Korzun, J., Yasmeen, A., Sanders, R.W., Klasse, P.J.*, et al.* (2014). Stable 293 T and CHO cell lines expressing cleaved, stable HIV-1 envelope glycoprotein trimers for structural and vaccine studies. Retrovirology *11*, 33-33.

Harvey, D.J. (2005a). Fragmentation of negative ions from carbohydrates: part 1. Use of nitrate and other anionic adducts for the production of negative ion electrospray spectra from N-linked carbohydrates. J Am Soc Mass Spectrom *16*, 622-630.

Harvey, D.J. (2005b). Fragmentation of negative ions from carbohydrates: part 2. Fragmentation of high-mannose N-linked glycans. J Am Soc Mass Spectrom *16*, 631-646.

Harvey, D.J. (2005c). Fragmentation of negative ions from carbohydrates: part 3. Fragmentation of hybrid and complex N-linked glycans. J Am Soc Mass Spectrom *16*, 647-659.

Harvey, D.J., Merry, A.H., Royle, L., Campbell, M.P., and Rudd, P.M. (2011). Symbol nomenclature for representing glycan structures: Extension to cover different carbohydrate types. Proteomics *11*, 4291-4295.

Harvey, D.J., Royle, L., Radcliffe, C.M., Rudd, P.M., and Dwek, R.A. (2008). Structural and quantitative analysis of N-linked glycans by matrix-assisted laser desorption ionization and negative ion nanospray mass spectrometry. Anal Biochem *376*, 44-60.

Lee, J.H., Leaman, D.P., Kim, A.S., Torrents de la Peña, A., Sliepen, K., Yasmeen, A., Derking, R., Ramos, A., de Taeye, S.W., Ozorowski, G., *et al.* (2015). Antibodies to a conformational epitope on gp41 neutralize HIV-1 by destabilizing the Env spike. Nature communications *6*, 8167-8167.

Li, M., Gao, F., Mascola, J.R., Stamatatos, L., Polonis, V.R., Koutsoukos, M., Voss, G., Goepfert, P., Gilbert, P., Greene, K.M., *et al.* (2005). Human immunodeficiency virus type 1 env clones from acute and early subtype B infections for standardized assessments of vaccine-elicited neutralizing antibodies. J Virol *79*, 10108-10125.

Montefiori, D.C. (2005). Evaluating neutralizing antibodies against HIV, SIV, and SHIV in luciferase reporter gene assays. Curr Protoc Immunol *12*, 11.11-11.17.

Neville, D.C., Dwek, R.A., and Butters, T.D. (2009). Development of a single column method for the separation of lipid- and protein-derived oligosaccharides. J Proteome Res *8*, 681-687.

Pritchard, L.K., Vasiljevic, S., Ozorowski, G., Seabright, G.E., Cupo, A., Ringe, R., Kim, H.J., Sanders, R.W., Doores, K.J., Burton, D.R., *et al.* (2015). Structural constraints determine the glycosylation of HIV-1 envelope trimers. Cell Rep *11*, 1604-1613.

Ringe, R.P., Yasmeen, A., Ozorowski, G., Go, E.P., Pritchard, L.K., Guttman, M., Ketas, T.A., Cottrell, C.A., Wilson, I.A., Sanders, R.W., *et al.* (2015). Influences on the design and purification of soluble, recombinant native-like HIV-1 envelope glycoprotein trimers. J Virol *89*, 12189-12210.

Sanders, R.W., Derking, R., Cupo, A., Julien, J.P., Yasmeen, A., de Val, N., Kim, H.J., Blattner, C., de la Pena, A.T., Korzun, J., *et al.* (2013). A next-generation cleaved, soluble HIV-1 Env trimer, BG505 SOSIP.664 gp140, expresses multiple epitopes for broadly neutralizing but not non-neutralizing antibodies. PLoS Path *9*, e1003618.

Wuhrer, M., Catalina, M.I., Deelder, A.M., and Hokke, C.H. (2007). Glycoproteomics based on tandem mass spectrometry of glycopeptides. J Chromatogr B Analyt Technol Biomed Life Sci *849*, 115-128.

Yasmeen, A., Ringe, R., Derking, R., Cupo, A., Julien, J.P., Burton, D.R., Ward, A.B., Wilson, I.A., Sanders, R.W., Moore, J.P., *et al.* (2014). Differential binding of neutralizing and non-neutralizing antibodies to native-like soluble HIV-1 Env trimers, uncleaved Env proteins, and monomeric subunits. Retrovirology *11*, 41.
