# Supplementary material for: Composition and Antigenic Effects of Individual Glycan Sites of a Trimeric HIV-1 Envelope Glycoprotein
Source: Cell Rep. 2016 Mar 10;14(11):2695–706. doi: 10.1016/j.celrep.2016.02.058 (PMC4805854; doi:10.1016/j.celrep.2016.02.058)
Supplement: Document S2. Article plus Supplemental Information [file mmc6.pdf]

Cell Reports

Article

Composition and Antigenic Effects of Individual Glycan Sites of a Trimeric HIV-1 Envelope Glycoprotein

Graphical Abstract

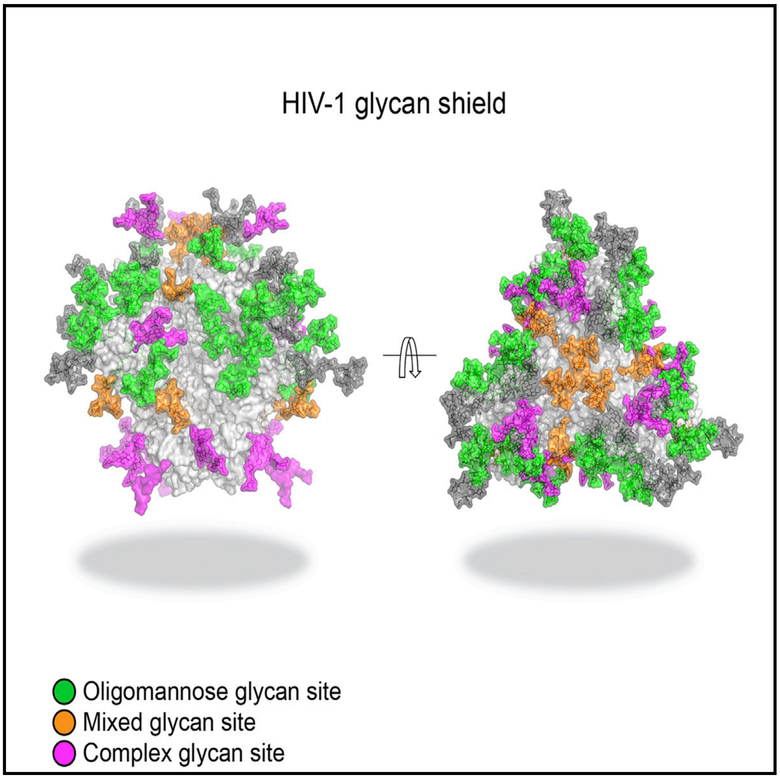

Highlights

- Quantitative, site-specific N-glycan analysis of a soluble HIV-1 Env trimer
- A map of the extremes of simplicity and diversity at individual glycan sites
- The fine structure of the mannose patch area of the Env trimer
- How individual glycan sites influence HIV-1 Env-pseudovirus neutralization

Authors

Anna-Janina Behrens, Snezana Vasiljevic, Laura K. Pritchard, ..., John P. Moore, Katie J. Doores, Max Crispin

Correspondence

katie.doores@kcl.ac.uk (K.J.D.), max.crispin@bioch.ox.ac.uk (M.C.)

In Brief

Behrens et al. present detailed, quantitative, site-specific analyses of N-glycosylation sites of a soluble recombinant HIV-1 envelope glycoprotein trimer. The results highlight structural and antigenic details of the glycan shield that will be valuable for designing next-generation HIV-1 Env vaccines and understanding virus neutralization by broadly active antibodies.

Behrens et al., 2016, Cell Reports *14*, 2695–2706
March 22, 2016 ©2016 The Authors
http://dx.doi.org/10.1016/j.celrep.2016.02.058

Cell Reports
Article

OPEN ACCESS
CellPress

Composition and Antigenic Effects of Individual Glycan Sites of a Trimeric HIV-1 Envelope Glycoprotein

Anna-Janina Behrens,[1] Snezana Vasiljevic,[1] Laura K. Pritchard,[1] David J. Harvey,[1] Rajinder S. Andev,[2] Stefanie A. Krumm,[2] Weston B. Struwe,[1] Albert Cupo,[3] Abhinav Kumar,[1] Nicole Zitzmann,[1] Gemma E. Seabright,[1] Holger B. Kramer,[4] Daniel I.R. Spencer,[5] Louise Royle,[5] Jeong Hyun Lee,[6] Per J. Klasse,[3] Dennis R. Burton,[7,8] Ian A. Wilson,[6,9] Andrew B. Ward,[6] Rogier W. Sanders,[3,10] John P. Moore,[3] Katie J. Doores,[2,*] and Max Crispin[1,*]

[1]Oxford Glycobiology Institute and Department of Biochemistry, University of Oxford, South Parks Road, Oxford OX1 3QU, UK
[2]Department of Infectious Diseases, Faculty of Life Sciences and Medicine, King's College London, Guy's Hospital, London SE1 9RT, UK
[3]Department of Microbiology and Immunology, Weill Cornell Medical College, New York, NY 10021, USA
[4]Department of Physiology, Anatomy and Genetics, University of Oxford, South Parks Road, Oxford OX1 3QX, UK
[5]Ludger, Ltd., Culham Science Centre, Abingdon, Oxfordshire OX14 3EB, UK
[6]Department of Integrative Structural and Computational Biology, International AIDS Vaccine Initiative (IAVI) Neutralizing Antibody Center and CAVD, Center for HIV/AIDS Vaccine Immunology and Immunogen Discovery, The Scripps Research Institute, La Jolla, CA 92037, USA
[7]Department of Immunology and Microbial Science, IAVI Neutralizing Antibody Center and CAVD, Center for HIV/AIDS Vaccine Immunology and Immunogen Discovery, the Scripps Research Institute, La Jolla, CA 92037, USA
[8]Ragon Institute of Massachusetts General Hospital, Massachusetts Institute of Technology and Harvard University, Boston, MA 02142, USA
[9]Skaggs Institute for Chemical Biology, The Scripps Research Institute, La Jolla, CA 92037, USA
[10]Laboratory of Experimental Virology, Department of Medical Microbiology, Center for Infection and Immunity Amsterdam (CINIMA), Academic Medical Center of the University of Amsterdam, 1105 AZ Amsterdam, the Netherlands
*Correspondence: katie.doores@kcl.ac.uk (K.J.D.), max.crispin@bioch.ox.ac.uk (M.C.)
http://dx.doi.org/10.1016/j.celrep.2016.02.058
This is an open access article under the CC BY license (http://creativecommons.org/licenses/by/4.0/).

SUMMARY

The HIV-1 envelope glycoprotein trimer is covered by an array of N-linked glycans that shield it from immune surveillance. The high density of glycans on the trimer surface imposes steric constraints limiting the actions of glycan-processing enzymes, so that multiple under-processed structures remain on specific areas. These oligomannose glycans are recognized by broadly neutralizing antibodies (bNAbs) that are not thwarted by the glycan shield but, paradoxically, target it. Our site-specific glycosylation analysis of a soluble, recombinant trimer (BG505 SOSIP.664) maps the extremes of simplicity and diversity of glycan processing at individual sites and reveals a mosaic of dense clusters of oligomannose glycans on the outer domain. Although individual sites usually minimally affect the global integrity of the glycan shield, we identify examples of how deleting some glycans can subtly influence neutralization by bNAbs that bind at distant sites. The network of bNAb-targeted glycans should be preserved on vaccine antigens.

INTRODUCTION

The trimeric HIV type 1 (HIV-1) envelope glycoprotein (Env) is the sole target for broadly neutralizing antibodies (bNAbs) produced by the immune system during infection and is, therefore, a focus of vaccine design. In numerous studies, bNAbs provide passive protection from viral challenge to non-human primates (Hessell et al., 2010; Mascola et al., 2000; Moldt et al., 2012). Many of these bNAbs recognize epitopes that are wholly or partially composed of glycan structures (Blattner et al., 2014; Calarese et al., 2003; Falkowska et al., 2014; Garces et al., 2014; Huang et al., 2014; Kong et al., 2013; McLellan et al., 2011; Mouquet et al., 2012; Pancera et al., 2013; Pejchal et al., 2011; Scanlan et al., 2002; Scharf et al., 2014; Walker et al., 2011). HIV-1 Env is among the most heavily glycosylated proteins known, with glycans making up ~50% of its total mass (Lasky et al., 1986). These abundant glycans have long been considered to shield the trimer from immune surveillance by occluding relatively conserved protein surfaces (Wei et al., 2003); while this concept remains valid, it is also now evident that the glycan shield itself can be a target for bNAbs. Defining the detailed composition of the glycan shield will increase our understanding of bNAb epitopes and how HIV-1 is neutralized and, thus, help the rational design of Env-based vaccine immunogens.

The Env trimer is composed of three gp120 and three gp41 subunits. Analyses of monomeric gp120 proteins have revealed the presence of under-processed N-glycans that remain in oligomannose form ($Man_{5-9}GlcNAc_2$) because steric constraints impede the actions of the endoplasmic reticulum (ER) and Golgi α-mannosidases (Bonomelli et al., 2011; Doores et al., 2010; Go et al., 2013; Leonard et al., 1990; Zhu et al., 2000). These oligomannose-type glycans are mainly localized to a highly conserved area of the gp120 outer domain, the so-called "intrinsic mannose patch,"

that presents numerous bNAb epitopes (Calarese et al., 2003; Doores, 2015; Doores et al., 2015; Kong et al., 2013; Pritchard et al., 2015c; Scanlan et al., 2002; Walker et al., 2009, 2011). Because these studies were performed using recombinant gp120 monomers, it was uncertain at the time to what extent their glycan content mimicked the native, trimeric structure on HIV-1 virions (Coutu and Finzi, 2015; Klasse et al., 2013; Kovacs et al., 2014; Ringe et al., 2013, 2015). Virion-derived trimers are hard to obtain in sufficient quantities for detailed characterizations, but oligomannose-rich regions are known to be present on both Envs extracted from HIV-1 virions (Bonomelli et al., 2011; Doores et al., 2010; Pritchard et al., 2015a) and membrane-associated recombinant Env (Go et al., 2015). Nonetheless, major knowledge gaps remained to be filled.

The soluble, recombinant BG505 SOSIP.664 trimer is the prototype of a class of native-like, Env-mimetic immunogens that is now being pursued in various vaccine-development programs (Sanders et al., 2013, 2015). On the BG505 SOSIP.664 trimers, the glycosylation profile of the gp120 subunits is dominated by large, oligomannose-type structures of the $Man_{8-9}GlcNAc_2$ type, while more complex-type structures are found on gp41 (Pritchard et al., 2015d). The native-like quaternary structure of the SOSIP.664 trimer had a major influence on its oligomannose-rich glycosylation profile; simpler (gp120 monomer) or non-native (uncleaved gp140) Env proteins carry a much higher content of processed glycans (Pritchard et al., 2015d; Ringe et al., 2015). These findings led to the concept of the "trimer-associated mannose patch" (TAMP) (Crispin and Doores, 2015).

Here, a quantitative, site-specific N-glycosylation analysis reveals the fine structures of the glycan shield of the BG505 SOSIP.664 trimer. Our results confirm the remarkable overall dominance of oligomannose-type glycans and reveal a mosaic of glycan microclusters bearing under-processed glycans, especially in areas covering the gp120 outer domain and at the trimer interfaces. At the trimer apex, there is a microcluster of glycans of mixed processing states, with both under-processed and complex structures present. In contrast, highly variable, but also highly processed, complex glycans occupy the sites present on gp41 and on the most proximal gp120 regions at the trimer base. Deleting specific potential N-glycosylation sites (PNGSs) does not markedly affect the overall glycosylation profile of the BG505 SOSIP.664 trimer but can directly or indirectly influence the neutralization sensitivity of HIV-1 BG505 Env-pseudotyped viruses.

RESULTS AND DISCUSSION

A Glycan Library from BG505 SOSIP.664 Trimers

The design and structure of the native-like BG505 SOSIP.664 trimer have been described elsewhere and are summarized in Figure 1A (Binley et al., 2000; Julien et al., 2013b; Khayat et al., 2013; Klasse et al., 2013; Lyumkis et al., 2013; Sanders et al., 2002, 2013). We first produced BG505 SOSIP.664 trimers from a stable HEK293T cell line as previously described (Chung et al., 2014). We analyzed the enzymatically released and fluorescently labeled N-glycans by hydrophilic interaction chromatography-ultra-performance liquid chromatography (HILIC-UPLC) to determine the trimer's overall glycan profile (Figure 1B). Endoglycosidase H (Endo H) cleavage of the released glycan pool established that 63% of the total glycans were of the oligomannose type.

To probe the glycan structures in greater detail and to facilitate the processing of subsequent site analysis data, we generated a database of the N-glycans present. For this purpose, unlabeled glycans released from the gp120 and gp41 subunits were analyzed separately by ion mobility-electrospray ionization mass spectrometry (IM-ESI MS; Figure 1C). Glycans were identified, and isomeric structures were assigned using negative ion fragmentation mode (Harvey et al., 2011). In total, 52 and 59 isobaric structures were identified on gp120 and gp41, respectively (Table S1). Consistent with the UPLC data, a significant oligomannose population was present on gp120. We obtained fine structural details of the highly processed gp41 glycans, which were dominated by unsialylated galactose-terminating bi- and triantennary structures (Figure 1C).

Overall, the mass spectrometry (MS) data reveal the extremes of glycan processing and protection that are displayed across the surface of the densely glycosylated trimer. The site-specific variation in glycan structures arises from the intersection between the folding of Env into its quaternary structure and how the producer cell's processing enzymes then see the folded trimer.

Quantitative Site-Specific Glycan Analysis

To quantify the distribution of under-processed oligomannose-type glycans and specify the locations of the highly processed complex-type glycans, we used a parallel mass-spectrometric-based approach exploiting two different ionization modes.

In one strategy, we generated glycopeptides from BG505 SOSIP.664 trimers by trypsin digestion, fractionated them by reverse-phase high-performance liquid chromatography (RP-HPLC), and analyzed the resulting glycopeptide pools using MALDI-TOF MS. In total, 11 glycan sites could be isolated and quantified by assessment of the ion abundances (Figure S1; Tables S2 and S3). In an alternative approach, the total pool of trypsin- and chymotrypsin-digested glycopeptides was analyzed by coupled, in-line liquid chromatography-ESI (LC-ESI) MS. Here again, we determined the relative abundance of each specific glycan structure by summing the ion intensity of the corresponding glycopeptide over all identified charge states. This quantification procedure is justified, because protonation of glycopeptides occurs mainly on the peptide backbone; the method has been validated previously, albeit using simpler targets (Wada, 2013). Nevertheless, our complementary analytical approaches enabled an additional validation of the relative quantification performed here.

The glycan compositions derived by the two methods are compared in Figure 2, which shows three representative N-glycan sites corresponding to those dominated by oligomannose structures, complex-type structures, or a mixed population of both glycan classes. The two datasets are highly concordant, indicating that each method reliably captures the processing state of an isolated glycosylation site. Using the glycan database generated by the ion mobility MS of released glycans (Figure 1), analysis of the LC-ESI MS data allowed 20 occupied N-linked glycosylation sites to be characterized in detail (Figure 3; Tables S4, S5, and S6). The remaining eight sites could not be

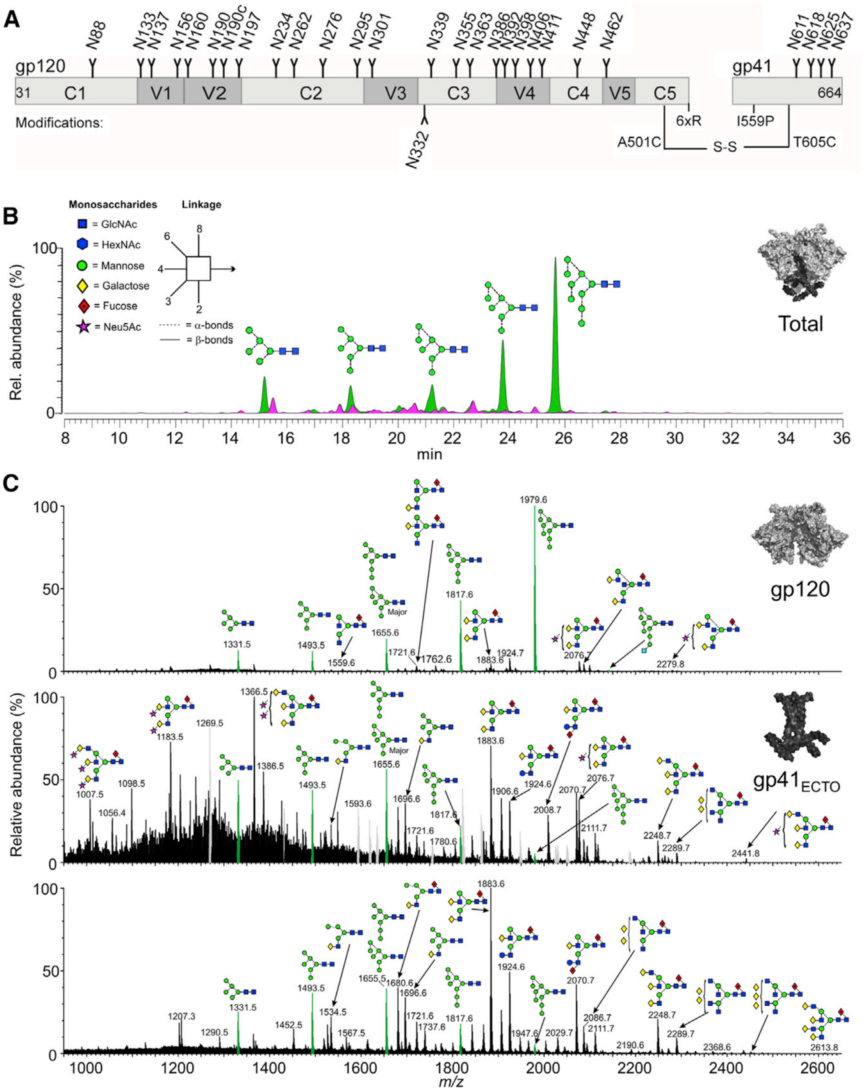

Figure 1. Glycosylation Pattern of BG505 SOSIP.664 Trimers

(A) Schematic representation of the BG505 SOSIP.664 construct, including PNGSs and incorporated modifications.

(B) HILIC-UPLC profile of N-linked glycans from BG505 SOSIP.664 trimers stably produced in HEK293T cells and purified by 2G12-affinity chromatography/SEC. Oligomannose-type and hybrid glycans (green) were identified by their sensitivity to Endo H digestion. Pink indicates complex glycans. Rel., relative.

(C) Negative ion electrospray spectra of N-glycans found on the gp120 (upper panel) and $gp41_{ECTO}$ (middle panel) subunits of BG505 SOSIP.664 trimers; and mobility-extracted singly charged negative ions from desialylated $gp41_{ECTO}$ glycans (bottom). Symbols are as explained in (B).

See also Table S1.

quantified; they were present either on a peptide containing more than one N-glycan (e.g., N133 and N137) or on a glycopeptide that could not be identified with sufficient confidence (e.g., N398 and N625). The latter scenario is likely to be attributable to either incomplete occupancy of the N-glycosylation site or a low ionization efficiency.

The overall percentages of oligomannose-type versus processed glycans confirm that the glycosylation sites on the gp120 subunits are largely dominated by oligomannose-type structures, whereas more highly processed glycans dominate on gp41. However, there are exceptions to these generalizations on both subunits, which we explore later. We generated a map of the distribution of the different processing states across the BG505 SOSIP.664 trimer by constructing a model based on its cryoelectron microscopy (cryo-EM) structure at a 4.36-Å resolution (Lee et al., 2015) (Figure 3B). The model reveals a mosaic of sites bearing oligomannose-type glycans, complex glycans, or a mixture of both (green, pink, and orange, respectively; Figure 3).

However, the distribution is not random, and a network of oligomannose glycans is evident across the trimer surface. The heterogeneity of the glycan-processing states indicates that there must be hotspots of accessibility to the producer cell's glycan-processing enzymes. In the following text, we discuss examples of how even closely proximal glycans can differ substantially in their processing states.

Clusters of Under-processed Glycans

The trimer apex is covered by a ring of six glycans created by two individual sites (N156 and N160) from the V1/V2 regions of each of the three protomers. All six glycans in this ring are minimally processed, which we attribute to inter-glycan interactions among these topologically proximal structures that shield each of them from the processing enzymes. $Man_9GlcNAc_2$ moieties dominate at the N156 position, whereas the N160 glycan is slightly more processed, although still predominantly of the oligomannose type (Figure 3). Glycans N234 and N276 near the CD4-binding site (CD4bs) also behave as a linked, minimally processed pair on each individual gp120 protomer, and we note that they are separated at the Asn Cα positions by only 10.6 Å (PDB: 5ACO) (Lee et al., 2015). This degree of proximity may again be sufficient for bidirectional protection from enzymatic processing, albeit with one partner again being slightly more shielded than the other. Thus, here, N276 is partially oligomannose-trimmed toward $Man_5GlcNAc_2$, with some hybrid and small complex structures also present, whereas N234 is even less processed. The way in which glycans proximal to the CD4bs are processed has been proposed to influence how antibodies interact with this critical region of Env (Kong et al., 2010). In this general context, it is relevant that Fab fragments of several CD4bs-targeting antibodies bind

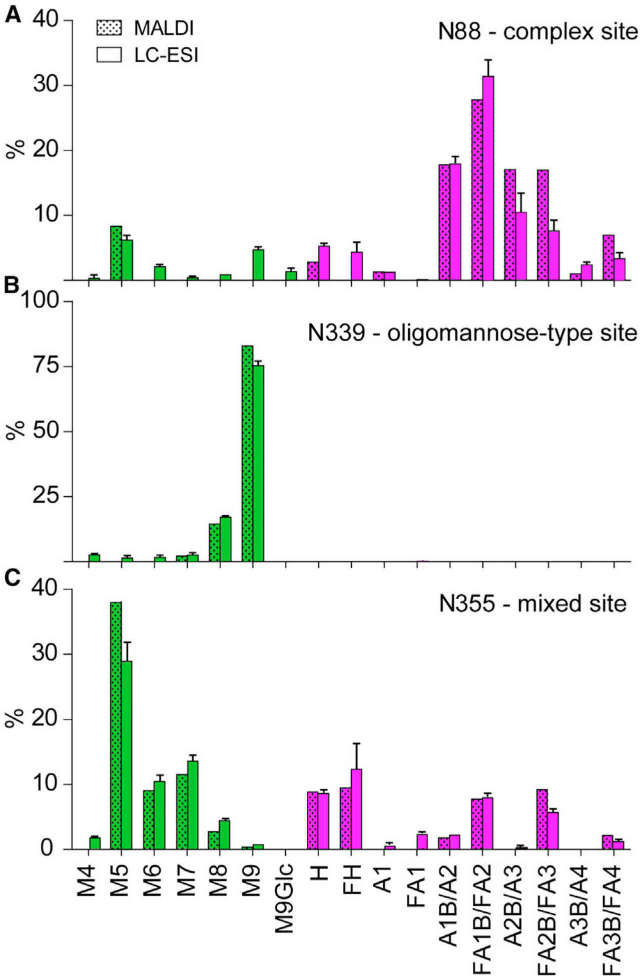

Figure 2. Comparison of Glycan Abundances Determined by MALDI-TOF MS and LC-ESI MS

(A–C) Side-by-side bar graph representation of the relative glycan abundances determined by MALDI-TOF MS and LC-ESI MS for three representative sites identified by trypsin digests. Shown are examples of N-glycosylation sites dominated by (A) complex-type glycans (pink; N88), (B) oligomannose glycans (green; N339), or (C) a mixture of structures (N355). Repeats of the LC-MS ESI analysis were performed, and the SEM is indicated on the bar graphs. Figure S1 shows the quantification of the remaining glycosylation sites isolated and analyzed by MALDI-TOF MS. Glycans were grouped as shown in Table S2 and quantified by summing up ion intensities. Briefly, the grouping is based on the number of residues within the oligomannose series (M) or the number of antennae (A) with and without core fucose (F) in a complex glycan.
See also Tables S3, S4, and S5.

markedly faster, but also with higher off-rate constants, to BG505 SOSIP.664 trimer mutants lacking the N276 glycan that abuts the CD4bs (Figure S2). A further example of how the dense clustering of glycans may sterically impede the access of glycan-processing enzymes to their substrates involves the mannose patch on the gp120 outer domain (Figure 3B).

Fine Structure of the Mannose Patch

Two densely packed microclusters are centered on residues N295 and N392 that, together, form a large patch of predominantly $Man_9GlcNAc_2$ glycans (Figure 3B). Here, the N295 glycan is intimately embraced by its counterparts at positions N262, N448, N332, and N301. We were unable to characterize the N301 glycan in this analysis, but oligomannose-type glycans have previously been identified at this position (Guttman et al., 2014). The N392, N363, and N386 glycans form a closely packed microcluster, with their Cα atoms as close together as 7.1 Å (N363 to N392), 8.8 Å (N363 to N386), and 11.5 Å (N386 to N392) (PDB: 5ACO) (Lee et al., 2015). This roughly triangular array of glycans is further surrounded by those on N137, N197, N133, and N339. The N332 glycan, a key component of the so-called "supersite" of immune vulnerability (Kong et al., 2013; Mouquet et al., 2012; Pejchal et al., 2011; Walker et al., 2011), resides at the heart of this site. Its location between the two component microclusters links them into a single large oligomannose patch that stretches around the entire gp120 outer domain. The N332 glycan itself is in close contact with, or in close proximity to, N295, N301, and N137. Overall, the mannose patch is stabilized by the now-revealed glycan-glycan interactions within and/or between the two individual microclusters.

Interprotomer Control of Glycan Processing

In addition to glycan clustering effects, we also found regions of the trimer where interprotomer interactions and the resulting steric effects are likely to limit glycan processing. For example, N197 bears a mix of oligomannose-type and complex N-glycans and resides very close to the V3 region of gp120 from the adjacent protomer (Figure 3B). As such interactions could only occur in the context of a properly assembled trimer, our observation is not inconsistent with reports that the N197 glycan of monomeric gp120 proteins contains complex glycans (Go et al., 2013; Leonard et al., 1990; Pritchard et al., 2015b). The same general scenario applies to the N156, N160, and N276 glycans that are complex when in the context of monomeric gp120 (Go et al., 2013; Leonard et al., 1990; Zhu et al., 2000). We now show that each of these glycans is predominantly of the oligomannose type when present on the BG505 SOSIP.664 trimer. Hence, all of these sites are subject to the additional constraints imposed on processing enzymes by the interprotomer interactions that apply at the trimer level but that are irrelevant to gp120 monomers (Pritchard et al., 2015d). Overall, our analyses reveal key molecular features of the TAMP (Crispin and Doores, 2015).

Processing of the Exposed Glycans of the Trimer Base

Complex glycans dominate the N and C termini of the BG505 SOSIP.664 trimer, which are located very close to one another at the structure's base (Figure 3). In particular, the highly processed gp120 N88 glycan is located immediately proximal to the gp41 subunit. Of the three glycan sites identified on gp41, N611 and N618 display a broad range of complex glycans (Figure 3; Tables S4 and S5), whereas N637 is less extensively processed; a significant proportion of these structures are in oligomannose-type form. An explanation for the mixed-processing status of the N637 glycan could be trimer-induced steric hindrance caused by its proximity to gp120 glycans N234 and N276. The comparatively limited (relative to

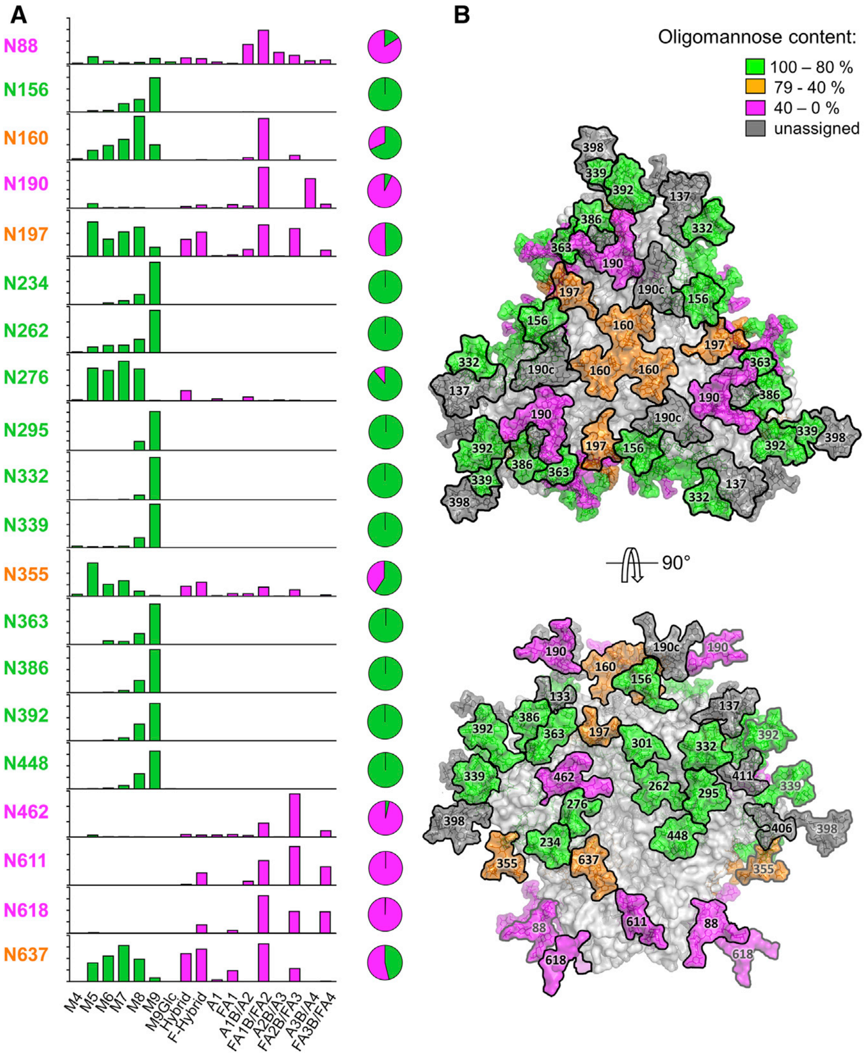

Figure 3. Site-Specific Glycosylation Profiles of BG505 SOSIP.664 Trimers

(A) Relative quantification of 20 N-glycosylation sites of BG505 SOSIP.664 trimers, stably produced in HEK293T cells. The trimers were digested with trypsin or chymotrypsin, enriched for glycopeptides, and analyzed by LC-ESI MS. Glycans were grouped as shown in Table S2. The bar graphs represent the means of two analytical replicates; the pie charts summarize the quantification of oligomannose-type (green) and complex/hybrid glycans (pink) on individual sites. Quantifications are based on the peak lists in Tables S4 and S5. Percentages corresponding to this figure can be found in Table S6.

(B) Model of a fully glycosylated BG505 SOSIP.664 trimer. The model was constructed using PDB: 5ACO (Lee et al., 2015), with the following glycans modeled according to the processing state: oligomannose-type ($Man_9GlcNAc_2$; Woods et al., 1998), processed (galactosylated biantennary glycan; from PDB: 1L6X), and mixed ($Man_5GlcNAc_2$; Woods et al., 1998) sites. N301 is classified as an oligomannose-type glycan based on the cryo-EM structure (Lee et al., 2015) and previously published MS analysis (Guttman et al., 2014). Deleting the nearby glycan at residue N276 increases both the on- and off-rate constants of the binding of CD4bs-targeting antibodies (Figure S2).

See also Figure S2 and Tables S2, S4, and S6.

gp120) overall oligomannose content of the gp41 subunit that was identified by UPLC analysis (Pritchard et al., 2015d) may, therefore, originate from only a single site, N637. We were only able to identify trace amounts of glycosylated peptides corresponding to the N625 site that were insufficient to verify by tandem MS (MS/MS). Hence, the N625 glycan site may be markedly less occupied than the other gp41 sites. There are previous reports of the partial or alternative occupancy of gp41 glycan sites in general (Depetris et al., 2012; Go et al., 2011, 2015; Pabst et al., 2012) and of the BG505 SOSIP.664 trimer's N625 glycan in particular (Guttman et al., 2014).

The BG505 SOSIP.664 trimer used for these glycan site analyses is a soluble protein. It lacks not only the transmembrane region of gp41 but also the membrane-proximal external region (MPER) (Khayat et al., 2013; Klasse et al., 2013). We note that the highly processed N611 and N618 glycans are each likely to be located very close to where the MPER would be located. Thus, in the context of full-length Env, additional shielding effects created by the MPER and the virion or cell membrane may provide additional constraints on processing of the nearby N611 and N618 sites. Even though the overall glycosylation profile of a soluble SOSIP.664 trimer and a sequence-matched, membrane-associated Env ΔCT trimer are broadly similar (Pritchard et al., 2015d), there may be structurally explicable differences in how specific sites are processed in the different contexts.

The Mannose Patch Is Reshaped by the Presence of a Glycan at N332

The N332 glycan at the heart of the mannose patch on the BG505 SOSIP.664 trimer is clearly dominated by $Man_9GlcNAc_2$ moieties, as shown both by MALDI-TOF MS (Figures 4A and 4B) and LC-ESI MS (Figures 4C and 4D). We note that the transmitted/founder virus isolated from the BG505 infant 6 weeks after birth does not contain an N-glycosylation site at position N332 (Sanders et al., 2013; Wu et al., 2006). The SOSIP.664 trimer is based on a clone from the week-6 isolate, but with the N332 glycan specifically introduced to create epitopes for the multiple bNAbs that recognize it (Kong et al., 2013; Pejchal et al., 2011; Scanlan et al., 2002; Sok et al., 2014; Walker et al., 2011). To assess the impact of the introduced N332 glycan, we expressed and analyzed BG505 SOSIP.664 (i.e., N332 present) trimers and its N332A mutant in HEK293F cells and determined their glycan profiles. Comparing the resulting

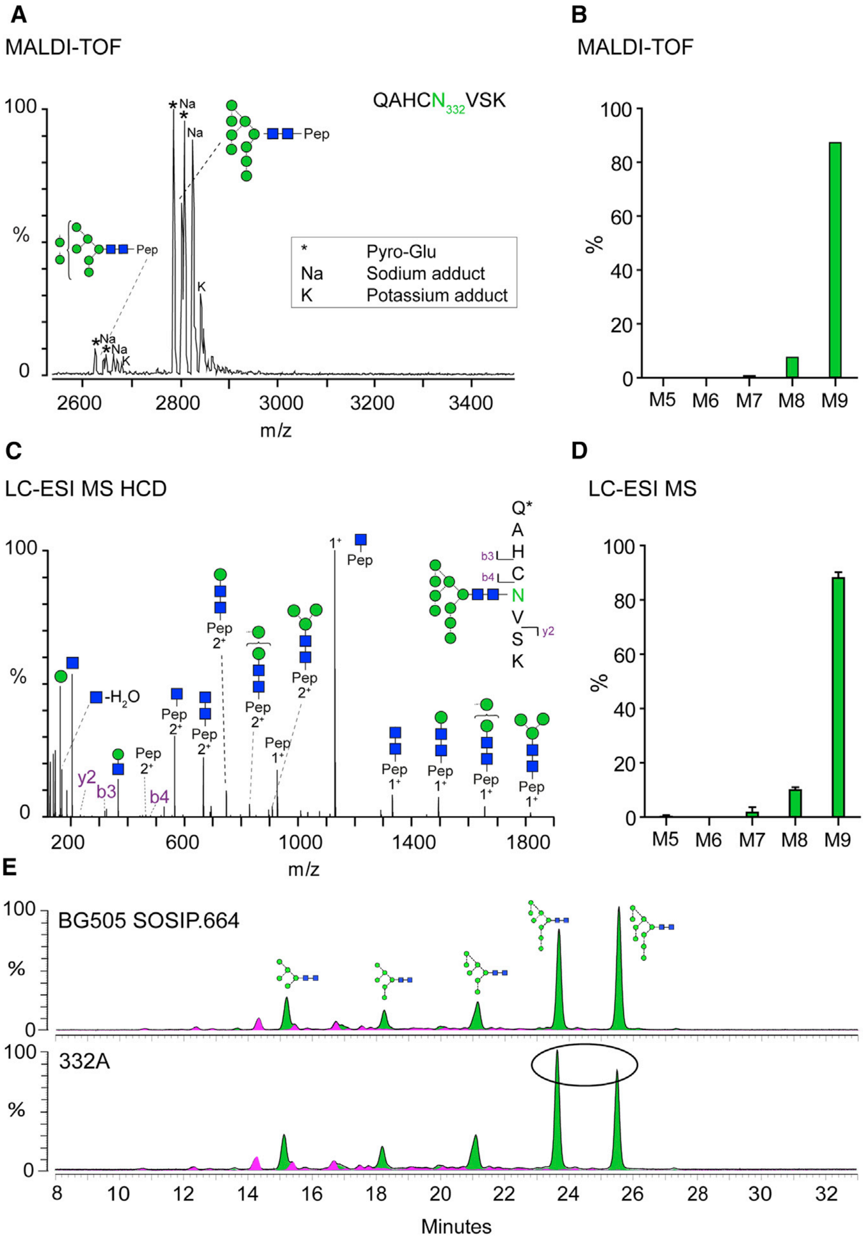

Figure 4. Glycosylation of N332 and Its Effect on the Glycan Shield

(A) MALDI-TOF MS spectrum of a tryptic glycopeptide containing N332. Observed modifications are indicated in the box and highlighted in the spectrum.

(B) Quantification of peak areas observed in the MALDI-TOF spectrum.

(C) LC-ESI MS HCD fragmentation of the same tryptic peptide (Pep) containing N332.

(D) Quantification of ion intensities for glycans identified for N332 and analyzed by LC-ESI MS. Error bars represent SEM.

(E) HILIC-UPLC profiles of glycans released from BG505 SOSIP.664 transiently produced in HEK293F cells and purified by PGT151-affinity chromatography. Bottom spectrum: alanine-mutated PNGS N332. Green indicates oligomannose-type and hybrid glycans; pink indicates complex glycans. Oval indicates region of spectrum exhibiting changes upon N332A mutation.

two HILIC-UPLC profiles shows that the knocked-in N332 glycan reshapes the overall glycan profile. Thus, compared to the standard trimer, the glycans released from the N332A mutant contain fewer $Man_9GlcNAc_2$ moieties, but more $Man_8GlcNAc_2$, so that the latter was now the most prominent N-glycan structure (Figure 4E). We interpret this observation to mean that, when the N332 glycan is present, it influences the composition of the multiple, closely proximal glycans within the mannose patch. A likely mechanism for this effect is steric obstruction of α-mannosidase enzymes (Pritchard et al., 2015b). More generally, we propose that, when the glycan shield evolves under immune selection pressure during HIV-1 infection, there may be subtle influences on the glycan composition of sites located some distance away from the point at which a glycan is added or deleted.

Effect of Glycan Site Deletions on Neutralization Sensitivity and Structure Integrity

To determine the impact of glycan deletions on bNAb sensitivity, we removed various PNGSs from the BG505.T332 (i.e., wild-type BG505) and the BG505.T332N Env-pseudotyped viruses (note that the BG505.T332N virus has the same glycosylation sites as BG505 SOSIP.664 trimers) (Sanders et al., 2013, 2015). Then, we determined neutralization profiles for a range of bNAbs to multiple epitopes: CD4bs (PGV04), mannose-patch binding (PGT121, PGT128, PGT130, and PGT135), V1/V2 loop (PG9 and PGT145), and gp120/gp41 interface (PGT151) (Figure 5). As expected, when glycans known to be key components of epitopes were absent, bNAb sensitivity was substantially reduced or lost entirely. For example, removing the N156 or N160 glycans from either of the BG505 test viruses reduced the neutralization activities of PG9 and PGT145; likewise, for glycans N295 or N301 with PGT128 and PGT130. However, we also observed some unexpected outcomes. Thus, eliminating the N197 glycan substantially reduced (BG505.T332) or entirely ablated (BG505.T332N) the neutralization activity of the V1/V2-binding bNAb PGT145. Similarly, deleting the N197 glycan from the BG505.T332 virus also reduced its sensitivity to PG9. We note that the N197 site contains a mixture of glycoforms (Figure 3) and is located at the trimer apex near the protomer interface. We propose that its removal indirectly affects the binding of various mannose-dependent bNAbs by perturbing the glycans at the trimer apex.

To further explore some of these phenomena, we created several double-PNGS deletion mutant trimers on the BG505

SOSIP.664 N332A background, together with the corresponding single mutants. The trimers were produced as described earlier, and their glycan profiles were analyzed by HILIC-UPLC. The relative impact of single-site deletions on the abundance of oligomannose-type glycans was generally significantly less than when two sites were deleted (Figure 6; Figure S3). However, it is notable that the overall reduction in oligomannose content transcends what would be predicted solely from the engineered loss of the individual glycans. As noted earlier, the N332 site has a significant influence on the wider composition of the glycan shield on BG505 SOSIP.664 trimers. This observation is reinforced by studies using mannose-patch binding bNAbs and mutant viruses (Figure 5). Specifically, the BG505.T332 virus (which lacks the N332 glycan) is more affected by the deletion of additional glycans, compared to BG505.T332N (which contains it). Thus, unexpectedly, the glycan epitopes for PGT128 and PGT130 are particularly strongly impaired when the N448 glycan is deleted from the BG505.T332 virus, more so than seen with BG505.T332N. Of note is that, in the SOSIP.664 trimer context, the double deletion of N332 and N448 has quite a strong impact on the overall abundance of oligomannose, as does the dual elimination of N332 and N262 (Figure 6). We noted that deleting the N262 glycan also reduces the sensitivity of the BG505.T332 virus to neutralization by PGT128. However, the impact of removing the N262 glycan may be explained by its critical role in gp120 folding (Mathys et al., 2014; Moore et al., 1994) and in influencing the conformation of the N301 glycan (Kong et al., 2015).

Conversely, and unexpectedly, certain bNAbs to the mannose-patch epitopes more potently neutralize BG505 virus mutants that lack some outer domain glycans. Deleting the N137 glycan renders both BG505 test viruses more sensitive to PGT128 and PGT130 and makes BG505.T332N more vulnerable to PGT121 and PGT135, but the corresponding change has no meaningful effect on oligomannose content in the SOSIP.664 trimer context (Figure 6). The likely explanation is that the loss of the N137 glycan increases the overall accessibility of the mannose-patch epitopes but without affecting the average composition of the glycan shield (Doores et al., 2015; Garces et al., 2015).

The CD4bs antibody PGV04 more strongly neutralized the N197 and N301 single-deletion mutants of the BG505.T332 and BG505.T332N viruses. This outcome is consistent with, and extends, a recent report that deleting the N197 glycan from a diverse panel of HIV-1 isolates generally increases their sensitivity to CD4bs bNAbs, presumably via increased epitope accessibility (Townsley et al., 2015).

Glycan-Dependent Epitope of the Trimer Apex

The PG9 epitope is critically dependent on the trimer apex glycans N156 and N160 (Amin et al., 2013; Julien et al., 2013a; Walker et al., 2009). On the BG505 SOSIP.664 trimer, the N156 site is exclusively occupied by oligomannose-type glycans, mainly $Man_9GlcNAc_2$ (Figure 3). This observation is in marked contrast with a report that sialylated complex structures play a critical role in how PG9 recognizes the N156 glycan (Amin et al., 2013) and also with the proposal that sialylated hybrid glycans are targeted by the related PG16 antibody (Pancera et al., 2013). These studies used a scaffolded V1/V2 region of the trimer, and we suggest that, although antibodies may be able to bind such fragments, their glycans may not be close mimics of the native setting. A related observation is that, in the trimer context, N160 can now be seen to be occupied by a mixed array of glycans, in which $Man_8GlcNAc_2$ predominates. This information supersedes the suggestion, based on simpler structures, that processed $Man_5GlcNAc_2$ moieties play a critical role in how PG9 recognizes the N160 glycan (Amin et al., 2013). It is unclear how the presence of kifunensine-induced $Man_9GlcNAc_2$ moieties impedes viral neutralization by PG9 and PG16 (Doores and Burton, 2010). One explanation is that PG9 and PG16 may simply not bind $Man_9GlcNAc_2$ structures very well. However, an alternative hypothesis is that driving all three N160 glycans to the larger structure (i.e., by the use of kifunensine) distorts the packing of the trimer apex, with implications for the presentation of the PG9 epitope. Overall, we now show that the PG9 epitope, in the trimer context, is substantially more oligomannose tolerant/dependent than has been thought. Our findings are concordant with the observation that PG9 binds comparably to BG505 SOSIP.664 trimers regardless of whether they are produced in HEK293T cells (which permit glycan processing) or GnTI-deficient HEK293S cells (which restrict glycans to oligomannose forms) (Julien et al., 2013a).

The knowledge we present on the site-specific glycan composition of bNAb epitopes should inform us about the types of immunological response that Env vaccine candidates may need to induce. As additional native-like recombinant trimers become available, including ones specifically engineered to better present epitopes for glycan-influenced bNAbs or their germline precursors, site-specific glycan analysis will become a critical tool that will, in turn, generate yet more information to guide further design improvements. In the context of HIV-1 infection, we also propose that the dense network of glycans that covers the Env trimer will tend to promote the presence of particular carbohydrate structures, despite the underlying and much greater diversity at the amino-acid-sequence level.

EXPERIMENTAL PROCEDURES

A more detailed description of the experimental procedures is provided in the Supplemental Experimental Procedures.

Overview of BG505 Constructs

BG505 SOSIP.664 trimers used for site-specific MS analysis were stably expressed in HEK293T cells as previously described (Chung et al., 2014). The effects of deleting individual glycan sites were studied by making specific mutants of His-tagged BG505 SOSIP.664 trimers (Sanders et al., 2013), which were transiently expressed in HEK293F cells. Neutralization assays were conducted with Env-pseudoviruses produced in HEK293T cells. Viruses based on the full-length wild-type BG505 Env (termed here BG505.T332), or the BG505.T332N point mutant in which the N332 glycan site was restored, served as the basis for introducing specific mutations to delete one or two PNGSs. Mutations were made using the QuikChange Mutagenesis (Agilent Technologies) system according to the manufacturer's instructions. The primers used and the mutants created are listed in Tables S7 and S8, respectively.

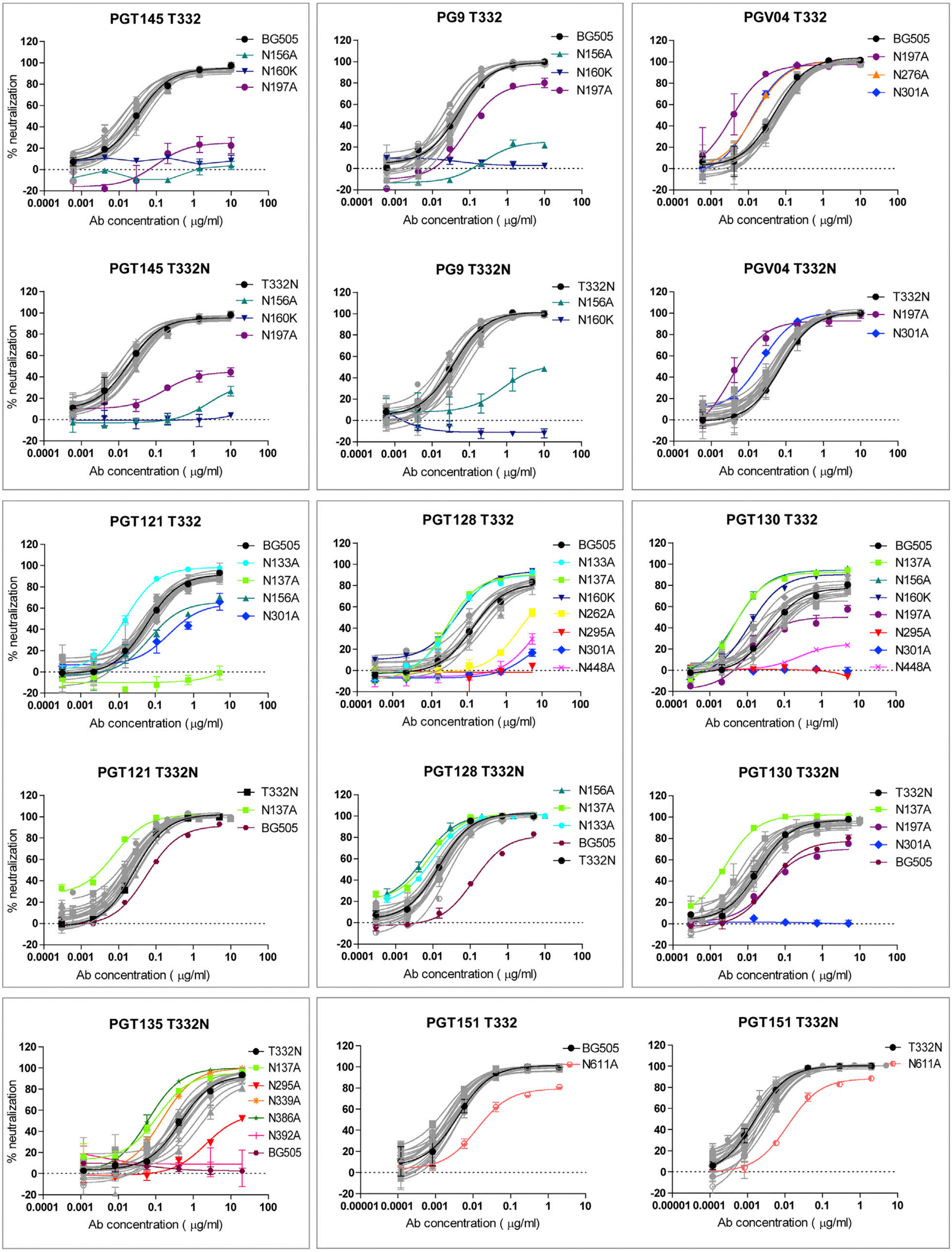

(legend on next page)

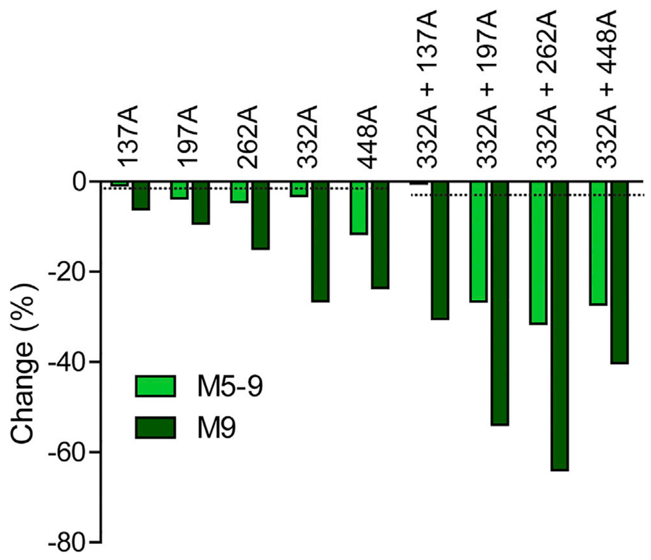

Figure 6. Impact of Individual Glycan Sites on Total Trimer Glycosylation

The figure shows the effects of various single- and double-glycan site deletions on the overall abundance of oligomannose-type glycans (M5-9; green), as well as on the individual abundance of $Man_9GlcNAc_2$ (M9; dark green). His-tagged BG505 SOSIP.664 trimers were transiently expressed in HEK293F cells and purified by PGT151-affinity chromatography. Bands corresponding to the trimeric protein were excised from native gels and subjected to glycan analysis. The abundance of oligomannose-type glycans were determined by the integration of corresponding HILIC-UPLC peaks with or without Endo H treatment. The y axis represents the change in percentage of oligomannose-type glycans of a mutant relative to the non-mutated BG505 SOSIP.664 trimer control and is calculated as follows: percent change = [(% oligomannose in control − % oligomannose in mutant)/(% oligomannose in control)] × 100. The two dashed lines represent the decrease in oligomannose abundance that would be predicted to arise solely by the deletion of one or two N-glycosylation sites that are exclusively occupied by oligomannose-type glycans when present on the control trimer.

See also Figure S3.

Expression and Purification of Env Trimers

BG505 SOSIP.664 trimers used for MS analysis of site-specific N-glycosylation were expressed in stable Flp-In HEK293T cells and purified by 2G12-affinity chromatography followed by size exclusion chromatography (SEC), as previously described (Chung et al., 2014). His-tagged BG505 SOSIP.664 trimers and corresponding PNGS mutants (discussed earlier) were transiently expressed in HEK293F cells, as described elsewhere (Sanders et al., 2013). The trimers were then purified from cell culture supernatants by PGT151-affinity chromatography, as described elsewhere (Pritchard et al., 2015d; Ringe et al., 2015).

Env-Pseudovirus Production and Neutralization Assays

To produce Env-pseudoviruses capable of single-cycle replication, HEK293T cells were cotransfected with plasmids encoding Env and an Env-deficient genomic backbone. Supernatants containing Env-pseudoviruses were harvested 72 hr post-transfection for use in neutralization assays based on TZM-bl target cells (Montefiori, 2005).

In-Gel Release of N-Linked Glycans

N-linked glycans were enzymatically released from trimers via in-gel digestion with PNGase F. The released glycans were then either fluorescently labeled or subjected to IM-ESI MS.

Glycan Analysis by HILIC-UPLC

Released glycans were fluorescently labeled with 2-aminobenzoic acid (2-AA), as previously described (Neville et al., 2009; Pritchard et al., 2015d). Labeled glycans were analyzed using a 2.1 mm × 10 mm Acquity BEH glycan column in a Waters Acquity UPLC instrument. Endo H digestions of labeled glycans were used to measure the abundance of oligomannose-type glycans.

Tandem IM-ESI MS Analysis of Released N-Linked Glycans

The total pool of released glycans from BG505 SOSIP.664 trimers (from the stable HEK293T cell line) was analyzed using IM-ESI MS with a Waters Synapt G2Si mass spectrometer (Waters). These results were the basis for the creation of a glycan library that was then used for the subsequent site-specific N-glycosylation analyses.

Proteolytic Digestion of BG505 SOSIP.664 Trimers and Glycopeptide Enrichment

A 200- to 300-μg sample of trimer was used for in-solution proteolytic digestion using trypsin or chymotrypsin (Mass Spectrometry Grade, Promega), followed by enrichment of the digestion mixture for glycopeptides using the ProteoExtract Glycopeptide Enrichment Kit (Merck Millipore). Glycopeptides were then either directly analyzed by LC-ESI MS or fractionated by RP-HPLC and subjected to MALDI-TOF MS.

RP-HPLC Fractionation and MALDI-TOF MS Analysis of Glycopeptides

Glycopeptides were resuspended in PBS buffer and fractionated with RP-HPLC a Jupiter C18 5-μm 250 × 4.5 mm column (300 Å, Phenomenex) and a Dionex U3000 LC system. Glycopeptide fractions were split in half. One half was directly analyzed by MALDI-TOF MS, whereas the other half was deglycosylated using PNGase F (New England Biolabs). MALDI-TOF MS was performed using an Autoflex Speed MALDI-TOF(/TOF) instrument (Bruker). MS/MS was performed on both glycopeptides and peptides to confirm peptide identity. Acquired data were processed using DataAnalysis 3 software (Bruker).

LC-ESI MS and MS/MS Analysis of Glycopeptides

Enriched glycopeptides were analyzed on a Q-Exactive Orbitrap mass spectrometer (Thermo Fisher Scientific) coupled to a Dionex Ultimate 3000 nanoLC system. MS data were acquired with XCalibur 3.0.63 (Thermo Fisher Scientific) using Top10 intense ions in 1.42-s duty cycle time. Glycopeptides were fragmented using higher energy collisional dissociation (HCD) fragmentation. Data analysis and glycopeptide identification were performed using Byonic (Version 2.7) and Byologic software (Version 2.3; Protein Metrics).

SUPPLEMENTAL INFORMATION

Supplemental Information includes Supplemental Experimental Procedures, three figures, and eight tables and can be found with this article online at http://dx.doi.org/10.1016/j.celrep.2016.02.058.

Figure 5. Glycan Site Modulation of Viral Neutralization by Glycan-Dependent bNAbs

Neutralization of wild-type BG505 (termed BG505.T332 in the main text) and BG505.T332N Env-pseudoviruses, and a panel of glycan-deletion mutant Env-pseudoviruses, by bNAbs PGT145, PG9, PGV04, PGT121, PGT128, PGT130, PGT135, and PGT151. The presence or absence of the N332 glycan is indicated above each panel (i.e., T332 or T332N). The plots show data from one experiment that is representative of at least two. The error bars are based on the mean values derived from two duplicate wells ± SEM. The glycan-deletion mutants based on the BG505.T332 virus were not analyzed for their sensitivity to PGT135, as this antibody (Ab) is highly N332 dependent, and the parental virus already lacks this glycan (Kong et al., 2013). Mutant Env-pseudoviruses with markedly increased or decreased neutralization sensitivity compared to the remaining panel are highlighted using the colors shown in the legends. The full list of PNGS deletions is in Table S8.

AUTHOR CONTRIBUTIONS

A.-J.B., S.V., L.K.P., D.J.H., R.S.A., S.A.K., A.C., A.K., J.H.L., P.J.K., and G.E.S. performed experimental work. A.-J.B., A.B.W., L.K.P., R.W.S., D.J.H., W.B.S., H.B.K., D.I.R.S., L.R., K.J.D., D.R.B., N.Z, J.H.L., P.J.K., and M.C. analyzed data. A.-J.B., I.A.W., A.B.W., R.W.S., W.B.S., J.P.M., K.J.D., and M.C. wrote the paper. D.R.B., I.A.W., J.P.M., K.J.D., and M.C. designed the study. All authors read and approved the final manuscript.

ACKNOWLEDGMENTS

We thank Professor Raymond A. Dwek, FRS, for insightful discussions. A.-J.B. is a recipient of a Chris Scanlan Memorial Scholarship from Corpus Christi College, University of Oxford. L.K.P. was supported by a scholarship from the Department of Biochemistry, University of Oxford. M.C. is a Fellow of Oriel College, University of Oxford. This work was supported by NIH grants P01 AI110657 (HIVRAD) and R37 AI36082, an International AIDS Vaccine Initiative Neutralizing Antibody Center CAVD grant (Glycan characterization and Outer Domain glycoform design), the Scripps CHAVI-ID (1UM1AI100663), and the Medical Research Council MR/K024426/1. R.W.S. is a recipient of a Vidi grant from the Netherlands Organization for Scientific Research (NWO) and a Starting Investigator Grant from the European Research Council (ERC-StG-2011–280829-SHEV). We are grateful to Anila Yasmeen for expert technical assistance with surface plasmon resonance analysis and Florian Klein for providing the 3BNC117 Fab plasmid. D.I.R.S. and L.R. are employees of Ludger, Ltd.

Received: December 15, 2015
Revised: January 29, 2016
Accepted: February 10, 2016
Published: March 10, 2016

REFERENCES

Amin, M.N., McLellan, J.S., Huang, W., Orwenyo, J., Burton, D.R., Koff, W.C., Kwong, P.D., and Wang, L.-X. (2013). Synthetic glycopeptides reveal the glycan specificity of HIV-neutralizing antibodies. Nat. Chem. Biol. *9*, 521–526.

Binley, J.M., Sanders, R.W., Clas, B., Schuelke, N., Master, A., Guo, Y., Kajumo, F., Anselma, D.J., Maddon, P.J., Olson, W.C., and Moore, J.P. (2000). A recombinant human immunodeficiency virus type 1 envelope glycoprotein complex stabilized by an intermolecular disulfide bond between the gp120 and gp41 subunits is an antigenic mimic of the trimeric virion-associated structure. J. Virol. *74*, 627–643.

Blattner, C., Lee, J.H., Sliepen, K., Derking, R., Falkowska, E., de la Peña, A.T., Cupo, A., Julien, J.P., van Gils, M., Lee, P.S., et al. (2014). Structural delineation of a quaternary, cleavage-dependent epitope at the gp41-gp120 interface on intact HIV-1 Env trimers. Immunity *40*, 669–680.

Bonomelli, C., Doores, K.J., Dunlop, D.C., Thaney, V., Dwek, R.A., Burton, D.R., Crispin, M., and Scanlan, C.N. (2011). The glycan shield of HIV is predominantly oligomannose independently of production system or viral clade. PLoS ONE *6*, e23521.

Calarese, D.A., Scanlan, C.N., Zwick, M.B., Deechongkit, S., Mimura, Y., Kunert, R., Zhu, P., Wormald, M.R., Stanfield, R.L., Roux, K.H., et al. (2003). Antibody domain exchange is an immunological solution to carbohydrate cluster recognition. Science *300*, 2065–2071.

Chung, N.P.Y., Matthews, K., Kim, H.J., Ketas, T.J., Golabek, M., de Los Reyes, K., Korzun, J., Yasmeen, A., Sanders, R.W., Klasse, P.J., et al. (2014). Stable 293 T and CHO cell lines expressing cleaved, stable HIV-1 envelope glycoprotein trimers for structural and vaccine studies. Retrovirology *11*, 33.

Coutu, M., and Finzi, A. (2015). HIV-1 gp120 dimers decrease the overall affinity of gp120 preparations for CD4-induced ligands. J. Virol. Methods *215-216*, 37–44.

Crispin, M., and Doores, K.J. (2015). Targeting host-derived glycans on enveloped viruses for antibody-based vaccine design. Curr. Opin. Virol. *11*, 63–69.

Depetris, R.S., Julien, J.P., Khayat, R., Lee, J.H., Pejchal, R., Katpally, U., Cocco, N., Kachare, M., Massi, E., David, K.B., et al. (2012). Partial enzymatic deglycosylation preserves the structure of cleaved recombinant HIV-1 envelope glycoprotein trimers. J. Biol. Chem. *287*, 24239–24254.

Doores, K.J. (2015). The HIV glycan shield as a target for broadly neutralizing antibodies. FEBS J. *282*, 4679–4691.

Doores, K.J., and Burton, D.R. (2010). Variable loop glycan dependency of the broad and potent HIV-1-neutralizing antibodies PG9 and PG16. J. Virol. *84*, 10510–10521.

Doores, K.J., Bonomelli, C., Harvey, D.J., Vasiljevic, S., Dwek, R.A., Burton, D.R., Crispin, M., and Scanlan, C.N. (2010). Envelope glycans of immunodeficiency virions are almost entirely oligomannose antigens. Proc. Natl. Acad. Sci. USA *107*, 13800–13805.

Doores, K.J., Kong, L., Krumm, S.A., Le, K.M., Sok, D., Laserson, U., Garces, F., Poignard, P., Wilson, I.A., and Burton, D.R. (2015). Two classes of broadly neutralizing antibodies within a single lineage directed to the high-mannose patch of HIV envelope. J. Virol. *89*, 1105–1118.

Falkowska, E., Le, K.M., Ramos, A., Doores, K.J., Lee, J.H., Blattner, C., Ramirez, A., Derking, R., van Gils, M.J., Liang, C.-H., et al. (2014). Broadly neutralizing HIV antibodies define a glycan-dependent epitope on the prefusion conformation of gp41 on cleaved envelope trimers. Immunity *40*, 657–668.

Garces, F., Sok, D., Kong, L., McBride, R., Kim, H.J., Saye-Francisco, K.F., Julien, J.-P., Hua, Y., Cupo, A., Moore, J.P., et al. (2014). Structural evolution of glycan recognition by a family of potent HIV antibodies. Cell *159*, 69–79.

Garces, F., Lee, J.H., de Val, N., Torrents de la Pena, A., Kong, L., Puchades, C., Hua, Y., Stanfield, R.L., Burton, D.R., Moore, J.P., et al. (2015). Affinity maturation of a potent family of HIV antibodies is primarily focused on accommodating or avoiding glycans. Immunity *43*, 1053–1063.

Go, E.P., Hewawasam, G., Liao, H.-X., Chen, H., Ping, L.-H., Anderson, J.A., Hua, D.C., Haynes, B.F., and Desaire, H. (2011). Characterization of glycosylation profiles of HIV-1 transmitted/founder envelopes by mass spectrometry. J. Virol. *85*, 8270–8284.

Go, E.P., Liao, H.-X., Alam, S.M., Hua, D., Haynes, B.F., and Desaire, H. (2013). Characterization of host-cell line specific glycosylation profiles of early transmitted/founder HIV-1 gp120 envelope proteins. J. Proteome Res. *12*, 1223–1234.

Go, E.P., Herschhorn, A., Gu, C., Castillo-Menendez, L., Zhang, S., Mao, Y., Chen, H., Ding, H., Wakefield, J.K., Hua, D., et al. (2015). Comparative analysis of the glycosylation profiles of membrane-anchored HIV-1 envelope glycoprotein trimers and soluble gp140. J. Virol. *89*, 8245–8257.

Guttman, M., Garcia, N.K., Cupo, A., Matsui, T., Julien, J.P., Sanders, R.W., Wilson, I.A., Moore, J.P., and Lee, K.K. (2014). CD4-induced activation in a soluble HIV-1 Env trimer. Structure *22*, 974–984.

Harvey, D.J., Sobott, F., Crispin, M., Wrobel, A., Bonomelli, C., Vasiljevic, S., Scanlan, C.N., Scarff, C.A., Thalassinos, K., and Scrivens, J.H. (2011). Ion mobility mass spectrometry for extracting spectra of N-glycans directly from incubation mixtures following glycan release: application to glycans from engineered glycoforms of intact, folded HIV gp120. J. Am. Soc. Mass Spectrom. *22*, 568–581.

Hessell, A.J., Rakasz, E.G., Tehrani, D.M., Huber, M., Weisgrau, K.L., Landucci, G., Forthal, D.N., Koff, W.C., Poignard, P., Watkins, D.I., and Burton, D.R. (2010). Broadly neutralizing monoclonal antibodies 2F5 and 4E10 directed against the human immunodeficiency virus type 1 gp41 membrane-proximal external region protect against mucosal challenge by simian-human immunodeficiency virus SHIVBa-L. J. Virol. *84*, 1302–1313.

Huang, J., Kang, B.H., Pancera, M., Lee, J.H., Tong, T., Feng, Y., Imamichi, H., Georgiev, I.S., Chuang, G.Y., Druz, A., et al. (2014). Broad and potent HIV-1 neutralization by a human antibody that binds the gp41-gp120 interface. Nature *515*, 138–142.

Julien, J.-P., Lee, J.H., Cupo, A., Murin, C.D., Derking, R., Hoffenberg, S., Caulfield, M.J., King, C.R., Marozsan, A.J., Klasse, P.J., et al. (2013a). Asymmetric recognition of the HIV-1 trimer by broadly neutralizing antibody PG9. Proc. Natl. Acad. Sci. USA *110*, 4351–4356.

Julien, J.P., Cupo, A., Sok, D., Stanfield, R.L., Lyumkis, D., Deller, M.C., Klasse, P.J., Burton, D.R., Sanders, R.W., Moore, J.P., et al. (2013b). Crystal structure of a soluble cleaved HIV-1 envelope trimer. Science *342*, 1477–1483.

Khayat, R., Lee, J.H., Julien, J.-P., Cupo, A., Klasse, P.J., Sanders, R.W., Moore, J.P., Wilson, I.A., and Ward, A.B. (2013). Structural characterization of cleaved, soluble HIV-1 envelope glycoprotein trimers. J. Virol. *87*, 9865–9872.

Klasse, P.J., Depetris, R.S., Pejchal, R., Julien, J.-P., Khayat, R., Lee, J.H., Marozsan, A.J., Cupo, A., Cocco, N., Korzun, J., et al. (2013). Influences on trimerization and aggregation of soluble, cleaved HIV-1 SOSIP envelope glycoprotein. J. Virol. *87*, 9873–9885.

Kong, L., Sheppard, N.C., Stewart-Jones, G.B., Robson, C.L., Chen, H., Xu, X., Krashias, G., Bonomelli, C., Scanlan, C.N., Kwong, P.D., et al. (2010). Expression-system-dependent modulation of HIV-1 envelope glycoprotein antigenicity and immunogenicity. J. Mol. Biol. *403*, 131–147.

Kong, L., Lee, J.H., Doores, K.J., Murin, C.D., Julien, J.P., McBride, R., Liu, Y., Marozsan, A., Cupo, A., Klasse, P.J., et al. (2013). Supersite of immune vulnerability on the glycosylated face of HIV-1 envelope glycoprotein gp120. Nat. Struct. Mol. Biol. *20*, 796–803.

Kong, L., Torrents de la Peña, A., Deller, M.C., Garces, F., Sliepen, K., Hua, Y., Stanfield, R.L., Sanders, R.W., and Wilson, I.A. (2015). Complete epitopes for vaccine design derived from a crystal structure of the broadly neutralizing antibodies PGT128 and 8ANC195 in complex with an HIV-1 Env trimer. Acta Crystallogr. D Biol. Crystallogr. *71*, 2099–2108.

Kovacs, J.M., Noeldeke, E., Ha, H.J., Peng, H., Rits-Volloch, S., Harrison, S.C., and Chen, B. (2014). Stable, uncleaved HIV-1 envelope glycoprotein gp140 forms a tightly folded trimer with a native-like structure. Proc. Natl. Acad. Sci. USA *111*, 18542–18547.

Lasky, L.A., Groopman, J.E., Fennie, C.W., Benz, P.M., Capon, D.J., Dowbenko, D.J., Nakamura, G.R., Nunes, W.M., Renz, M.E., and Berman, P.W. (1986). Neutralization of the AIDS retrovirus by antibodies to a recombinant envelope glycoprotein. Science *233*, 209–212.

Lee, J.H., de Val, N., Lyumkis, D., and Ward, A.B. (2015). Model building and refinement of a natively glycosylated HIV-1 Env protein by high-resolution cryoelectron microscopy. Structure *23*, 1943–1951.

Leonard, C.K., Spellman, M.W., Riddle, L., Harris, R.J., Thomas, J.N., and Gregory, T.J. (1990). Assignment of intrachain disulfide bonds and characterization of potential glycosylation sites of the type 1 recombinant human immunodeficiency virus envelope glycoprotein (gp120) expressed in Chinese hamster ovary cells. J. Biol. Chem. *265*, 10373–10382.

Lyumkis, D., Julien, J.P., de Val, N., Cupo, A., Potter, C.S., Klasse, P.J., Burton, D.R., Sanders, R.W., Moore, J.P., Carragher, B., et al. (2013). Cryo-EM structure of a fully glycosylated soluble cleaved HIV-1 envelope trimer. Science *342*, 1484–1490.

Mascola, J.R., Stiegler, G., VanCott, T.C., Katinger, H., Carpenter, C.B., Hanson, C.E., Beary, H., Hayes, D., Frankel, S.S., Birx, D.L., and Lewis, M.G. (2000). Protection of macaques against vaginal transmission of a pathogenic HIV-1/SIV chimeric virus by passive infusion of neutralizing antibodies. Nat. Med. *6*, 207–210.

Mathys, L., François, K.O., Quandte, M., Braakman, I., and Balzarini, J. (2014). Deletion of the highly conserved N-glycan at Asn260 of HIV-1 gp120 affects folding and lysosomal degradation of gp120, and results in loss of viral infectivity. PLoS ONE *9*, e101181.

McLellan, J.S., Pancera, M., Carrico, C., Gorman, J., Julien, J.-P., Khayat, R., Louder, R., Pejchal, R., Sastry, M., Dai, K., et al. (2011). Structure of HIV-1 gp120 V1/V2 domain with broadly neutralizing antibody PG9. Nature *480*, 336–343.

Moldt, B., Rakasz, E.G., Schultz, N., Chan-Hui, P.Y., Swiderek, K., Weisgrau, K.L., Piaskowski, S.M., Bergman, Z., Watkins, D.I., Poignard, P., and Burton, D.R. (2012). Highly potent HIV-specific antibody neutralization in vitro translates into effective protection against mucosal SHIV challenge in vivo. Proc. Natl. Acad. Sci. USA *109*, 18921–18925.

Montefiori, D.C. (2005). Evaluating neutralizing antibodies against HIV, SIV, and SHIV in luciferase reporter gene assays. Curr. Protoc. Immunol. *Chapter 12*, 11.

Moore, J.P., Willey, R.L., Lewis, G.K., Robinson, J., and Sodroski, J. (1994). Immunological evidence for interactions between the first, second, and fifth conserved domains of the gp120 surface glycoprotein of human immunodeficiency virus type 1. J. Virol. *68*, 6836–6847.

Mouquet, H., Scharf, L., Euler, Z., Liu, Y., Eden, C., Scheid, J.F., Halper-Stromberg, A., Gnanapragasam, P.N.P., Spencer, D.I.R., Seaman, M.S., et al. (2012). Complex-type N-glycan recognition by potent broadly neutralizing HIV antibodies. Proc. Natl. Acad. Sci. USA *109*, E3268–E3277.

Neville, D.C., Dwek, R.A., and Butters, T.D. (2009). Development of a single column method for the separation of lipid- and protein-derived oligosaccharides. J. Proteome Res. *8*, 681–687.

Pabst, M., Chang, M., Stadlmann, J., and Altmann, F. (2012). Glycan profiles of the 27 N-glycosylation sites of the HIV envelope protein CN54gp140. Biol. Chem. *393*, 719–730.

Pancera, M., Shahzad-Ul-Hussan, S., Doria-Rose, N.A., McLellan, J.S., Bailer, R.T., Dai, K., Loesgen, S., Louder, M.K., Staupe, R.P., Yang, Y., et al. (2013). Structural basis for diverse N-glycan recognition by HIV-1-neutralizing V1-V2-directed antibody PG16. Nat. Struct. Mol. Biol. *20*, 804–813.

Pejchal, R., Doores, K.J., Walker, L.M., Khayat, R., Huang, P.-S., Wang, S.-K., Stanfield, R.L., Julien, J.-P., Ramos, A., Crispin, M., et al. (2011). A potent and broad neutralizing antibody recognizes and penetrates the HIV glycan shield. Science *334*, 1097–1103.

Pritchard, L.K., Harvey, D.J., Bonomelli, C., Crispin, M., and Doores, K.J. (2015a). Cell- and protein-directed glycosylation of native cleaved HIV-1 envelope. J. Virol. *89*, 8932–8944.

Pritchard, L.K., Spencer, D.I., Royle, L., Bonomelli, C., Seabright, G.E., Behrens, A.J., Kulp, D.W., Menis, S., Krumm, S.A., Dunlop, D.C., et al. (2015b). Glycan clustering stabilizes the mannose patch of HIV-1 and preserves vulnerability to broadly neutralizing antibodies. Nat. Commun. *6*, 7479.

Pritchard, L.K., Spencer, D.I., Royle, L., Vasiljevic, S., Krumm, S.A., Doores, K.J., and Crispin, M. (2015c). Glycan microheterogeneity at the PGT135 antibody recognition site on HIV-1 gp120 reveals a molecular mechanism for neutralization resistance. J. Virol. *89*, 6952–6959.

Pritchard, L.K., Vasiljevic, S., Ozorowski, G., Seabright, G.E., Cupo, A., Ringe, R., Kim, H.J., Sanders, R.W., Doores, K.J., Burton, D.R., et al. (2015d). Structural constraints determine the glycosylation of HIV-1 envelope trimers. Cell Rep. *11*, 1604–1613.

Ringe, R.P., Sanders, R.W., Yasmeen, A., Kim, H.J., Lee, J.H., Cupo, A., Korzun, J., Derking, R., van Montfort, T., Julien, J.-P., et al. (2013). Cleavage strongly influences whether soluble HIV-1 envelope glycoprotein trimers adopt a native-like conformation. Proc. Natl. Acad. Sci. USA *110*, 18256–18261.

Ringe, R.P., Yasmeen, A., Ozorowski, G., Go, E.P., Pritchard, L.K., Guttman, M., Ketas, T.A., Cottrell, C.A., Wilson, I.A., Sanders, R.W., et al. (2015). Influences on the design and purification of soluble, recombinant native-like HIV-1 envelope glycoprotein trimers. J. Virol. *89*, 12189–12210.

Sanders, R.W., Vesanen, M., Schuelke, N., Master, A., Schiffner, L., Kalyanaraman, R., Paluch, M., Berkhout, B., Maddon, P.J., Olson, W.C., et al. (2002). Stabilization of the soluble, cleaved, trimeric form of the envelope glycoprotein complex of human immunodeficiency virus type 1. J. Virol. *76*, 8875–8889.

Sanders, R.W., Derking, R., Cupo, A., Julien, J.P., Yasmeen, A., de Val, N., Kim, H.J., Blattner, C., de la Peña, A.T., Korzun, J., et al. (2013). A next-generation cleaved, soluble HIV-1 Env trimer, BG505 SOSIP.664 gp140, expresses multiple epitopes for broadly neutralizing but not non-neutralizing antibodies. PLoS Pathog. *9*, e1003618.

Sanders, R.W., van Gils, M.J., Derking, R., Sok, D., Ketas, T.J., Burger, J.A., Ozorowski, G., Cupo, A., Simonich, C., Goo, L., et al. (2015). HIV-1 VACCINES. HIV-1 neutralizing antibodies induced by native-like envelope trimers. Science *349*, aac4223.

Scanlan, C.N., Pantophlet, R., Wormald, M.R., Ollmann Saphire, E., Stanfield, R., Wilson, I.A., Katinger, H., Dwek, R.A., Rudd, P.M., and Burton, D.R. (2002). The broadly neutralizing anti-human immunodeficiency virus type 1 antibody 2G12 recognizes a cluster of alpha1–>2 mannose residues on the outer face of gp120. J. Virol. *76*, 7306–7321.

Scharf, L., Scheid, J.F., Lee, J.H., West, A.P., Jr., Chen, C., Gao, H., Gnanapragasam, P.N.P., Mares, R., Seaman, M.S., Ward, A.B., et al. (2014). Antibody 8ANC195 reveals a site of broad vulnerability on the HIV-1 envelope spike. Cell Rep. *7*, 785–795.

Sok, D., Doores, K.J., Briney, B., Le, K.M., Saye-Francisco, K.L., Ramos, A., Kulp, D.W., Julien, J.P., Menis, S., Wickramasinghe, L., et al. (2014). Promiscuous glycan site recognition by antibodies to the high-mannose patch of gp120 broadens neutralization of HIV. Sci. Transl. Med. *6*, 236ra63.

Townsley, S., Li, Y., Kozyrev, Y., Cleveland, B., and Hu, S.L. (2015). Conserved role of an N-linked glycan on the surface antigen of human immunodeficiency virus type 1 modulating virus sensitivity to broadly neutralizing antibodies against the receptor and coreceptor binding sites. J. Virol. *90*, 829–841.

Wada, Y. (2013). Glycan profiling: label-free analysis of glycoproteins. Methods Mol. Biol. *951*, 245–253.

Walker, L.M., Phogat, S.K., Chan-Hui, P.-Y., Wagner, D., Phung, P., Goss, J.L., Wrin, T., Simek, M.D., Fling, S., Mitcham, J.L., et al.; Protocol G Principal Investigators (2009). Broad and potent neutralizing antibodies from an African donor reveal a new HIV-1 vaccine target. Science *326*, 285–289.

Walker, L.M., Huber, M., Doores, K.J., Falkowska, E., Pejchal, R., Julien, J.-P., Wang, S.-K., Ramos, A., Chan-Hui, P.-Y., Moyle, M., et al.; Protocol G Principal Investigators (2011). Broad neutralization coverage of HIV by multiple highly potent antibodies. Nature *477*, 466–470.

Wei, X., Decker, J.M., Wang, S., Hui, H., Kappes, J.C., Wu, X., Salazar-Gonzalez, J.F., Salazar, M.G., Kilby, J.M., Saag, M.S., et al. (2003). Antibody neutralization and escape by HIV-1. Nature *422*, 307–312.

Woods, R.J., Pathiaseril, A., Wormald, M.R., Edge, C.J., and Dwek, R.A. (1998). The high degree of internal flexibility observed for an oligomannose oligosaccharide does not alter the overall topology of the molecule. Eur. J. Biochem. *258*, 372–386.

Wu, X., Parast, A.B., Richardson, B.A., Nduati, R., John-Stewart, G., Mbori-Ngacha, D., Rainwater, S.M., and Overbaugh, J. (2006). Neutralization escape variants of human immunodeficiency virus type 1 are transmitted from mother to infant. J. Virol. *80*, 835–844.

Zhu, X., Borchers, C., Bienstock, R.J., and Tomer, K.B. (2000). Mass spectrometric characterization of the glycosylation pattern of HIV-gp120 expressed in CHO cells. Biochemistry *39*, 11194–11204.

Cell Reports, Volume *14*

Supplemental Information

Composition and Antigenic Effects of Individual Glycan Sites of a Trimeric HIV-1 Envelope Glycoprotein

Anna-Janina Behrens, Snezana Vasiljevic, Laura K. Pritchard, David J. Harvey, Rajinder S. Andev, Stefanie A. Krumm, Weston B. Struwe, Albert Cupo, Abhinav Kumar, Nicole Zitzmann, Gemma E. Seabright, Holger B. Kramer, Daniel I.R. Spencer, Louise Royle, Jeong Hyun Lee, Per J. Klasse, Dennis R. Burton, Ian A. Wilson, Andrew B. Ward, Rogier W. Sanders, John P. Moore, Katie J. Doores, and Max Crispin

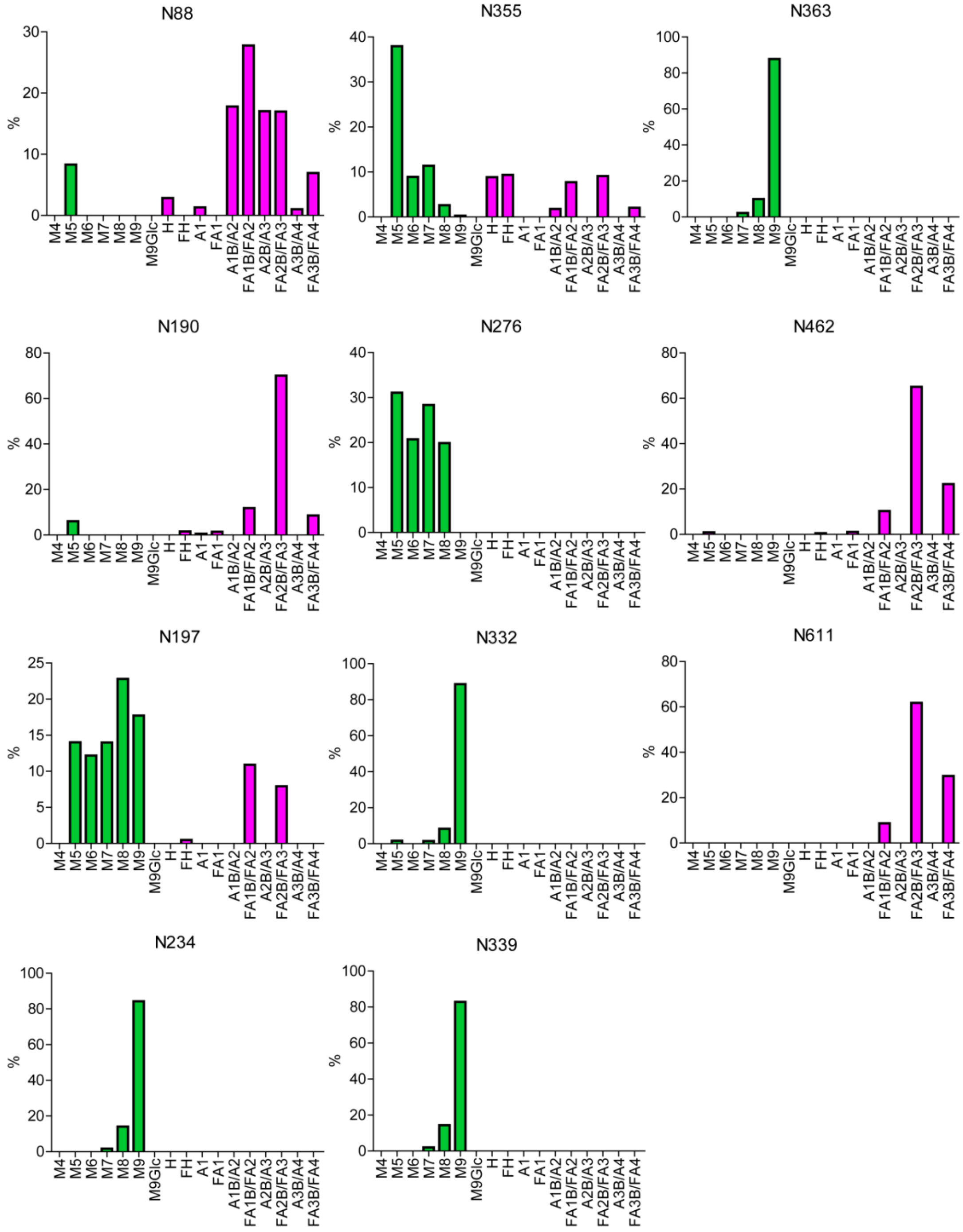

Figure S1. Relative quantification of N-glycosylation sites on BG505 SOSIP.664 trimers identified by MALDI-TOF MS, related to Figure 2. BG505 SOSIP.664 trimers, stably produced in 293T cells and purified by 2G12 affinity chromatography/SEC, were reduced, alkylated, digested with trypsin and then fractionated by RP-HPLC. Fractions were analysed by MALDI-TOF MS and MS/MS in positive mode. In cases where glycopeptides eluted over more than one fraction, the relevant fractions were pooled and reanalysed. The microheterogeneity at individual N-glycosylation sites was quantified in the following way: [Sum of areas of isotopic peaks of one glycoform] / [sum of areas of isotopic peaks of all glycoforms] x 100. Glycans were grouped as shown in Table S2; green: oligomannose-type glycans; pink: hybrid and complex glycans. The peak lists corresponding to these data including intensities can be found in Table S3 and Table S4.

A

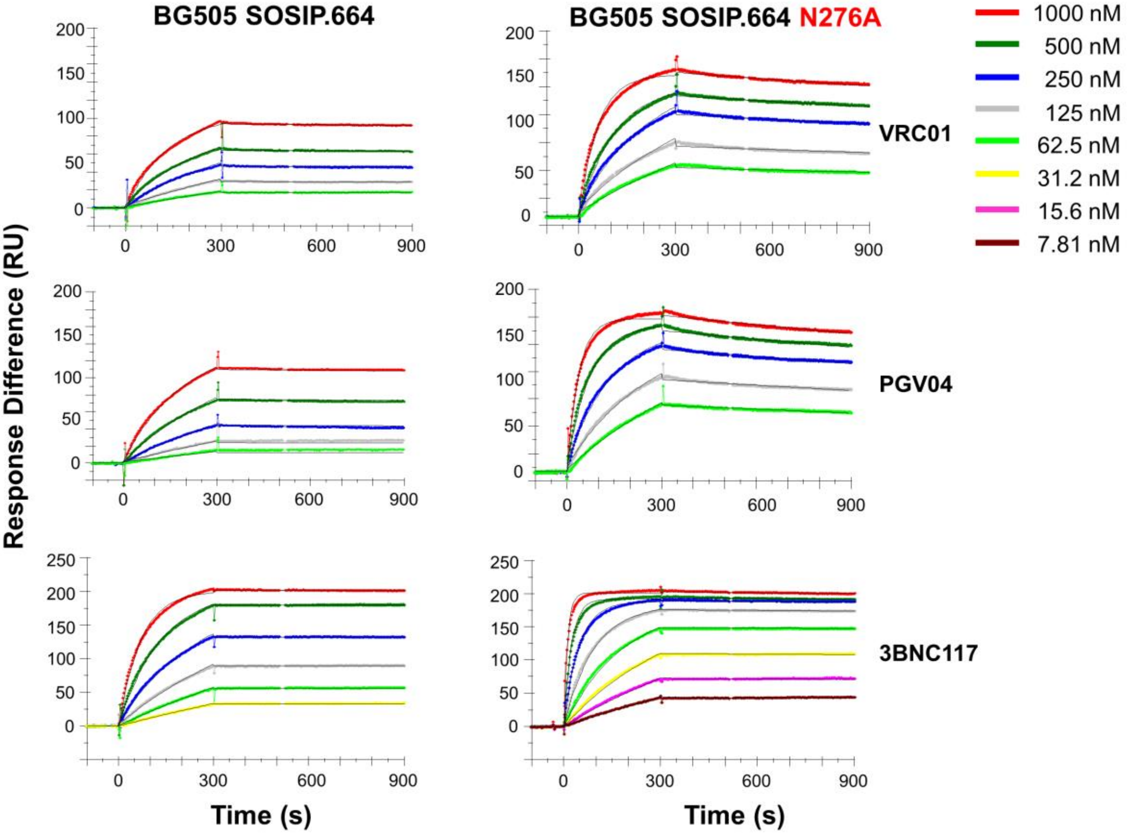

B

mAb (Fab)	BG505 SOSIP.664	k_{on} (1/Ms)	k_{off} (1/s)	K_d (nM)	S_m
**VRC01**	WT (n=2)[a]	$8.6 \cdot 10^3 \pm 1.0 \cdot 10^2$	$< 10^{-5}$ [b]	< 1 [b]	1.6 ± 0.061
	N276A (n=3)	$1.7 \cdot 10^4 \pm 3.3 \cdot 10^2$	$1.7 \cdot 10^{-4} \pm 1.0 \cdot 10^{-5}$	10 ± 0.50	2.5 ± 0.055
**PGV04**	WT (n=2)	$6.7 \cdot 10^3 \pm 50$	$3.2 \cdot 10^{-5} \pm 1.5 \cdot 10^{-6}$	4.8 ± 0.20	2.0 ± 0.034
	N276A (n=3)	$2.6 \cdot 10^4 \pm 3.3 \cdot 10^2$	$2.1 \cdot 10^{-4} \pm 8.8 \cdot 10^{-6}$	8.0 ± 0.40	2.7 ± 0.054
**3BNC117**	WT (n=3)	$1.5 \cdot 10^4 \pm 0$	$< 10^{-5}$ [b]	< 0.1 [b]	3.0 ± 0.053
	N276A (n=3)	$8.4 \cdot 10^4 \pm 1.5 \cdot 10^3$	$2.7 \cdot 10^{-5} \pm 2.9 \cdot 10^{-6}$	0.30 ± 0.040	2.9 ± 0.028

Figure S2. Binding of CD4bs-specific Fabs to BG505 SOSIP.664 and N276A mutant trimers analysed by SPR, related to Figure 3. (A) The binding of Fabs at different concentrations (legend to the right), after background subtraction, is given on the y axes (response units, RU) over the time periods shown on the x axis (s). Association was monitored for 300 s and dissociation for 600 s. Deleting the N276 glycan site increased both the on- and off-rate constants for all three Fabs. (B) Langmuir-modelled binding parameters. [a]Tabulated parameter values were obtained by fitting a Langmuir model to the binding data; the values are the means ± SEM of n independent experiments. [b]Off-rate constants below significant detectability (T<10) and the corresponding dissociation constants are given as <.

A

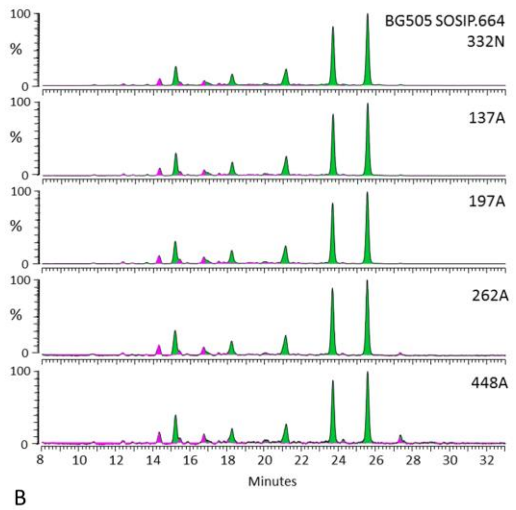

B

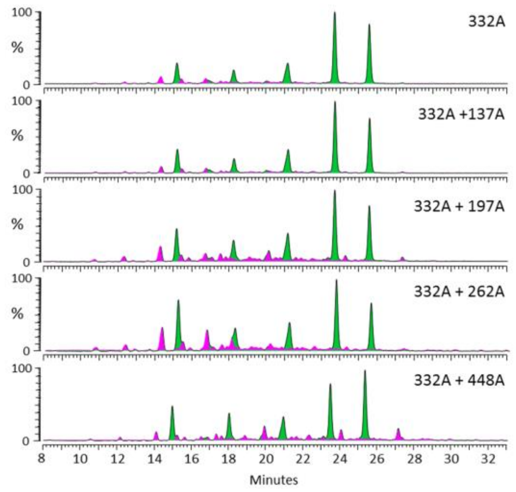

C

	Man5	Man6	Man7	Man8	Man9	sum
BG505 SOSIP.664	8	5	9	24	30	75
137A	9	5	9	24	28	75
332A	8	5	10	27	22	73
197A	9	5	8	23	27	73
262A	10	6	8	23	25	72
332A	8	5	10	27	22	73
448A	10	6	8	20	23	67
332A+137A	9	6	12	27	21	75
332A+197A	9	6	9	18	14	55
332A+262A	12	5	8	16	11	52
332A+448A	9	7	8	14	18	55

Figure S3. HILIC-UPLC profiles of a panel of PNGS-deletion mutants of BG505 SOSIP.664 trimers, related to Figure 6. A selection of single and double N-glycan site knockout mutants of the His-tagged BG505 SOSIP.664 trimer, together with the unmodified control trimer, were expressed by transient transfection of HEK 293F cells and purified by PGT151 affinity chromatography. The eluted Env proteins were resolved by BN-PAGE and the bands corresponding to trimers were excised. N-glycans were released from the gel bands using PNGase F and fluorescently labelled. Glycan profiles with and without Endo H digestion were analysed by HILIC-UPLC. Peaks corresponding to oligomannose-type and hybrid-type glycans are depicted in green, complex-type glycans in pink. (A) The panel of single PNGS knockout mutants. (B) The corresponding panel of N332A double PNGS knockout mutants. (C) Abundances (as percentages of total glycans) of oligomannose-type glycans $Man_{5\text{-}9}GlcNAc_2$ (Man5-Man9). The changes seen in the various mutants, relative to the BG505 SOSIP.664 control trimers, are presented in Figure 6.

Table S1. Library of glycan structures identified on BG505 SOSIP.664 trimers, related to Figure 1. Structures are represented as shown in Figure 1 using the Oxford glycan nomenclature (Oxford) as previously described (Harvey et al., 2011) and also the nomenclature from the Consortium of Functional Glycomics (CFG). MW: Molecular weight; Calc: Calculated.

Table S2. Classification of identified N-linked glycan compositions, related to Figure 2. Glycan names are constructed as follows: Mn = number (n) of mannose residues; An = number (n) of antennae (e.g. A3 = triantennary); Gn = number (n) of galactose residues; F in the front of the name indicates the presence of a core fucose.

Group Name	Members[a]
M4	M4
M5	M5
M6	M6
M7	M7
M8	M8
M9	M9
M9Glc	M9Glc
H/Hybrid	M4A1G1, M5A1G1, M6A1G1, M4A1G1S1, M5A1G1S1,
FH/Fucosylated-Hybrid	FM4A1G1, FM5A1G1, FM4A1G1S1, FM5A1G1S1
A1	A1, A1G1, A1G1S1
FA1	FA1, FA1G1, FA1G1S1
A1B/A2	A2, A2G1, A2G2, A2G1S1, A2G2S2
FA1B/FA2	FA2, FA2G1, FA2G2, FA2G1S1, FA2G2S1, FA2G2S2
A2B/A3	A2B, A2BG1, A2BG2, A3G3, A3G2S1, A3G3S1, A3G3S2, A3G3S3
FA2B/FA3	FA2B, FA2BG1, FA2BG2, FA3G3, FA3G3S1, FA3G3S2, FA3G3S3, FA2BG2S1
A3B/A4	A4, A4G1, A4G2, A4G3, A4G4, A4G4S1, A4G4S2, A4G4S3, A4G4S4
FA3B/FA4	FA3B, FA4G1, FA3BG1, FA3BG2, FA3BG3, FA4G4, FA4G4S1, FA4G4S2, FA4G4S3, FA4G4S4

[a]Isobaric glycans are not listed. Unusual glycan structures with terminal fucoses, sulfates or *N*-acetylgalactosamines are categorized according to the number of their antennae.

Table S3. N-linked glycopeptide compositions of trypsin-digested BG505 SOSIP.664 trimers identified by MALDI-TOF MS, related to Figure 2. Site: N-glycosylation site; Mod.: Modifications; Ad.: Adduct; C: Carbamidomethylated; PG: Pyro-Glu; O: Oxidation; Exp.: Experimental determined *m/z*; Calc.: Calculated *m/z*; Intensity: Intensity counts of all isotopic peaks. The second dataset shows N-linked glycopeptide compositions identified from pooled RP-HPLC fractions by MALDI-TOF MS.

Table S4. N-linked glycopeptide compositions of trypsin-digested BG505 SOSIP.664 trimers identified by LC-ESI MS, related to Figure 3. Site: N-glycosylation site; XIC: Extracted ion chromatogram; Exp.: Experimental determined mass (shown as a range when different charge states and/or different scans were recorded); Calc.: Calculated mass. All cysteines are carbamidomethylated. Lower case letters in sequence indicate the position of the modification. Table contains data from two analytical replicates.

Table S5. N-linked glycopeptide compositions of chymotrypsin-digested BG505 SOSIP.664 trimers identified by LC-ESI MS, related to Figure 3. Site: N-glycosylation site; XIC: Extracted ion chromatogram; Exp.: Experimental determined mass (shown as a range when different charge states and/or different scans were recorded); Calc.: Calculated mass; O: Oxidation. All cysteines are carbamidomethylated. Lower case letters in sequence indicate the position of the modification. Table contains data from two analytical replicates.

Table S6. Semi-quantification of glycopeptides identified by LC-ESI MS, related to Figure 3. The numbers are the % of the total glycan pool at any given position. Glycopeptides are grouped as shown in Table S2. T (trypsin) and C (chymotrypsin) indicate the digestion origins of the glycopeptide used for quantification. Average shows the average abundance of the different glycopeptide groups among the 20 identified N-glycosylation sites.

	**N88**	**N156**	**N160**	**N190**	**N197**	**N234**	**N262**	**N276**	**N295**	**N332**	**N339**
**M4**	0.3	0.0	0.7	0.0	0.1	0.0	1.2	0.8	0.0	0.0	2.5
**M5**	6.2	2.1	6.3	4.8	14.9	0.0	7.3	21.3	0.0	0.4	1.3
**M6**	2.1	2.4	9.4	0.7	7.5	2.6	9.4	19.8	0.0	0.0	1.5
**M7**	0.4	14.3	13.4	0.9	10.6	6.6	9.8	25.8	0.0	0.9	2.4
**M8**	0.9	21.7	28.4	0.8	12.7	17.5	17.3	20.8	18.6	9.6	17.0
**M9**	4.7	59.3	9.9	0.0	3.9	73.3	55.0	0.3	81.4	89.1	75.3
**M9Glc**	1.4	0.0	0.0	0.0	0.0	0.0	0.0	0.0	0.0	0.0	0.0
**H**	5.3	0.1	0.0	1.8	7.4	0.0	0.0	6.7	0.0	0.0	0.0
**FH**	4.3	0.0	0.3	4.9	10.5	0.0	0.0	0.0	0.0	0.0	0.0
**A1**	1.3	0.0	0.0	0.6	0.3	0.0	0.0	0.9	0.0	0.0	0.0
**FA1**	0.1	0.0	0.1	3.7	0.6	0.0	0.0	0.0	0.0	0.0	0.0
**A1B/A2**	17.9	0.1	1.5	2.4	3.0	0.0	0.0	2.5	0.0	0.0	0.0
**FA1B/FA2**	31.4	0.0	27.0	43.5	13.7	0.0	0.0	0.2	0.0	0.0	0.0
**A2B/A3**	10.5	0.0	0.0	0.0	0.2	0.0	0.0	0.7	0.0	0.0	0.0
**FA2B/FA3**	7.6	0.0	3.0	31.7	12.1	0.0	0.0	0.4	0.0	0.0	0.0
**A3B/A4**	2.4	0.0	0.0	0.0	0.0	0.0	0.0	0.0	0.0	0.0	0.0
**FA3B/FA4**	3.3	0.0	0.0	4.2	2.7	0.0	0.0	0.0	0.0	0.0	0.0
**Digestion**	T	C	T	C	T	T	C	T	T	T	T

	**N355**	**N363**	**N386**	**N392**	**N448**	**N462**	**N611**	**N618**	**N637**	**Average**
**M4**	1.7	0.0	0.0	0.0	0.1	0.0	0.0	0.0	0.0	0.4
**M5**	28.9	0.0	0.0	0.0	0.5	2.5	0.0	0.5	8.1	5.3
**M6**	10.4	6.2	0.7	1.4	1.6	0.5	0.0	0.0	11.1	4.4
**M7**	13.4	4.9	3.6	5.1	5.7	0.4	0.0	0.0	15.7	6.7
**M8**	4.3	18.7	21.0	28.8	26.2	0.2	0.0	0.0	9.7	13.7
**M9**	0.6	70.2	74.7	64.7	65.5	0.0	0.0	0.0	1.6	36.5
**M9Glc**	0.0	0.0	0.0	0.0	0.3	0.0	0.0	0.0	0.0	0.1
**H**	8.7	0.0	0.0	0.0	0.0	3.3	0.4	0.0	12.1	2.3
**FH**	12.2	0.0	0.0	0.0	0.0	2.6	12.3	9.0	14.1	3.5
**A1**	0.5	0.0	0.0	0.0	0.0	2.7	0.0	0.0	0.7	0.4
**FA1**	2.3	0.0	0.0	0.0	0.0	3.4	0.0	2.9	4.7	0.9
**A1B/A2**	2.2	0.0	0.0	0.0	0.0	1.3	3.5	0.0	0.0	1.7
**FA1B/FA2**	7.9	0.0	0.0	0.0	0.0	18.2	25.2	40.8	16.4	11.2
**A2B/A3**	0.2	0.0	0.0	0.0	0.0	0.3	0.0	0.0	0.0	0.6
**FA2B/FA3**	5.6	0.0	0.0	0.0	0.0	56.3	39.9	23.6	5.6	9.3
**A3B/A4**	0.0	0.0	0.0	0.0	0.0	0.0	0.0	0.0	0.0	0.1
**FA3B/FA4**	1.1	0.0	0.0	0.0	0.0	8.3	18.7	23.2	0.2	3.1
**Digestion**	T	T	C	C	T	T	T	C	T	

Table S7. Primers used for site-directed mutagenesis of BG505.SOSIP.664 trimers, related to Experimental Procedures.

	**Primer sequence (5'-to-3')**
BG505 SOSIP.664 137A	GTACAAACGTGACTAACGCTATCACCGACGATATGCG
BG505 SOSIP.664 197A	CAGGCTGATCAATTGCGCCACCAGTGCCATTACAC
BG505 SOSIP.664 262A	CAGCTGCTGCTGGCTGGATCTCTGGCC
BG505 SOSIP.664 332A	CAGGCACACTGTGCTGTGAGCAAGGCTAC
BG505 SOSIP.664 448A	GTGATTCGATGTGTCAGCGCTATCACAGGCCTGATTC

Table S8. Primers used for site-directed mutagenesis of full-length Env, related to Experimental Procedures.

	**Primer sequence (5'-to-3')**
N133A	CTCTACAGTGTACCGCTGTCACCAATAATATC
N137A	CCAATGTCACCAATGCTATCACTGATGACATG
N156A	GAGGGGAGAATTAAAAGCCTGCTCTTTC
N160K	AACTGCTCTTTCAAGATGACCACAGAGCTAAGG
N190A	ACGAGAATCAAGGTGCTAGGAGTAATAATAGTA
N190cA	CGAGAATCAAGGTGCTAGGAGTAATAATAGTAAC
N197A	GATTAATAAATTGTGCTACCTCAGCCATTAC
N262A	CTCAACTGCTGTTAGCTGGCAGTCTAGCAG
N276A	ATGATTAGATCTGAAGCTATCACAAACAATGC
N295A	ACGCCTGTGCAAATTGCTTGTACCAGACCTAAC
N301A	GTACCAGACCTAACGCCAATACAAGGAAAAG
T332N	GACAAGCACATTGTAATGTCAGTAAAGCAA
N339A	GTCAGTAAAGCAACATGGGCTGAAACTTTGGG
N386A	GAATTTTTCTATTGTGCCACATCAGGCCTGTTC
N392A	CATCAGGCCTGTTCGCTAGCACTTGGATTAG
N448A	AATAAGATGTGTATCAGCCATTACAGGGCTAATA
N611A	TAATGTGCCCTGGGCCTCTAGTTGGAGTAATA
N618A	TTGGAGTAATAGAGCCCTGAGTGAGATATGGG
N625A	AGTGAGATATGGGACGCTATGACCTGGCTGC
N637A	GGGATAAAGAAATTAGCGCCTACACACAGATAA

Supplemental Experimental Procedures

Overview of BG505 constructs

BG505 SOSIP.664 trimers used for site-specific MS analysis were stably expressed in HEK 293T cells as previously described (Chung et al., 2014). The effects of deleting individual glycan sites were studied by making specific mutants of His-tagged BG505 SOSIP.664 trimers (Sanders et al., 2013), which were transiently expressed in HEK 293F cells. SPR experiments were conducted with BG505 SOSIP.664 trimers transiently expressed in 293T cells. Neutralization assays were conducted with Env-pseudoviruses produced in HEK 293T cells. Viruses based on the full-length wild-type BG505 Env glycoprotein (termed here BG505.T332), or the BG505.T332N point mutant in which the N332 glycan site was restored, served as the basis for introducing specific mutations to delete one or two PNGS.

Site-directed mutagenesis

The pPPI4 vector expressing His-tagged BG505 SOSIP.664 has been previously described (Sanders et al., 2013). Single and double mutants of a selection of N-glycan sites in this construct, as well as in the BG505.T332 and BG505.T332N full-length Env proteins, were made by changing asparagine (within the N-glycosylation sequence Asn-X-Ser/Thr, where X is any amino acid except proline) to alanine or (for the N160 site, only) lysine, using the QuikChange Mutagenesis (Agilent) system according to the manufacturer's instructions. Changes were verified by DNA sequencing. The primers used and the list of mutants created are listed in Table S7 and Table S8, respectively.

Expression and purification of Env trimers

BG505 SOSIP.664 trimers used for mass spectrometry analysis of site-specific N-glycosylation were expressed in stable Flp-In HEK 293T cells and purified by 2G12-affinity chromatography followed by SEC, as previously described (Chung et al., 2014). His-tagged BG505 SOSIP.664 trimers and PNGS mutants thereof (see above) were transiently expressed in HEK 293F cells, as described elsewhere (Sanders et al., 2013). The trimers were then purified from cell culture supernatants by PGT151

affinity chromatography as described elsewhere (Pritchard et al., 2015; Ringe et al., 2015). Eluted trimers were immediately buffer exchanged into 10 mM Tris-HCl, 75 mM NaCl (pH 8.0) and concentrated using a 50 kDa cut-off Vivaspin column (GE Healthcare).

Env-pseudovirus production and neutralization assays

To produce Env-pseudoviruses capable of single-cycle replication, HEK 293T cells were co-transfected (PEI, 1 mg/mL, 1:3 PEI:total DNA, Polysciences) with plasmids encoding Env and an Env-deficient genomic backbone (pSG3ΔEnv, 1:2 ratio). Supernatants containing Env-pseudoviruses were harvested 72 h post transfection for use in neutralization assays based on TZM-bl target cells (Li et al., 2005; Montefiori, 2005). Briefly, a serial diluted antibody was pre-incubated with virus for 1 h at 37 °C in a 96-well, flat bottom plate. TZM-bl cells (20,000 cells/well) were added to the virus/antibody mixture and incubated for 72 h. The cells were lysed and luminescence was quantified via addition of Bright-Glo™ Luciferase substrate (Promega). To determine IC_{50} values, dose-response curves were fitted using nonlinear regression (GraphPad Prism).

In-gel release of N-linked glycans

N-linked glycans were enzymatically released from trimers via in-gel digestion. Purified trimers were fractionated by reducing SDS-PAGE (addition of DL-Dithiothreitol; DTT) or by BN-PAGE and stained with Coomassie Blue. Following destaining, the bands corresponding to the trimeric protein (BN-PAGE) or $gp41_{ECTO}$ and gp120 (reducing SDS-PAGE) were excised and washed five times, alternating between acetonitrile and water. The proteins in the gel bands were then digested *in situ* with PNGase F, at 37°C for 16 h, according to the manufacturer's instructions (New England Biolabs). The released glycans were extracted from the gel by washing with water, dried down in a SpeedVac concentrator and then either fluorescently labelled or subjected to IM-ESI MS.

Fluorescent labelling of N-linked glycans and HILIC-UPLC

Released glycans were fluorescently labelled with 2-aminobenzoic acid (2-AA) as previously described (Neville et al., 2009; Pritchard et al., 2015). Excess label was removed using Spe-ed Amide 2 cartridges (iBiosys Solutions Ltd), as described before (Neville et al., 2009). 2-AA labelled glycans were separated by HILIC-UPLC using a 2.1 mm x 10 mm Acquity BEH glycan column (1.7 µm particle size, Waters, Elstree, UK) in a Waters Acquity UPLC instrument. Glycans were separated by running the following gradient: time = 0 min (t = 0): 22 % A, 78 % B (flow rate 0.5 ml/min); t = 38.5: 44.1 % A, 55.9 % B (0.5 ml/min); t = 39.5: 100 % A, 0 % B (0.25 ml/min); t = 44.5: 100 % A, 0 % B; t = 46.5: 22 % A, 78 % B (0.25 ml/min), t = 46.6: 22 % A, 22 % B (0.5 ml/min), where solvent A was 50 mM ammonium formate, pH 4.4, and solvent B was acetonitrile. Fluorescence measuring occurred at an excitation wavelength of 250 nm and a detection wavelength of 428 nm. Empower 3 software was used for data processing.

Endo H digestion of released glycans

Digestion of released glycans with Endo H (New England Biolabs) was employed to measure the abundance of oligomannose-type glycans. The 2-AA labelled glycans were resuspended in water and digested with Endo H for 16 h at 37°C, according to the manufacturer's instructions, then extracted using a PVDF protein-binding membrane plate (Merck Millipore), again according to the manufacturer's instructions, and finally analysed by HILIC-UPLC. The relative abundance of oligomannose-type glycans was determined by integrating the chromatograms with or without Endo H, following normalization.

Tandem ion mobility-ESI MS analysis of released N-linked glycans

The total pool of released glycans from BG505 SOSIP.664 trimers (from the stable HEK 293T cell line) was analysed using ion mobility MS. These results were the basis for the creation of a glycan library that was then used for the subsequent site-specific N-glycosylation analyses. Glycan samples were

desalted with a Nafion membrane for 2 h, as described elsewhere (Börnsen et al., 1995). They were then dissolved in a solution of methanol-water (1:1; vol/vol) containing 100 mM ammonium phosphate. Negative ion mass, collision-induced dissociation (CID) and ion mobility spectra were recorded with a Waters Synapt G2Si mass spectrometer (Waters Corp.) fitted with a nano-electrospray ion source. Samples were infused through Waters thin-wall nanospray capillaries. The instrument was set up as follows: Electrospray capillary voltage: 1.2 kV; cone voltage: 150 V; ion source temperature: 80°C; ion mobility gas (nitrogen) flow: 80 mL/min; T-wave velocity: 450 m/sec; T-wave peak height: 40 V; trap DC bias: 60 V. CID fragmentation was performed after mobility separation in the transfer cell with argon as the collision gas. The collision cell voltage (60-130 V) was adjusted according to the parent ion mass to give an even distribution of fragment ions across the mass range. The instrument was externally mass-calibrated with glucose oligomers (Glc_{2-13}) from dextran (*Leuconostoc mesenteroides*). Samples were digested with α-Neuraminidase (*Clostridium perfringens;* New England Bioscience) if needed. Data acquisition and processing were carried out using the Waters Driftscope (version 2.8) software and MassLynx™ (version 4.1). Spectra were interpreted as described in earlier publications (Harvey, 2005a, b; Harvey, 2005c; Harvey et al., 2008).

Proteolytic digestion of BG505 SOSIP.664 and glycopeptide enrichment

A 200-300 µg sample of trimer in 50 mM Tris/HCl, 6 M Urea, pH 8.0 was used for proteolytic digestions. DTT was added to a final concentration of 5 mM during a 1h incubation at 50°C, followed by addition of 20 mM iodacetamide (IAA), for a further 1h at room temperature (RT) in the dark. Any residual IAA was neutralized by adding 20 mM DTT for 1h at RT. The protein was then buffer exchanged into 50 mM Tris/HCl, pH 8.0 using Vivaspin columns and digested with either trypsin or chymotrypsin (Mass Spectrometry Grade, Promega) at a ratio of 1:20 (w/w) or 1:30 (w/w), respectively, according to the manufacturer's instructions. Protease-digested samples were enriched for glycopeptides by using the ProteoExtract Glycopeptide Enrichment Kit (Merck Millipore) and

dried down in a SpeedVac concentrator. Glycopeptides were then either directly analysed by LC-ESI MS or fractionated by RP-HPLC and subjected to MALDI-TOF MS.

RP-HPLC fractionation of tryptic glycopeptides

Glycopeptides were resuspended in PBS buffer and fractionated by reverse phase-HPLC using a Jupiter C18 5 µm 250 x 4.5 mm column (300 Å, Phenomenex) and a Dionex U3000 LC system. The following gradient was run at a flow rate of 1 ml/min: time = 0 min (t = 0): 95 % Am 5 % B; t = 5: 95 % A, 5 % B; t = 90: 10 % A, 90 % B; t = 95: 10 % A, 90 % B; t = 97: 95 % A, 5 % B, where solvent A was 10 mM ammonium formate, pH 4.4 and solvent B was 10 mM ammonium formate in 80 % acetonitrile. Fractions were collected every minute for 90 min, dried down using a SpeedVac concentrator and resuspended in 20 mM $NaHCO_3$ buffer.

MALDI-TOF MS analysis of glycopeptides

Glycopeptide fractions were divided into two equal fractions. One half was directly analysed MALDI-TOF MS whereas the other was deglycosylated using PNGase F (New England Biolabs). Samples were desalted employing C18 Ziptips according to the manufacturer's instructions and then spotted onto a ground steel target plate in 2,5-dihydrobenzoic acid (DHB, 10 mg/ml in 50 % acetonitrile) matrix. MALDI-TOF MS was performed using an Autoflex^{TM} Speed MALDI-TOF(/TOF) instrument (Bruker), operated in positive ion mode. Tandem MS (MS/MS) was performed on both glycopeptides and peptides to confirm peptide identity. In cases where the same peptide eluted in more than one fraction, the relevant fractions were pooled and reanalysed. Acquired data were processed using DataAnalysis 3 software (Bruker). After baseline subtraction, a signal-to-noise ratio cut-off of 3 was applied. Peptides were manually identified by a combination of 2 approaches. MALDI-TOF MS/MS data were examined for the presence of characteristic glycopeptide fragment ions such as, for example, the $^{0.2}$X ion $[M_{peptide}+83+H]^+$ or the Y1-ion $[M_{peptide}+203+H]^+$ which then enabled the mass of the peptide portion to be calculated (Wuhrer et al., 2007). In addition, MS and MS/MS data of the corresponding deglycosylated peptide-containing fractions were included in the peptide

identification process. The following peptide modifications were considered: Carbamidomethylation of cysteine, oxidation of methionine, N-terminal Pyro-Glu conversion and deamidation of asparagine, as well as sodium and potassium adducts. As a next step, the glycopeptide spectra were screened for plausible glycopeptide compositions based on the mass of the peptide portion and the generated glycan library. The relative abundance of different glycoforms on each peptide was determined by integrating the intensity of all isotopic peaks, and relating this value to the sum of the intensities of all identified glycoforms.

LC-ESI MS and MS/MS analysis of glycopeptides

Enriched glycopeptides were analysed on a Q-Exactive Orbitrap mass spectrometer (Thermo Fisher Scientific) and glycopeptides were desalted using a Dionex UltiMate 300 RS pump with Acclaim PepMap C18 300 µm ID x 5 mm trap at RT. A flow rate 12 µL/min was used for 4 min using 0.05 % TFA (trifluoroacetic acid) in water as desalting buffer. Desalted peptides were separated using a Dionex UltiMate3000 RSLCnano pump with 75 µm ID x 500 mm analytical columns at 50 ^{o}C. A flow rate of 250 nl/min was used and peptides were eluted using a 2 h linear gradient of 4 % to 40 % buffer B over 120 min. Buffer A was 0.1 % formic acid in water and buffer B was 0.1 % formic acid in acetonitrile. MS data were acquired with XCalibur 3.0.63 (Thermo Fisher Scientific). A Top10 data dependent acquisition method was used. This method allows the selection, fragmentation and detection of ten precursors in a duty cycle time of 1.42 s. Higher energy collisional dissociation (HCD) fragmentation of glycopeptides was performed and led to the generation of low-molecular-weight oxonium ions, peptide fragmentation ions (predominantly y- and b-ions) and B- and Y-type fragmentation of glycosidic linkages (Cao et al., 2014). Data analysis and glycopeptide identification were performed using Byonic^{TM} (Version 2.7) and Byologic^{TM} software (Version 2.3; Protein Metrics Inc.). First, acquired tandem MS spectra were analysed with Byonic, considering the same peptide modifications as for MALDI-TOF MS analysis, a precursor mass tolerance of 4 ppm, a fragment mass tolerance of 10 ppm and two missed cleavages. For tryptic-digested samples, semi-tryptic cleavages

were allowed. Chymotrypsin digestions were specified by C-terminal cleavage of Tyr, Phe, Trp, Leu and Met. The personalized glycan library was used in order to identify the compositions of glycosylated peptides. Byologic was then used for further data processing including relative quantification. Fragmentation spectra of identified glycopeptides were manually verified. The abundance of individual glycoforms was determined by adding up the intensities of the extracted-ion chromatograms (XICs) over all charge states. Relative abundance = [Intensity of one glycoform] / [Intensities of all glycoforms identified on the same peptide].

Surface Plasmon Resonance

Binding of Fabs was analysed by surface plasmon resonance (SPR) on a Biacore 3000 instrument at 25 °C. Env trimers (transiently expressed in HEK 293T cells and purified by 2G12 affinity chromatography and SEC (Lee et al., 2015)) were immobilized by antibody binding to a His-tag, as previously described (Lee et al., 2015; Yasmeen et al., 2014). Briefly, anti-histidine antibody (GE Healthcare) was amide-coupled to the dextran surface of a CM5 chip up to an immobilization level of 15000 RU. After deactivation, Env trimers were captured to yield R_L values of 520 RU (<2 % s.d.); HBS-EP (10 mM HEPES [pH 7.4], 150 mM NaCl, 3 mM EDTA, 0.002 % P20 surfactant) was used as running buffer. A high flow rate for analyte injections (50 µl/min) was used in order to prevent significant distortion by mass-transport limitation, the near absence of which was confirmed by global fits of mass transfer coefficients. After each cycle the capture-antibody surface was regenerated by an injection of 10 mM Glycine (pH 2) for 120 s at a flow rate of 30 µl/min. The signals from both 0-analyte and control-channel injections were subtracted. A Langmuir model (Biaevaluation, GE Healthcare) was fitted to the binding data as previously described (Yasmeen et al., 2014). S_m values were based on the highest analyte concentrations. All reported binding parameters were significant (T>10).

Supplemental References

Börnsen, K.O., Mohr, M.D., and Widmer, H.M. (1995). Ion exchange and purification of carbohydrates on a Nafion® membrane as a new sample pretreatment for matrix-assisted laser desorption/ionization mass spectrometry. Rapid Commun Mass Spectrom *9*, 1031-1034.

Cao, L., Tolic, N., Qu, Y., Meng, D., Zhao, R., Zhang, Q., Moore, R.J., Zink, E.M., Lipton, M.S., Pasa-Tolic, L., *et al.* (2014). Characterization of intact N- and O-linked glycopeptides using higher energy collisional dissociation. Anal Biochem *452*, 96-102.

Chung, N.P.Y., Matthews, K., Kim, H.J., Ketas, T.J., Golabek, M., de Los Reyes, K., Korzun, J., Yasmeen, A., Sanders, R.W., Klasse, P.J., *et al.* (2014). Stable 293 T and CHO cell lines expressing cleaved, stable HIV-1 envelope glycoprotein trimers for structural and vaccine studies. Retrovirology *11*, 33-33.

Harvey, D.J. (2005a). Fragmentation of negative ions from carbohydrates: part 1. Use of nitrate and other anionic adducts for the production of negative ion electrospray spectra from N-linked carbohydrates. J Am Soc Mass Spectrom *16*, 622-630.

Harvey, D.J. (2005b). Fragmentation of negative ions from carbohydrates: part 2. Fragmentation of high-mannose N-linked glycans. J Am Soc Mass Spectrom *16*, 631-646.

Harvey, D.J. (2005c). Fragmentation of negative ions from carbohydrates: part 3. Fragmentation of hybrid and complex N-linked glycans. J Am Soc Mass Spectrom *16*, 647-659.

Harvey, D.J., Merry, A.H., Royle, L., Campbell, M.P., and Rudd, P.M. (2011). Symbol nomenclature for representing glycan structures: Extension to cover different carbohydrate types. Proteomics *11*, 4291-4295.

Harvey, D.J., Royle, L., Radcliffe, C.M., Rudd, P.M., and Dwek, R.A. (2008). Structural and quantitative analysis of N-linked glycans by matrix-assisted laser desorption ionization and negative ion nanospray mass spectrometry. Anal Biochem *376*, 44-60.

Lee, J.H., Leaman, D.P., Kim, A.S., Torrents de la Peña, A., Sliepen, K., Yasmeen, A., Derking, R., Ramos, A., de Taeye, S.W., Ozorowski, G*., et al.* (2015). Antibodies to a conformational epitope on gp41 neutralize HIV-1 by destabilizing the Env spike. Nature communications *6*, 8167-8167.

Li, M., Gao, F., Mascola, J.R., Stamatatos, L., Polonis, V.R., Koutsoukos, M., Voss, G., Goepfert, P., Gilbert, P., Greene, K.M*., et al.* (2005). Human immunodeficiency virus type 1 env clones from acute and early subtype B infections for standardized assessments of vaccine-elicited neutralizing antibodies. J Virol *79*, 10108-10125.

Montefiori, D.C. (2005). Evaluating neutralizing antibodies against HIV, SIV, and SHIV in luciferase reporter gene assays. Curr Protoc Immunol *12*, 11.11-11.17.

Neville, D.C., Dwek, R.A., and Butters, T.D. (2009). Development of a single column method for the separation of lipid- and protein-derived oligosaccharides. J Proteome Res *8*, 681-687.

Pritchard, L.K., Vasiljevic, S., Ozorowski, G., Seabright, G.E., Cupo, A., Ringe, R., Kim, H.J., Sanders, R.W., Doores, K.J., Burton, D.R*., et al.* (2015). Structural constraints determine the glycosylation of HIV-1 envelope trimers. Cell Rep *11*, 1604-1613.

Ringe, R.P., Yasmeen, A., Ozorowski, G., Go, E.P., Pritchard, L.K., Guttman, M., Ketas, T.A., Cottrell, C.A., Wilson, I.A., Sanders, R.W*., et al.* (2015). Influences on the design and purification of soluble, recombinant native-like HIV-1 envelope glycoprotein trimers. J Virol *89*, 12189-12210.

Sanders, R.W., Derking, R., Cupo, A., Julien, J.P., Yasmeen, A., de Val, N., Kim, H.J., Blattner, C., de la Pena, A.T., Korzun, J*., et al.* (2013). A next-generation cleaved, soluble HIV-1 Env trimer, BG505 SOSIP.664 gp140, expresses multiple epitopes for broadly neutralizing but not non-neutralizing antibodies. PLoS Path *9*, e1003618.

Wuhrer, M., Catalina, M.I., Deelder, A.M., and Hokke, C.H. (2007). Glycoproteomics based on tandem mass spectrometry of glycopeptides. J Chromatogr B Analyt Technol Biomed Life Sci *849*, 115-128.

Yasmeen, A., Ringe, R., Derking, R., Cupo, A., Julien, J.P., Burton, D.R., Ward, A.B., Wilson, I.A., Sanders, R.W., Moore, J.P.*, et al.* (2014). Differential binding of neutralizing and non-neutralizing antibodies to native-like soluble HIV-1 Env trimers, uncleaved Env proteins, and monomeric subunits. Retrovirology *11*, 41.
